# Circular RNA hsa_circRNA_0007334 is Predicted to Promote MMP7 and COL1A1 Expression by Functioning as a miRNA Sponge in Pancreatic Ductal Adenocarcinoma

**DOI:** 10.1155/2019/7630894

**Published:** 2019-07-24

**Authors:** Jinghui Yang, Xianling Cong, Ming Ren, Hongyan Sun, Tao Liu, Gaoyang Chen, Qingyu Wang, Zhaoyan Li, Shan Yu, Qiwei Yang

**Affiliations:** ^1^Department of Hepatopancreatobiliary Surgery, China-Japan Union Hospital of Jilin University, Changchun 130033, China; ^2^Tissue Bank, China-Japan Union Hospital of Jilin University, Changchun 130033, China; ^3^Department of Orthopedics, Second Hospital of Jilin University, Changchun 130041, China; ^4^Second Breast Department of Jilin Cancer Hospital, Changchun 130021, China; ^5^Department of Neurology, China-Japan Union Hospital of Jilin University, Changchun 130033, China; ^6^Medical Research Center, Second Hospital of Jilin University, Changchun 130041, China

## Abstract

Pancreatic cancer remains one of the leading causes of cancer-related deaths worldwide. Pancreatic ductal adenocarcinoma (PDAC) is the most common type of pancreatic tumor. Many circular RNAs (circRNAs) have proven to play vital roles in the physiological and pathological processes of tumorigenesis; however, their biogenesis in PDAC remains unclear. In this study, the expression profiles of circRNAs from 10 PDAC tissues and their paired adjacent nontumor tissues were analyzed through RNA sequencing analysis. An enrichment analysis was employed to predict the functions of the differentially expressed circRNAs. Sequence alignment information and mRNA microarray projects were used to predict the RNA regulatory network. The knockdown of circRNAs by small interfering RNAs followed by wound healing and western blot assays was used to confirm their functions in a PDAC cell line. A total of 278 circRNAs were identified as differentially expressed in PDAC tissue. Of these, we found that hsa_circRNA_0007334 was significantly upregulated and may serve as a competing endogenous RNA to regulate matrix metallopeptidase 7 (MMP7) and collagen type I alpha 1 chain (COL1A1) by the competitive adsorption of hsa-miR-144-3p and hsa-miR-577 to enhance the expression and functions of MMP7 and COL1A1 in PDAC. In vitro experiments confirmed these results. The present study is the first to propose two regulatory pathways in PDAC: hsa_circRNA_0007334–hsa-miR-144-3p–MMP7 and hsa_circRNA_0007334–hsa-miR-577–COL1A1.

## 1. Introduction

Pancreatic cancer remains one of the leading causes of cancer-related deaths worldwide. The number of global deaths caused by pancreatic cancers of all types was 411,600 in 2015 [1]. Statistics collected by the American Cancer Society show that 12.5 of 100,000 people, on average, are at risk of pancreatic cancer and 10.9 of 100,000 people die from pancreatic cancer. Pancreatic ductal adenocarcinoma (PDAC) is the most common type of pancreatic tumor, accounting for approximately 85%-90% of all pancreatic tumors. It has now become the seventh leading cause of cancer-related deaths in the world [2].

Noncoding RNAs, including circular RNAs (circRNAs), long noncoding RNAs (lncRNAs), and microRNAs (miRNAs), all play vital roles in physiological and pathological processes, as suggested in a growing number of studies [3–7]. Although the function of circRNAs as regulators in the process of tumorigenesis has been previously acknowledged, their biogenesis and potential functions remain difficult to understand. The competing endogenous RNA (ceRNA) mechanism is regarded as one of the most important mechanisms by which circRNAs regulate gene expression [8]. In theory, circRNAs function as molecular sponges for a miRNA to bind to its binding site, miRNA response elements (MREs), so that the expression and function of all target genes of the respective miRNA family are suppressed [9–11]. Nevertheless, the role of circRNAs in PDAC initiation and progression is unknown.

In the present study, we analyzed the expression profiles of circRNAs in PDAC tissue through RNA sequencing to further investigate differentially expressed circRNAs and their biological roles in PDAC. Our findings indicate that hsa_circRNA_0007334 promotes the expression levels of matrix metallopeptidase 7 (MMP7) and collagen type I alpha 1 chain (COL1A1) by blocking the functions of miR-144-3p and miR-577 in PDAC. We propose two regulatory pathways in PDAC: hsa_circRNA_0007334–hsa-miR-144-3p–MMP7 and hsa_circRNA_0007334–hsa-miR-577–COL1A1. The circular RNA hsa_circRNA_0007334 can also be used as a potential biomarker in PDAC diagnosis and therapy.

## 2. Materials and Methods

### 2.1. Tissue Specimens

Ten PDAC tissues and ten paired adjacent nontumor tissues were provided by the Tissue Bank of China-Japan Union Hospital, Jilin University (Changchun, China). The clinical information of the patients is shown in Table 1.

### 2.2. Ethics Approval and Informed Statement

The Ethics Committee of the China-Japan Union Hospital of Jilin University was given detailed study information and approved all experimental protocols (approval number: 2018-NSFC-006). All participants were informed of the use of their specimens, and written informed consent was obtained from each participant.

### 2.3. RNA Preparation

Total RNA was extracted using TRIzol reagent (Invitrogen, USA) according to the manufacturer's instructions. The concentration and purity of the isolated RNA were determined by a NanoDrop 2000 instrument (Thermo Scientific, USA) by measuring the absorbance values at 260 nm (A260) and 280 nm (A280).

### 2.4. Library Preparation for circRNA Sequencing

Five PDAC tissues and five paired adjacent nontumor tissues were employed for the circRNA sequencing analysis. A total of 5 *μ*g of RNA per sample was used as input material for RNA sample preparation. Ribosomal RNAs were depleted by an Epicentre Ribo-Zero rRNA Removal Kit (Epicentre, USA). rRNA-depleted RNAs were treated with RNase R (Epicentre, USA) and then subjected to TRIzol extraction. Sequencing libraries were generated using the rRNA-depleted and RNase R-digested RNAs with an NEBNext Ultra Directional RNA Library Prep Kit for Illumina (NEB, USA) following the manufacturer's recommendations. The remaining steps were performed according to the descriptions in previous articles [12, 13]. The library was purified (AMPure XP system) and then qualified on an Agilent Bioanalyzer 2100 system.

### 2.5. Clustering and Sequencing

Clustering of the index-coded samples was performed on a cBot Cluster Generation System using a HiSeq PE Cluster Kit v4 cBot (Illumina) according to the manufacturer's instructions. The library preparations were sequenced on an Illumina HiSeq 2500 platform, and 125 bp paired-end reads were generated. Fold change values were calculated by the negative binomial distribution DESeq2 method using the expression level (read count data). We calculated the p value between the data of two groups by negative binomial distribution and corrected the multiple hypothesis by Benjamini-Hochberg (BH) correction to obtain the adjusted p value.

### 2.6. Enrichment Analysis

Gene Ontology (GO) enrichment analysis was performed by GOseq (version 1.18.0) [14] to determine the parental gene function of the differentially expressed circRNAs. GO terms with a corrected p value less than 0.05 were considered significantly enriched by the differentially expressed genes. KOBAS [15] was used for Kyoto Encyclopedia of Genes and Genomes (KEGG) pathway enrichment analysis.

### 2.7. Reverse Transcription Quantitative PCR

For mRNA and circRNA, 1 *μ*g of total RNA was used for cDNA synthesis using EasyScript One-Step gDNA Removal and cDNA Synthesis SuperMix (TransGen Biotech, China) and for RT-qPCR analysis using TransStart Top Green qPCR SuperMix (TransGen Biotech, China), according to the manufacturer's instructions. The primers were designed by Primer Premier 5 and synthesized by Sangon Biotech, Inc. (China). The primer sequences are as follows: hsa_circRNA_0007334 exon sequence, forward: 5′-ATCATCATAGGAGTGGAGAA-3′, reverse: 5′-TAGCAAGGCAAAGAGTTG-3′; hsa_circRNA_0007334 potential intron sequences, forward: 5′-CTGGGAATACTGTGTTATGC-3′, reverse: 5′-TACATCCAAATAGTCTTCATT-3′; MMP7, forward: 5′-GCATCTCCTTGAGTTTGGCT-3′, reverse: 5′-GAGCTACAGTGGGAACAGGC-3′; COL1A1, forward: 5′-CACACGTCTCGGTCATGGTA-3′, reverse: 5′-AAGAGGAAGGCCAAGTCGAG-3′; and GAPDH, forward: 5′-GACAGTCAGCCGCATCTTCT-3′, reverse: 5′-TTAAAAGCAGCCCTGGTGAC-3′.

For miRNA, 500 ng of total RNA was used for cDNA synthesis and for RT-qPCR analysis on an All-in-One™ miRNA RT-qPCR Detection System (GeneCopoeia, USA) according to the manufacturer's instructions. The primers were purchased from GeneCopoeia (USA) (hsa-miR-144-3p: Cat. #HmiRQP0190; hsa-miR-577: Cat. #HmiRQP0678).

RT-qPCR analysis was performed on a 7500 Fast Dx Real-Time PCR Instrument (Applied Biosystems, USA). RT-qPCR was repeated three times for each sample. The cycle threshold value (Ct) data were analyzed using the 2^−ΔΔCt^ method.

### 2.8. Knockdown of hsa_circRNA_0007334 by a Small Interfering RNA (siRNA)

PANC-1 cells were purchased from the National Infrastructure of Cell Line Resource of China and cultivated in DMEM basic (Gibco, USA) containing 10% fetal bovine serum (Gibco, USA). The cells were adjusted to a density of 1 × 10^5^ cells per well in 24-well plates and incubated at 37°C in a 5% CO_2_ humidified atmosphere. After 24 hours, 50 nM siRNA transfection reagent was made according to the manufacturer's instructions and added to the cells. After an additional 24 hours, RNA and protein were extracted from the cells. The cells were divided into 4 groups: the si-circRNA_0007334 group was transfected with si-hsa_circRNA_0007334, the si-circRNA_0007334+miR-144-3p inhibitor group was transfected with both si-hsa_circRNA_0007334 and the hsa-miR-144-3p inhibitor, the si-circRNA_0007334+miR-577 inhibitor group was transfected with both si-hsa_circRNA_0007334 and the hsa-miR-577 inhibitor, and the negative control (NC) group was treated with only transfection reagents in the absence of the siRNA or miRNA inhibitors. The interfering RNA target sequence of hsa_circRNA_0007334 was 5′-GGAGAACATGCACAAGTCA-3′. The siRNA (Cat. #siG180808051115), miR-144-3p inhibitor (Cat. #miR20000436), hsa-miR-577 (Cat. #miR20003242), and transfection reagents (ribo FECT™ CP Transfection Kit Cat. # C10511-05) were purchased from RiboBio Co., Ltd. (China) and operated according to the manufacturer's instructions.

### 2.9. Wound Healing Assay

A wound healing assay was performed to detect the invasion ability of PANC-1 cells after the knockdown of hsa_circRNA_0007334. A Culture-Insert 2 Well (ibidi, Germany) was used to prepare the scratch in 24-well plates. The Culture-Insert 2 Well was removed after 24 hours, and hsa_circRNA_0007334 knockdown was immediately performed as previously described. Microscopic cell images were collected at 0, 6, 12, and 24 hours after knockdown. The migration rate was analyzed by ImageJ software based on the scratch areas.

### 2.10. Western Blot Analysis

RIPA lysis buffer (Beyotime, China) containing protease inhibitor cocktail (Beyotime, China) was used to extract total protein according to the manufacturer's instructions. Fifty micrograms of total protein from each sample was used for SDS-PAGE and then transferred to PVDF membranes (Millipore, China). The antibodies used were anti-MMP7 (1:1000, ab189277, Abcam, USA), anti-COL1A1 (1:1000, ab6308, Abcam, USA), and anti-GAPDH (1:5000, ab245, Abcam, USA). A luminescent and fluorescent biological image analysis system (Furi Science & Technology, China) was used to detect exposure after adding the enhanced chemiluminescent (ECL) reagent. Images were analyzed by ImageJ software to calculate relative expression levels.

### 2.11. Statistical Analysis

The quantitative data are shown as the mean±standard deviation (SD). Statistical analyses were performed using SPSS version 21 (SPSS, Inc., USA). Student's t-test was used to compare the expression levels of hsa_circRNA_0007334, MMP7, and COL1A1. Two-tailed Pearson's correlation analysis was used to determine the association between the expression levels of hsa-miR-144-3p and hsa-miR-577. P<0.05 indicates a statistically significant difference. Genes with a fold change ≥ 2 and a p value < 0.05 in the sequencing or microarray data were regarded as significantly differentially expressed.

## 3. Results

### 3.1. circRNA Expression Profiles in PDAC

**Figure 1:**
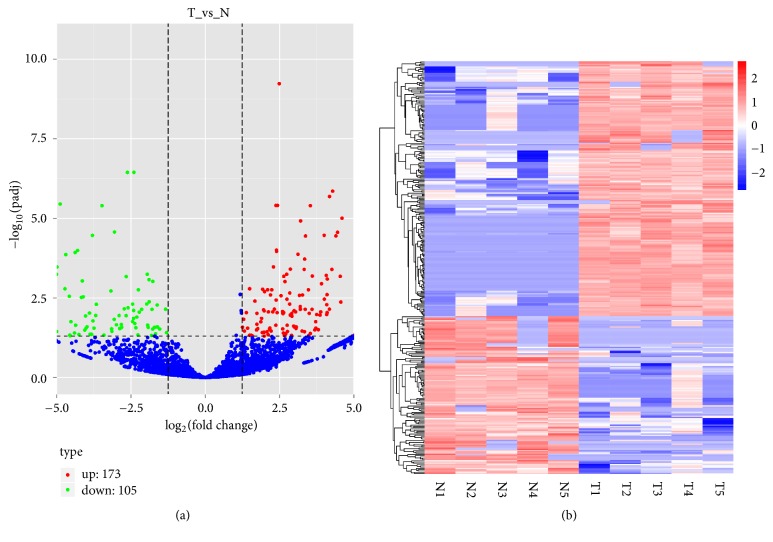
circRNA expression profile in PDAC. (a) The volcano plot was constructed according to the fold change and p value. The x-axis presents the log_2_(fold change) value of differential expression, and the y-axis presents the -log_10_(padj) value of differential expression. The vertical lines correspond to 2.0-fold up- and downregulation between PDAC and adjacent nontumor tissues, and the horizontal line represents a p value of 0.05. The red circles in the plot represent the upregulated circRNAs, and the green circles in the plot represent the downregulated differentially expressed circRNAs with statistical significance. (b) Hierarchical clustering analysis of circRNAs that were differentially expressed (over a 2.5-fold change) between PDAC and adjacent nontumor tissues. The x-axis presents samples (N1-N5: adjacent nontumor tissues; T1-T5: PDAC tissues), and the y-axis presents the differentially expressed circRNAs. Expression values are represented in different colors; red indicates a high expression level, and blue indicates a low expression level.

The circRNA expression profiles in PDAC tissues and adjacent nontumor tissues (5 PDAC tissues and 5 adjacent nontumor tissues) were revealed by hierarchical clustering. The variation in circRNA expression between PDAC tissues and adjacent nontumor tissues is presented in a volcano plot (Figure 1(a)). Overall, 28,374 circRNAs were identified. The information on read mapping, quality filtering, data normalization, and the analysis of differential expression is shown in Supplementary Data  1. Among these circRNAs, 278 were identified as differentially expressed (fold change ≥ 2.0 and p < 0.05). Of these, 173 circRNAs were identified as upregulated, and 105 circRNAs were identified as downregulated (Supplementary Data  2). The differentially expressed circRNAs with a fold change ≥ 2.5 are presented as a cluster heatmap (Figure 1(b)).

### 3.2. Enrichment Analysis of Parental Gene Function of the Differentially Expressed circRNAs

**Figure 2:**
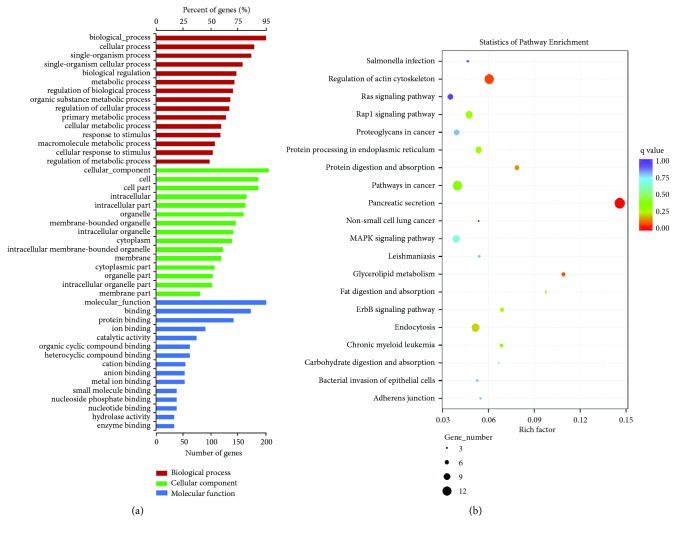
GO and KEGG analyses of the parental gene function of the differentially expressed circRNAs. (a) GO analysis covers the three domains of biological process, cellular component, and molecular function. The x-axis presents the gene number and the proportion of all annotated genes, and the y-axis presents the top 15 functions in each term. (b) KEGG analysis revealed the most important biochemical metabolic pathways and signal transduction pathways in which the genes are involved. The x-axis presents the Rich factor in this pathway, the y-axis presents the related pathways, the q values are represented in different colors, and the size of the circle represents the number of genes in this pathway. The degree of KEGG enrichment is measured by the Rich factor, the q value, and the number of genes in this pathway. The Rich factor refers to the ratio of the number of differentially expressed genes located in the pathway to the total number of genes in the pathway. The larger the Rich factor, the greater the degree of enrichment. The q value is the p value after the multiplicative hypothesis test and correction, and the value range of the q value is 0-1. The closer the value to 0, the more significant the enrichment.

According to the correspondence between the circRNA and its parental gene, GO and KEGG enrichment analyses were performed to determine the parental gene function of the differentially expressed circRNAs. GO classification, mainly including biological process, cellular component, and molecular function, was applied to analyze the main functions of the differentially expressed genes according to GO terms. The gene number and the proportion of all annotated genes that are related to the top 15 functions in each term are shown in Figure 2(a). Through GO analysis, we obtained 217 parental genes of circRNAs that are closely related to the development of PDAC (Supplementary Data  3). These parental genes can help us further understand which genes are affected in PDAC tissue, and at the same time, we can predict which circRNAs regulate these genes. Furthermore, we can predict the potential functions of the circRNAs. Significant enrichment by pathway analysis can reveal the most important biochemical metabolic pathways and signal transduction pathways in which the genes are involved. KEGG is the main public database on pathways (http://www.genome.jp/kegg/). KEGG pathway analysis can be used to identify pathways that are significantly enriched in the gene of interest. A KEGG pathway scatterplot is a graphical way to present KEGG enrichment analysis results (Figure 2(b)). The 20 most prominent enrichment pathways are shown in the figure.

### 3.3. Construction of the ceRNA Network of the Differentially Expressed circRNAs

**Figure 3:**
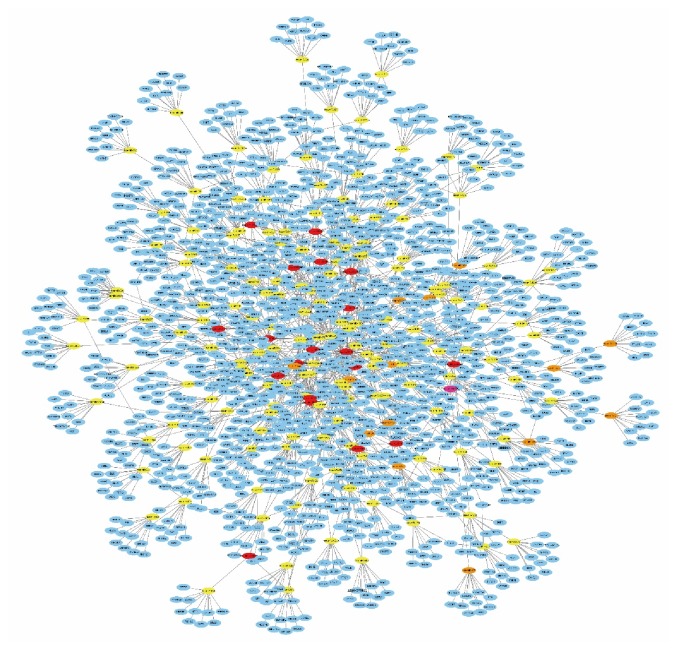
ceRNA analysis of the differentially expressed circRNAs. Ten circRNAs (red nodes), 173 miRNAs (yellow nodes), and 1,460 target genes (blue nodes) are involved in the ceRNA network. Solid lines represent the relationship between two nodes.

To predict the functions of the differentially expressed circRNAs, we constructed a ceRNA network with Cytoscape. The ten circRNAs with the largest fold change (5 upregulated and 5 downregulated) and the ten circRNAs with the smallest p value were chosen to construct the network. To enhance data reliability, the circRNAs that have not been recorded in ENCODE were removed. First, we used the Circular RNA Interactome database (https://circinteractome.nia.nih.gov/) to predict all the miRNAs that are most likely to be associated with the chosen circRNAs. Then, we used TargetScan (http://www.targetscan.org/vert_71/) to predict the target genes. TargetScan allows miRNA-mRNA predictions based on solely the presence of seed sequences, which is considered the main influence on the biological function of a mRNA. All predicted target genes were ranked according to the total context score, and only the top 10 target genes were selected to construct the network. After removing duplicate items, there were a total of 173 miRNAs and 1,460 target genes in the ceRNA network (Figure 3). The ceRNA network shows that circRNAs can interact with target genes through miRNAs by sequence complementation. This ceRNA network map visually shows the complex regulatory relationships between various RNAs. One circRNA can regulate a large number of target genes, or one target gene can be regulated by multiple circRNAs. In contrast, among the 20 differentially expressed circRNAs, the ceRNA network of hsa_circRNA_0007334 is relatively simple and clear. Moreover, the parental gene of hsa_circRNA_0007334 (MBOAT2, ENSG00000143797) was associated with 23 of the 45 biological functions involved in the GO analysis.

### 3.4. The Expression Profile of hsa_circ_0007334

The expression level of hsa_circRNA_0007334 in PDAC tissues was upregulated 4.18-fold. Although hsa_circRNA_0007334 was not the most differentially expressed, the significance of its difference ranked third among all the circRNAs. To verify the sequencing results, RT-qPCR was performed. The relative expression level results showed that the expression of hsa_circRNA_0007334 was upregulated 3.92-fold in PDAC tissues compared with adjacent nontumor tissues. According to the sequencing results and the human reference genome (GRCh37/hg19) obtained from the University of California at Santa Cruz (UCSC) genome database (http://genome.ucsc.edu/), we presume that hsa_circRNA_0007334 has a genomic length of 15,456 nt and a spliced length of 224 nt. Its location in the genome is chr2:9083315-9098771. The mature sequence of hsa_circRNA_0007334 is GTCAACTTTGTAGTGTGCCAACTCTTTGCCTTGCTAGCAGCCATTTGGTTTCGAACTTATCTACATTCAAGCAAAACTAGCTCTTTTATAAGACATGTAGTTGCTACCCTTTTGGGCCTTTATCTTGCACTTTTTTGCTTTGGATGGTATGCCTTACACTTTCTTGTACAAAGTGGAATTTCCTACTGTATCATGATCATCATAGGAGTGGAGAACATGCACAA, and its specific junction sequence is CATCATAGGAGTGGAGAACATGCACAAGTCAACTTTGTAGTGTGCCAACTCTTT.

### 3.5. Identification of Exon-Intron circRNAs (EIciRNAs)

To determine whether there is an EIciRNA form for hsa_circRNA_0007334, we designed primers based on the possible intron regions of hsa_circRNA_0007334 and performed reverse transcription PCR according to previous studies [16]. We designed two forward primers in the exon region (marked as the F-primer) and intron region (marked as the F′-primer) and two reverse primers in the exon region (marked as the R-primer) and intron region (marked as the R′-primer) (Supplementary Figure  1a). Then, the primers were paired with each other for PCR amplification using cDNA as a template. The agarose gel electrophoresis results showed that hsa_circRNA_0007334 could be effectively amplified only when both of the exonic forward and reverse primers were used, and the intronic primers could not amplify hsa_circRNA_0007334 (Supplementary Figure  1b). These results suggest that the intron sequence is not detected in mature hsa_circRNA_0007334 and that hsa_circRNA_0007334 is composed of exons only.

### 3.6. ceRNA Analysis of hsa_circRNA_0007334

**Figure 4:**
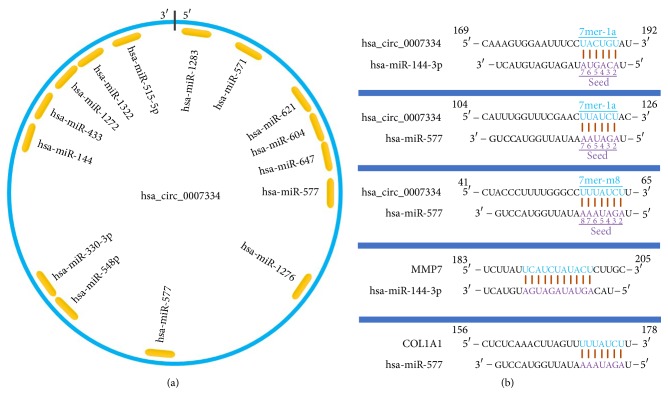
Pattern diagram of hsa_circRNA_0007334 and its miRNA binding sites. (a) A schematic model showing the putative miRNA binding sites for hsa_circRNA_0007334. The indigo circle represents the structure of hsa_circRNA_0007334, and the yellow fragments represent miRNAs. (b) Binding sites of the miRNAs hsa-miR-144-3p and hsa-miR-577 in circRNA hsa_circRNA_0007334 and binding sites of the miRNAs hsa-miR-144-3p and hsa-miR-577 in the target genes, MMP7 and COL1A1.

**Figure 5:**
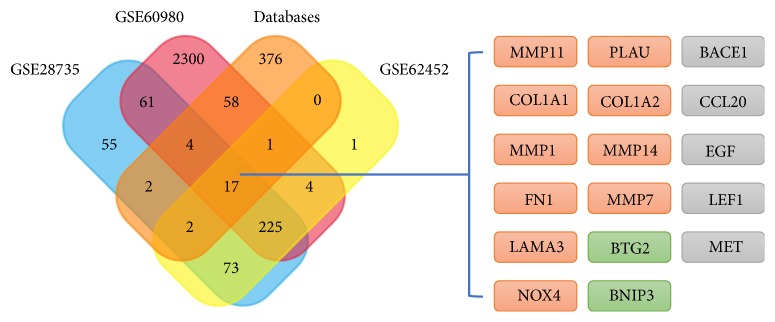
Venn analysis of the differentially expressed genes. The Venn diagram shows 17 genes mentioned in both the databases and 3 microarray projects. Among these 17 genes, MMP1, MMP7, MMP11, MMP14, COL1A1, COL1A2, FN1, LAMA3, NOX4, and PLAU are highly expressed in PDAC tissues (marked as orange, round rectangles); BTG2 and BNIP3 are weakly expressed in PDAC tissues (marked as green, round rectangles); BACE1, CCL20, EGF, LEF1, and MET are not expressed in pancreatic tumor according to the NCBI-UniGene database (marked as gray, round rectangles).

**Figure 6:**
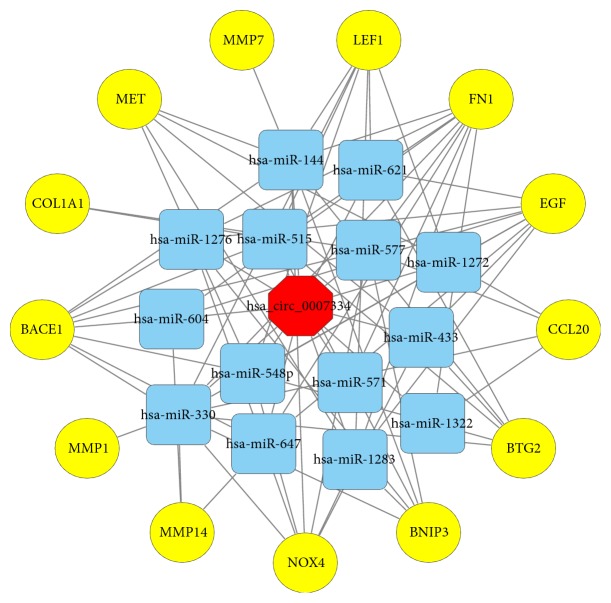
ceRNA analysis of hsa_circRNA_0007334. hsa_circRNA_0007334 (red octagon), 14 miRNAs (blue, round rectangles), and 13 target genes (yellow circles) are involved in the ceRNA network. Solid lines represent the relationship between two nodes.

Assuming that hsa_circRNA_0007334 functions in PDAC through a ceRNA mechanism, we performed the following analysis. First, we used the Circular RNA Interactome database (https://circinteractome.nia.nih.gov/) to predict the miRNAs that are most likely associated with hsa_circRNA_0007334. We found 15 potential miRNA binding sites that can bind with 14 miRNAs (Figure 4(a)). For hsa-miR-577, there are two suitable binding sites. The miRNAs predicted to bind to hsa_circRNA_0007334 are shown in Supplementary Data 4. Then, we summarized the results of the three databases, namely, HMDD (http://www.cuilab.cn/hmdd), miRWalk 2.0 (http://zmf.umm.uni-heidelberg.de/apps/zmf/mirwalk2/), and MalaCards (http://www.malacards.org/), to predict the miRNAs and their target genes that are related to pancreatic cancer. Next, we investigated three mRNA microarray projects (GSE28735, GSE60980, and GSE62452) from the Gene Expression Omnibus (GEO) DataSets (https://www.ncbi.nlm.nih.gov/gds/?term=) with 163 PDAC tissues and 118 adjacent nontumor tissues in total to identify genes that are differentially expressed between tumor and adjacent nontumor tissues in PDAC patients. The differential expression data of the mRNA microarray were auto-analyzed by the GEO2R online tool. Only the genes with a differential expression fold change ≥ 2.0 and p < 0.05 were further analyzed. A comprehensive comparison of the above results by Venn analysis revealed that 17 of 3,179 genes were differentially expressed in PDAC tumor tissue (Figure 5). Next, we predicted the miRNAs that can regulate the expression of these 17 genes by TargetScan (http://www.targetscan.org/vert_71/), miRanda (http://34.236.212.39/microrna/microrna/home.do), and RNA22 (https://cm.jefferson.edu/rna22/). Then, we overlapped the results with the 14 miRNAs mentioned earlier. All of these 14 miRNAs were further analyzed. Of the 17 predicted genes, 13 were predicted to be regulated by these 14 miRNAs and were further analyzed. Finally, we used hsa_circRNA_0007334, 14 miRNAs, and 13 target genes and constructed an hsa_circRNA_0007334-miRNA-target gene network using Cytoscape to visualize their interactions (Figure 6). It The ceRNA network revealed that hsa_circRNA_0007334 regulates the expression of MMP7 and COL1A1 through hsa-miR-144-3p and hsa-miR-577, respectively. Compared to other pathways, this pathway is relatively simple and less affected by other miRNAs. The 2D structures and binding sites of hsa_circRNA_0007334, hsa-miR-144-3p, hsa-miR-577, MMP7, and COL1A1 are illustrated in Figure 4(b).

### 3.7. Protein Function Analysis of the Predicted Target Genes

**Figure 7:**
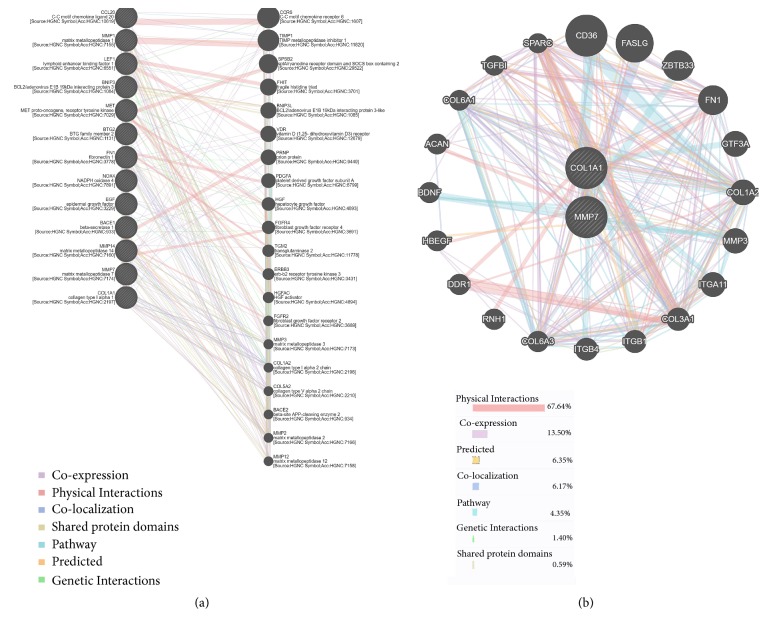
Protein-protein interaction (PPI) analysis of the predicted target genes. (a) The network shows the protein interactions between 13 predicted target genes and their related genes. (b) The network shows the protein interactions between MMP7, COL1A1, and their related genes. The gray nodes represent genes. The node size represents the correlation strength between genes. The line between two nodes represents the mode of action (color of the line) and strength (width of the line) between genes.

To verify the functions and interactions of these 13 target genes, we performed a protein-protein interaction (PPI) analysis with the GeneMANIA database (http://genemania.org/). The protein interactions between the predicted target genes and their related genes were constructed in the network, as shown in Figure 7(a). The analysis revealed that the interaction between these proteins is mainly through physical interactions. As CCR6 and TIMP1 interact with CCL20 or MMP1 via physical interactions, they also interact with MMP7 or COL1A1 via coexpression. Notably, there are several MMP families and collagen families included in this network. To further understand the protein interactions of the two genes of interest, MMP7 and COL1A1, the protein interactions between MMP7, COL1A1, and their related genes were separately constructed in the network, as shown in Figure 7(b). These genes may help explain the biological activity changes that occur when the expression levels of MMP7 and COL1A1 are changed.

### 3.8. Expression Profiles of the miRNAs hsa-miR-144-3p and hsa-miR-577 and MMP7 and COL1A1 mRNAs in PDAC

**Figure 8:**
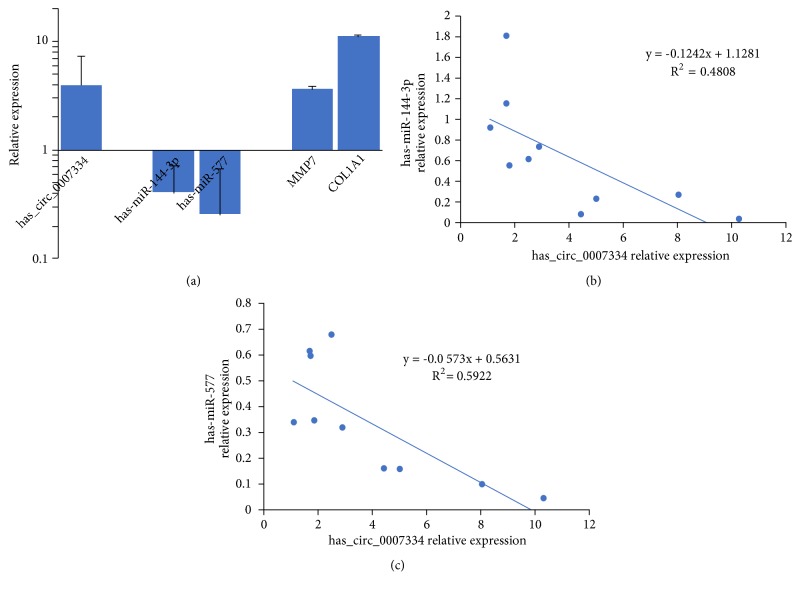
Expression profiles of the miRNAs hsa-miR-144-3p and hsa-miR-577 and MMP7 and COL1A1 mRNAs in PDAC. (a) The expression levels of the circRNA hsa_circRNA_0007334, the miRNAs hsa-miR-144-3p and hsa-miR-577, and MMP7 and COL1A1 mRNAs were analyzed by the 2^−ΔΔCt^ method using RT-qPCR. The x-axis presents the genes. The y-axis presents the relative expression level, and a relative expression level higher than 1.00 indicates that it is upregulated. In PDAC tissues, compared with adjacent nontumor tissues, a relative expression level lower than 1.00 indicates that it is downregulated. (b) Two-tailed Pearson's correlation analysis of the relationship between hsa_circRNA_0007334 and hsa-miR-144-3p expression. (c) Two-tailed Pearson's correlation analysis of the relationship between hsa_circRNA_0007334 and hsa-miR-577 expression.

To validate the results of the ceRNA analysis, RT-qPCR was further performed in 10 PDAC tissues and 10 adjacent nontumor tissues. The relative expression levels of the miRNAs hsa-miR-144-3p and hsa-miR-577 and MMP7 and COL1A1 mRNAs were measured by the 2^−ΔΔCt^ method. The expression levels in adjacent nontumor tissues were normalized to 1.00. In PDAC tissues, a relative expression level higher than 1.00 indicates that it is upregulated in PDAC tissues compared with adjacent nontumor tissues; in contrast, a relative expression level lower than 1.00 indicates that it is downregulated. As mentioned above, the expression of hsa_circRNA_0007334 was upregulated 3.92-fold in PDAC tissues compared with adjacent nontumor tissues. As expected, the expression of hsa-miR-144-3p and hsa-miR-577 in PDAC tissue was downregulated 2.50-fold and 3.85-fold, respectively, compared with that in adjacent nontumor tissues. Correspondingly, the expression of MMP7 and COL1A1 in PDAC tissue was upregulated 3.69-fold and 11.31-fold, respectively, compared with that in adjacent nontumor tissues (Figure 8(a)). The fact that there is only one suitable binding site for hsa-miR-144-3p but two sites for hsa-miR-577 in each hsa_circRNA_0007334 molecule helps explain why the level of hsa-miR-577 downregulation is greater than the level of hsa-miR-144-3p downregulation. The expression levels of hsa-miR-144-3p or hsa-miR-577 were negatively correlated with hsa_circRNA_0007334 in PDAC tissue, as indicated by the two-tailed Pearson's correlation analysis (Figures 8(b) and 8(c)).

These results suggest that hsa_circRNA_0007334 plays a role by suppressing hsa-miR-144-3p and hsa-miR-577. Correspondingly, the level of COL1A1 upregulation is greater than the level of MMP7 upregulation. Taken together, these results strongly suggest that hsa_circRNA_0007334 plays a role in PDAC by functioning via the ceRNA mechanism by the competitive adsorption of hsa-miR-144-3p and hsa-miR-577 to enhance the level of MMP7 and COL1A1 expression.

### 3.9. Knockdown of hsa_circRNA_0007334 in the PANC-1 Cell Line

**Figure 9:**
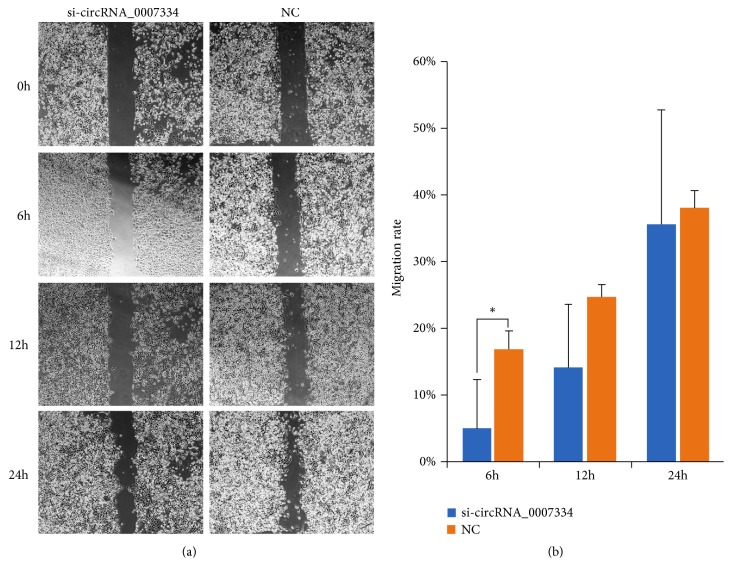
Wound healing assay after hsa_circRNA_0007334 knockdown in the PANC-1 cell line. (a) Microscopic cell imaging (6.4×) at 0, 6, 12, and 24 hours after hsa_circRNA_0007334 knockdown. (b) Migration rate based on scratch areas at 0, 6, 12, and 24 hours after hsa_circRNA_0007334 knockdown. *∗* indicates a significant difference compared to the NC group (p<0.05).

**Figure 10:**
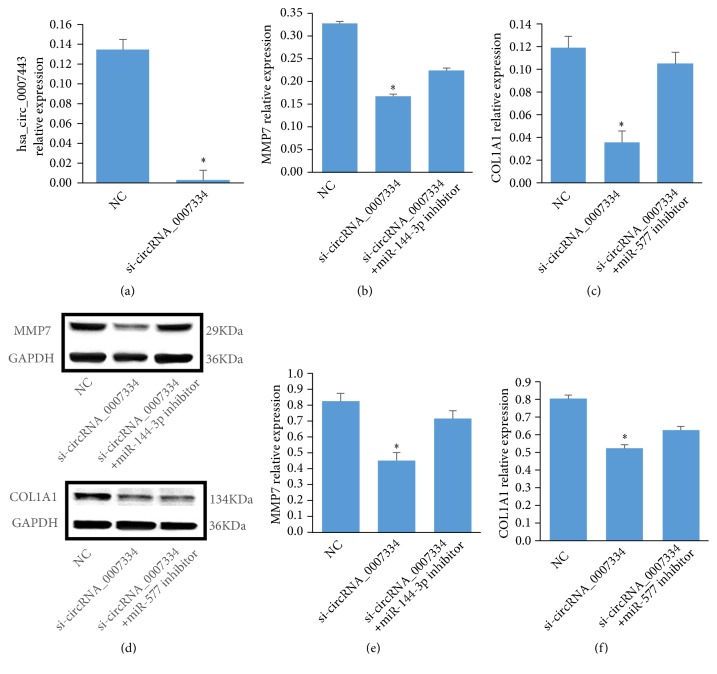
Expression profiles of hsa_circRNA_0007334, MMP7, and COL1A1 in the PANC-1 cell line after hsa_circRNA_0007334 knockdown. (a) The expression levels of hsa_circRNA_0007334 in the negative control (NC) and hsa_circRNA_0007334 knockdown groups. (b) The mRNA expression levels of MMP7 in the NC, hsa_circRNA_0007334 knockdown, and knockdown plus miR-144-3p inhibitor groups. (c) The mRNA expression levels of COL1A1 in the NC, hsa_circRNA_0007334 knockdown, and knockdown plus miR-577 inhibitor groups. (d) Western blot results of MMP7 and COL1A1 expression in the NC, hsa_circRNA_0007334 knockdown, and knockdown plus miRNA inhibitor groups. (e) The protein expression levels of MMP7 in the NC, hsa_circRNA_0007334 knockdown, and knockdown plus miR-144-3p inhibitor groups. (f) The protein expression levels of COL1A1 in the NC, hsa_circRNA_0007334 knockdown, and knockdown plus miR-577 inhibitor groups. *∗* indicates a significant difference compared to the NC group (p<0.05).

Furthermore, we used a siRNA to knockdown the expression level of circRNA_0007334 in the pancreatic cancer cell line PANC-1. Wound healing assay results showed that after siRNA knockdown, the migration ability of cells decreased significantly, especially at 6-12 hours after knockdown (Figure 9). RT-qPCR results showed that after siRNA knockdown, the expression level of hsa_circRNA_0007334 was significantly reduced (Figure 10(a)). The silencing efficiency of hsa_circRNA_0007334 was 98.04%. The expression levels of MMP7 and COL1A1 decreased significantly with the knockdown of hsa_circRNA_0007334. However, when the miR-144-3p inhibitor was added, the expression level of MMP7 was partially restored (Figure 10(b)), and the miR-577 inhibitor restored the expression level of COL1A1 (Figure 10(c)). Western blot analysis revealed the same results (Figures 10(d)–10(f)).

### 3.10. Survival Analysis of hsa-miR-144-3p, hsa-miR-577, MMP7, and COL1A1 Expression Levels in PDAC Patients

**Figure 11:**
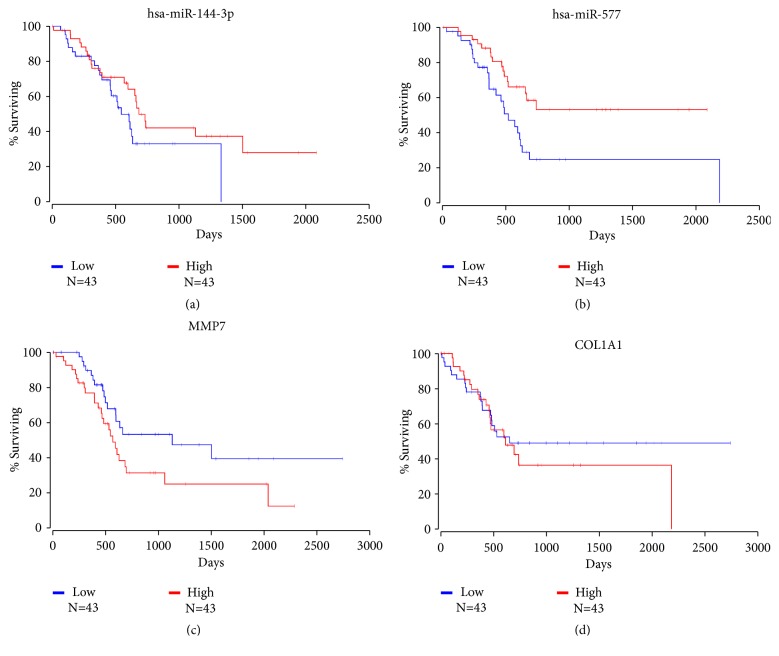
Survival analysis of hsa-miR-144-3p, hsa-miR-577, MMP7, and COL1A1 expression level in PDAC patients. The x-axis presents the survival time, and the y-axis presents the remaining rate of surviving patients. Red curves present high expression cases of each genes, and blue curves present low expression cases of each genes.

To further clarify the relationship between the expression levels of hsa-miR-144-3p, hsa-miR-577, MMP7, and COL1A1 in PDAC, we conducted a survey of the survival time of PDAC patients with different expression profiles. The survival data were obtained from the Cancer Genome Atlas (TCGA) database and analyzed by the OncoLnc database (http://www.oncolnc.org/). The top 25% of cases with high or low expression were selected for the survival analysis. As a result, 43 high expression cases and 43 low expression cases were included in the analysis. The results are presented as survival curves and are shown in Figure 11. As seen in the survival curves, the survival time of the low hsa-miR-144-3p and hsa-miR-577 expression group was significantly shorter than that of the high hsa-miR-144-3p and hsa-miR-577 expression group. Correspondingly, the survival time of the high MMP7 and COL1A1 expression group was significantly shorter than that of the low MMP7 and COL1A1 expression group. These results further confirm that hsa-miR-144-3p and hsa-miR-577 promote the development of PDAC through the regulation of MMP7 and COL1A1.

## 4. Discussion

As suggested in a considerable number of studies, the expression profiles of noncoding RNAs, including circRNAs, lncRNAs, and miRNAs, are abnormal in many types of cancer [17, 18]. Some of these proteins have been confirmed to play key roles in cancer pathogenesis [7, 19]. Although some studies have revealed that many circRNAs act as regulators in the development of PDAC [20, 21], the actual circRNAs involved and their functions remain unclear. According to previous studies, noncoding RNAs regulate gene expression via two main ways: cis-acting and transacting regulation. Cis-acting elements control the expression of genes that are positioned in the vicinity of the transcription sites and can sometimes spread the effect to long distances on the same chromosome, whereas transacting elements can either repress or activate gene expression at independent loci [5, 22, 23]. Based on cis-acting regulation, analyzing the parental gene from which it is derived to predict the potential functions of the circRNA is the most commonly used method. By analyzing the enrichment of the parental gene, we can determine which genes are affected in PDAC tissue and at the same time predict which circRNAs regulate these genes. Furthermore, we can predict the potential function of the circRNAs. GO and KEGG enrichment analyses were utilized to clarify the primary functions and pathways of the differentially expressed circRNAs based on the correlation between the circRNA and its parental gene. GO analysis revealed 217 parental genes that are closely related to the development of PDAC.

The ceRNA hypothesis illustrates the way in which different types of coding and noncoding members of the transcriptome interact with each other via miRNAs [7]. The members first compete for binding to miRNAs and then regulate mutual expression to build a complex posttranscriptional regulatory network [24]. We constructed a ceRNA network to predict the functions of the differentially expressed circRNAs. The ten circRNAs with the largest fold change (5 upregulated and 5 downregulated) and the ten circRNAs with the smallest p value were chosen to construct the network. The ceRNA network involves a total of 173 microRNAs and 1,460 target genes (Figure 3). Some circRNA ceRNA networks are very complicated; one circRNA can regulate a large number of target genes, or one target gene can be regulated by multiple circRNAs. In contrast, the ceRNA network of hsa_circRNA_0007334 is relatively simple. Moreover, the parental gene of hsa_circRNA_0007334 is associated with 23 of the 45 biological functions involved in the GO analysis, suggesting that hsa_circRNA_0007334 may be involved in numerous biological processes associated with PDAC. Therefore, we focused on the circRNA hsa_circRNA_0007334. The circRNA hsa_circRNA_0007334 has been mentioned in only a limited number of articles [25–28], and its functions in tumor development remain unknown. This study ranked its significance of the difference third among the 173 unregulated circRNAs.

Recent studies have shown that some mature circRNA molecules may consist of exon and intron sequences or that there are residual intron sequences. This kind of circRNA is called an EIciRNA [16]. Different from exonic circRNAs, which act as a miRNA sponge, and intronic circRNAs, which activate the transcription of maternal genes, EIciRNAs might hold factors through RNA-RNA interactions to further interact with the Pol II transcription complex at the promoters of parental genes to enhance gene expression. To determine whether there is an EIciRNA structure for hsa_circRNA_0007334, we performed reverse transcription PCR. The results suggest that the intron sequence is not detected in mature hsa_circRNA_0007334, which indicates that hsa_circRNA_0007334 is composed of exons only.

We have made efforts to understand the gene expression profiles between PDAC and normal tissues. First, the results of the three databases (HMDD, miRWalk 2.0, and MalaCards) were summarized to predict miRNAs, their target genes, and elements relevant to pancreatic cancer. Then, three mRNA microarray projects (GSE28735, GSE60980, and GSE62452) from GEO DataSets, with 163 PDAC tissues and 118 adjacent nontumor tissues in total, were investigated. The three gene expression profile microarray projects had several shared pathways that have been reported to be associated with the pathogenesis of PDAC. However, because of the differences in platforms and samples, the differentially expressed genes of the three projects showed differences as well. Next, a Venn analysis was conducted to identify the differentially expressed genes in both the databases and microarray projects. The results indicated that 17 genes were differentially expressed. Of all the 17 genes, MMP1, MMP7, MMP11, MMP14, COL1A1, COL1A2, FN1, LAMA3, NOX4, PLAU, CCL20, LEF1, and MET are highly expressed in PDAC tissues; BTG2, BNIP3, BACE1, and EGF are weakly expressed in adjacent nontumor tissues. As BACE1, CCL20, EGF, LEF1, and MET are not expressed in pancreatic tumors according to the NCBI-UniGene database (https://www.ncbi.nlm.nih.gov/unigene/); they were not included in the subsequent analyses.

On the basis of the PPI analysis of coexpression, colocalization, shared protein domains, and predicted or genetic interactions, we have learned more about the functions of and communication between these genes. MMP7 and COL1A1 are closely correlated with the occurrence and development of multiple tumors. MMP7 encodes a member of the peptidase M10 family of matrix metalloproteinases. Notably, proteins in this family play an active role in the breakdown of the extracellular matrix in normal physiological processes (such as embryonic development, reproduction, and tissue remodeling) and in disease processes (such as arthritis and metastasis). Additionally, MMP7 is highly expressed in multiple human malignant tumors [29–31] and is closely related to metastatic PDAC [32]. Therefore, it can be employed as a biomarker to risk-stratify PDAC patients [33, 34]. We also found that the expression of MMP7 in PDAC tumor cells shows a positive association with the presence of necrosis but a negative association with microvessel density [35]. In comparison, COL1A1 is a fibril-forming collagen in most connective tissues and is rich in the bone, cornea, dermis, and tendon. With these features, it is invariably considered a desmoplasia-related marker. In PDAC, COL1A1 can also be used as a biomarker to provide histologic presentations of human chronic pancreatitis transitioning to early tumorigenesis [36].

One hsa_circRNA_0007334-miRNA-target gene ceRNA network was established to research the potential interactions between the 13 genes and hsa_circRNA_0007334 (Figure 6). According to the results, MMP7 is targeted by hsa-miR-144-3p, while COL1A1 is targeted by hsa-miR-577. Moreover, the results of the ceRNA analysis were further substantiated by RT-qPCR in 10 PDAC tissues and 10 adjacent nontumor tissues. The miRNA hsa-miR-144 is closely correlated with multiple human malignant tumors [37–40], in that it induces cell cycle arrest and apoptosis in pancreatic cancer by targeting PRR11 [41]. The miRNA hsa-miR-577 also shows a strong correlation with multiple human malignant tumors [42–44], but its role in PDAC has not yet been documented. Generally, the “sponging theory” is more relevant to circRNAs with a very high number of miRNA binding sites. However, the regulation of gene expression is affected by many factors. When predicting the miRNAs that are most likely associated with the 20 most differentially expressed circRNAs, we found a maximum of only 3 binding sites for one miRNA on one circRNA among all 20 circRNAs. In this study, we found that hsa-miR-144-3p and hsa-miR-577 are significantly regulated by hsa_circRNA_0007334, which is one of the reasons we decided to further study these RNAs. Moreover, the ceRNA network of hsa_circRNA_0007334 is relatively simple, which may be because our choice of circRNA_0007334 has a simple ceRNA network and is less subject to other regulatory factors. In addition to our study, there are similar reports in other studies with fewer binding sites but significant regulatory effects [45–48].

A survey on the survival time of PDAC patients with different expression profiles was performed to further substantiate the relationship between the expression levels of hsa-miR-144-3p, hsa-miR-577, MMP7, and COL1A1 in PDAC. The survival time of the low hsa-miR-144-3p and hsa-miR-577 expression group was remarkably shorter than that of the high expression group, whereas the survival time of the high MMP7 and COL1A1 expression group was significantly shorter than that of the low expression group. However, we have to note the limitation of the current study: merely ten circRNAs with the largest fold change (five upregulated and five downregulated) and ten circRNAs with the smallest p value were chosen to construct the ceRNA network, but the roles of other circRNAs remain unclear.

## 5. Conclusions

In this study, we propose two regulatory pathways in PDAC: hsa_circRNA_0007334–hsa-miR-144-3p–MMP7 and hsa_circRNA_0007334–hsa-miR-577–COL1A1. The results indicate that by the competitive adsorption of hsa-miR-144-3p and hsa-miR-577, hsa_circRNA_0007334 functions as a ceRNA for MMP7 and COL1A1 to elevate their expression levels and plays an important role in PDAC.

## Figures and Tables

**Table 1:** Clinicopathological characteristics of patients with PDAC.

Clinicopathological characteristics	Patients in which RNA sequencing was performed (n=5)	Patients in which qPCR was performed (n=10)
Age (mean ± standard deviation)	59.67±8.26	60.70±8.45
Gender		
Male	2	6
Female	4	4
TNM stage^1^		
Stage 0	0	0
Stage IA	0	1
Stage IB	0	1
Stage II	1	4
Stage III	4	4
Stage IV	0	0

^1^ According to the WHO classification of tumors of the digestive system.

## Data Availability

The data used to support the findings of this study are included within the article.

## Supplementary Materials

Supplementary Materials Supplementary Data 1. The information about reads' mapping, quality filtering, normalization of data and analysis of differential expression. Supplementary Data 2. The information of differential expressed circRNAs. Supplementary Data 3. GO analysis on 217 parental genes of circRNAs. Supplementary Data 4. The predicted miRNAs binding to hsa_circRNA_0007334. Supplementary Figure 1. Identification of EIciRNA structure. (A) The pattern diagram of primer setting. (B) Agarose gel electrophoresis detection of reverse transcript PCR. F-R: amplification using F-primer and R-primer (expected size of product: 65 bp), F-R′: amplification using F-primer and R′-primer (expected size of product: 222 bp), F′-R: amplification using F′′-primer and R-primer (expected size of product: 218 bp), F′-R′: amplification using F′-primer and R′-primer (expected size of product: 375 bp).
