# Supplementary material for: Circular RNA hsa_circRNA_0007334 is Predicted to Promote MMP7 and COL1A1 Expression by Functioning as a miRNA Sponge in Pancreatic Ductal Adenocarcinoma
Source: J Oncol. 2019 Jul 24;2019:7630894. doi: 10.1155/2019/7630894 (PMC6681607; doi:10.1155/2019/7630894)
Supplement: Supplementary Materials — Supplementary Data 1. The information about reads' mapping, quality filtering, normalization of data and analysis of differential expression. Supplementary Data 2. The information of differential expressed circRNAs. Supplementary Data 3. GO analysis on 217 parental genes of circRNAs. Supplementary Data 4. The predicted miRNAs binding to hsa_circRNA_0007334. Supplementary Figure 1. Identification of EIciRNA structure. (A) The pattern diagram of primer setting. (B) Agarose gel electrophoresis detection of reverse transcript PCR. F-R: amplification using F-primer and R-primer (expected size of product: 65 bp), F-R′: amplification using F-primer and R′-primer (expected size of product: 222 bp), F′-R: amplification using F′′-primer and R-primer (expected size of product: 218 bp), F′-R′: amplification using F′-primer and R′-primer (expected size of product: 375 bp). [file 7630894.f1.pdf]

Circular RNA hsa_circRNA_0007334 is Predicted to Promote MMP7 and COL1A1 Expression by Functioning as miRNA Sponge in Pancreatic Ductal Adenocarcinoma

Jinghui Yang[1], Xianling Cong[3], Ming Ren[4], Hongyan Sun[3], Tao Liu [5], Gaoyang Chen[4], Qingyu Wang[4], Zhaoyan Li[4], Shan Yu[2*], Qiwei Yang[4,6*]

Reads' Mapping

Sample name	T1	T2	T3	T4	T5	N1	N2	N3	N4	N5
Total reads	65459710	73486532	67158268	55900776	62333044	55590510	83180310	68719040	67928714	55385732
Total mapped	59316900 (90.62%)	66826568 (90.94%)	57373700 (85.43%)	49960555 (89.37%)	54491826 (87.42%)	51489537 (92.62%)	76606797 (92.1%)	59151794 (86.08%)	61192081 (90.08%)	51014081 (92.11%)
Multiple mapped	5607957 (8.57%)	5647470 (7.69%)	5348820 (7.96%)	5194563 (9.29%)	5164715 (8.29%)	4855697 (8.73%)	7184264 (8.64%)	7128627 (10.37%)	8821875 (12.99%)	4139852 (7.47%)
Uniquely mapped	53708943 (82.05%)	61179098 (83.25%)	52024880 (77.47%)	44765992 (80.08%)	49327111 (79.13%)	46633840 (83.89%)	69422533 (83.46%)	52023167 (75.7%)	52370206 (77.1%)	46874229 (84.63%)
Read-1	27582550 (42.14%)	31344483 (42.65%)	26806585 (39.92%)	22911223 (40.99%)	25416915 (40.78%)	23861332 (42.92%)	35601818 (42.8%)	27003106 (39.29%)	26796743 (39.45%)	24084039 (43.48%)
Read-2	26126393 (39.91%)	29834615 (40.6%)	25218295 (37.55%)	21854769 (39.1%)	23910196 (38.36%)	22772508 (40.96%)	33820715 (40.66%)	25020061 (36.41%)	25573463 (37.65%)	22790190 (41.15%)
Reads map to '+'	27049064 (41.32%)	30931750 (42.09%)	26239351 (39.07%)	22578850 (40.39%)	24929924 (39.99%)	23561941 (42.38%)	35099420 (42.2%)	26505766 (38.57%)	26407159 (38.87%)	23732793 (42.85%)
Reads map to '-'	26659879 (40.73%)	30247348 (41.16%)	25785529 (38.4%)	22187142 (39.69%)	24397187 (39.14%)	23071899 (41.5%)	34323113 (41.26%)	25517401 (37.13%)	25963047 (38.22%)	23141436 (41.78%)
Non-splice reads	51892094 (79.27%)	59148927 (80.49%)	49325975 (73.45%)	43065778 (77.04%)	47297714 (75.88%)	45862802 (82.5%)	68279190 (82.09%)	50594745 (73.63%)	51632914 (76.01%)	45763995 (82.63%)
Splice reads	1816849 (2.78%)	2030171 (2.76%)	2698905 (4.02%)	1700214 (3.04%)	2029397 (3.26%)	771038 (1.39%)	1143343 (1.37%)	1428422 (2.08%)	737292 (1.09%)	1110234 (2%)
Reads mapped in proper pairs	51272680 (78.33%)	58551796 (79.68%)	49419838 (73.59%)	42988366 (76.9%)	46976936 (75.36%)	44449828 (79.96%)	65375240 (78.59%)	49423140 (71.92%)	50409928 (74.21%)	44250084 (79.89%)
Proper-paired reads map to different chrom	2 (0%)	0 (0%)	2 (0%)	8 (0%)	2 (0%)	0 (0%)	0 (0%)	0 (0%)	0 (0%)	2 (0%)

Quality Filtering

Sample name	T1	T2	T3	T4	T5	N1	N2	N3	N4	N5
Clean Reads	32729855 (93.37%)	36743266 (93.52%)	33579134 (86.26%)	27950388 (89.19%)	31166522 (85.76%)	27795255 (95.14%)	41590155 (96.12%)	34359520 (87.72%)	33964357 (87.70%)	27692866 (96.03%)
Containing N	403960 (1.15%)	456449 (1.16%)	598427 (1.54%)	414751 (1.32%)	228050 (0.63%)	512183 (1.75%)	477672 (1.10%)	398934 (1.02%)	266829 (0.69%)	502797 (1.74%)
Low Quality	0.1 (0.00%)	0.1 (0.00%)	0.1 (0.00%)	0.1 (0.00%)	0.1 (0.00%)	0.1 (0.00%)	0.1 (0.00%)	0.1 (0.00%)	0.1 (0.00%)	0.1 (0.00%)
Adapter Related	1921264 (5.48%)	2090157 (5.32%)	4752140 (12.21%)	2973079 (9.49%)	4948847 (13.62%)	907721 (3.11%)	1201001 (2.78%)	4411429 (11.26%)	4497436 (11.61%)	642428 (2.23%)

Normalization of Data

$$\text{Normalization value} = \frac{\text{Read Count} * 1{,}000{,}000}{\sum \text{circRNA readcount}}$$

Circular RNA hsa_circRNA_0007334 is Predicted to Promote MMP7 and COL1A1 Expression by Functioning as miRNA Sponge in Pancreatic Ductal Adenocarcinoma

Jinghui Yang[1], Xianling Cong[3], Ming Ren[4], Hongyan Sun[3], Tao Liu [5], Gaoyang Chen[4], Qingyu Wang[4], Zhaoyan Li[4], Shan Yu[2*], Qiwei Yang[4,6*]

Annotations		Fold change and Regulation		P-value and P-adj	
circRNA ID	GeneSymbol	FC (abs)	Regulation	P-val	P-adj
hsa_circ_0056856	ITGB6	8.0341	up	2.91E-07	5.13E-05
hsa_circ_0049792	EMR2	6.7007	up	2.02E-05	0.0014822
hsa_circ_0000692	ITGAM	6.6262	up	2.65E-05	0.0017553
hsa_circ_0008599	MIR31HG	6.6075	up	0.00015567	0.0064513
hsa_circ_0005320	SEPT9	6.575	up	3.32E-05	0.0020075
hsa_circ_0075829	LINC00340	6.3798	up	7.06E-05	0.0035536
hsa_circ_0001277	SLC4A7	6.3215	up	7.66E-05	0.003766
hg38_circ_0007301		6.2788	up	0.0001115	0.005014
hsa_circ_0081343	ARPC1B	6.1535	up	0.00014957	0.0064181
hsa_circ_0005311	TRIO	6.1257	up	0.00016683	0.0067806
hg38_circ_0004289		6.1225	up	0.00015183	0.0064181
hsa_circ_0006973	PDZRN3	6.1045	up	0.00016489	0.0067669
hsa_circ_0004032	ATXN1	6.104	up	0.00020913	0.0074284
hsa_circ_0004300	ANP32E	6.064	up	0.00018224	0.0071438
hg38_circ_0015943		6.0525	up	0.00019169	0.0071877
hsa_circ_0000682	PRKCB	6.0458	up	0.00019974	0.0072163
hsa_circ_0002882	AXL	6.0266	up	0.00026468	0.0086728
hsa_circ_0005085	ASAP2	5.9524	up	0.00037515	0.010546
hsa_circ_0052099	ZNF880	5.9336	up	0.00026052	0.0086728
hsa_circ_0018087	ZEB1	5.9101	up	0.00028173	0.0089538
hg38_circ_0007606		5.894	up	0.00029697	0.0091432
hg38_circ_0008704		5.8622	up	0.00036658	0.010469
hsa_circ_0005734	FAM53B	5.8221	up	0.000408	0.011199
hg38_circ_0007186		5.7719	up	0.00064168	0.016049
hg38_circ_0007297		5.7302	up	0.00053996	0.013917
hsa_circ_0039158	ITGAM	5.6933	up	0.00060968	0.01534
hsa_circ_0008063	UBE2J2	5.6828	up	0.0005888	0.014924
hg38_circ_0014522		5.6578	up	0.00082033	0.019481
hsa_circ_0017636	SFMBT2	5.6342	up	0.00066586	0.016556
hsa_circ_0089974	NHS	5.6168	up	0.00070478	0.01722
hg38_circ_0018384		5.5107	up	0.00095499	0.021944
hsa_circ_0036988	CHD2	5.4987	up	0.0013177	0.026398
hg38_circ_0014949		5.4983	up	0.0012711	0.02621
hsa_circ_0006482	CBFB	5.4939	up	0.00098432	0.02249
hg38_circ_0020621		5.4756	up	0.0012439	0.02603
hsa_circ_0023988	NOX4	5.4639	up	0.0010605	0.023593
hsa_circ_0004142	ALKBH5	5.4555	up	0.0011053	0.024335
hsa_circ_0005211	MYO1E	5.4518	up	0.001305	0.026398
hsa_circ_0031569	HEATR5A	5.4411	up	0.0013079	0.026398
hsa_circ_0064460	NR2C2	5.4291	up	0.0011454	0.025086
hsa_circ_0002015	MMD	5.4217	up	0.0011744	0.02542
hg38_circ_0008591		5.3445	up	0.0014584	0.028022
hg38_circ_0011267		5.3314	up	0.0015713	0.029069
hsa_circ_0003271	AXL	5.3302	up	0.00157	0.029069
hsa_circ_0006051	LRP5L	5.3302	up	0.0016402	0.029757
hsa_circ_0001240	NFAM1	5.3294	up	0.0016885	0.030243
hsa_circ_0003563	RUNX2	5.3137	up	0.0018751	0.032219
hsa_circ_0032689	FLVCR2	5.3126	up	0.0015562	0.029069
hsa_circ_0057867	NBEAL1	5.3106	up	0.0015596	0.029069
hsa_circ_0002111	PSD3	5.2822	up	0.0017109	0.030259

hsa_circ_0023555	C2CD3	5.2793	up	0.0020778	0.034716
hsa_circ_0008537	GLI3	5.2649	up	0.0019365	0.033007
hsa_circ_0002247	LTBP1	5.2602	up	0.0018394	0.031866
hg38_circ_0023450		5.2574	up	0.0021962	0.035568
hsa_circ_0006708	IRAK3	5.2457	up	0.0023183	0.036567
hsa_circ_0008223	XPO6	5.241	up	0.0019294	0.033007
hsa_circ_0087232	PCSK5	5.2046	up	0.0021174	0.034732
hsa_circ_0008432	TPM4	5.1952	up	0.0028869	0.042321
hsa_circ_0062649	ADRBK2	5.1881	up	0.0022868	0.03634
hsa_circ_0005687	CRISPLD2	5.1879	up	0.0021409	0.03494
hsa_circ_0006358	PALLD	5.1788	up	0.0023184	0.036567
hsa_circ_0000906	TMEM38A	5.1771	up	0.0027281	0.041038
hsa_circ_0051123	LTBP4	5.1632	up	0.0026832	0.040653
hsa_circ_0006569	PTPN9	5.1423	up	0.0025918	0.039774
hsa_circ_0004365	SEMA3C	5.1243	up	0.0026041	0.039774
hsa_circ_0007733	DOCK11	5.1169	up	0.0030168	0.043671
hsa_circ_0049487	CNN1	5.1139	up	0.0028842	0.042321
hsa_circ_0001252	TBC1D22A	5.1093	up	0.0031691	0.044872
hsa_circ_0079668	TAX1BP1	5.1076	up	0.0027092	0.040899
hsa_circ_0073396	MCTP1	5.101	up	0.0031741	0.044872
hg38_circ_0027589		5.0908	up	0.0028174	0.041939
hg38_circ_0010763		5.0865	up	0.0028177	0.041939
hsa_circ_0007843	ARHGAP32	5.0798	up	0.0028345	0.04204
hg38_circ_0021801		5.0789	up	0.0035142	0.047307
hsa_circ_0003449	MEIS3	5.0778	up	0.002926	0.04265
hsa_circ_0056664	GTDC1	5.0605	up	0.0030657	0.04379
hg38_circ_0002385		5.0493	up	0.003514	0.047307
hsa_circ_0001115	DGKD	5.0459	up	0.0030588	0.04379
hsa_circ_0058773	DGKD	5.0444	up	0.0036625	0.048531
hsa_circ_0032457	PCNX	5.0429	up	0.0037744	0.049394
hg38_circ_0022002		5.0423	up	0.0033326	0.046338
hsa_circ_0069052	EVC2	4.9843	up	0.0037556	0.049301
hsa_circ_0000450	RHOF	4.6007	up	3.52E-08	9.91E-06
hsa_circ_0004968	DCBLD2	4.5611	up	8.94E-05	0.0042453
hsa_circ_0007210	TCONS_l2_0001[illegible]	4.5393	up	7.45E-06	0.00066336
hsa_circ_0003380	ZFPM2	4.449	up	1.17E-07	2.75E-05
hsa_circ_0000939	APOC1	4.394	up	1.86E-07	3.58E-05
hsa_circ_0075828	LINC00340	4.2866	up	1.99E-09	1.40E-06
hg38_circ_0007554		4.2575	up	3.52E-06	0.00040135
hsa_circ_0007334	MBOAT2	4.1784	up	3.41E-09	2.06E-06
hsa_circ_0007904	HDAC9	4.1741	up	0.0001834	0.0071438
hsa_circ_0004650	None	4.1054	up	9.72E-06	0.00080543
hsa_circ_0006836	CLEC16A	4.0974	up	0.0002963	0.0091432
hsa_circ_0009142	CAP1	4.0947	up	4.29E-05	0.002485
hsa_circ_0000431	DRAM1	4.0869	up	0.00011584	0.0051541
hsa_circ_0002873	CREBL2	4.0841	up	0.00036902	0.010469
hg38_circ_0025574		4.0693	up	6.61E-06	0.00062046
hsa_circ_0002360	RUNX1	4.0054	up	6.71E-05	0.0034412
hsa_circ_0005019	CHSY1	3.9988	up	1.62E-07	3.43E-05
hsa_circ_0004092	MB21D1	3.9078	up	1.50E-05	0.0011285
hg38_circ_0000996		3.884	up	0.00026312	0.0086728
hsa_circ_0007439	PPP1CB	3.858	up	0.00039239	0.010912
hsa_circ_0008844	MFSD2A	3.8047	up	0.0017819	0.031254
hsa_circ_0039161	ITGAX	3.7469	up	0.00036861	0.010469
hsa_circ_0052877	C2orf43	3.7184	up	0.0014663	0.028046
hsa_circ_0003267	ME1	3.7141	up	0.00073087	0.017553
hsa_circ_0008264	NAV1	3.6842	up	0.0018159	0.031719
hsa_circ_0033144	BCL11B	3.6012	up	7.04E-06	0.00064724

hg38_circ_0004316		3.5919	up	0.00019348	0.0071877
hsa_circ_0005317	TRPM4	3.5351	up	0.0027491	0.041208
hsa_circ_0004891	CNN2	3.5342	up	1.23E-08	4.01E-06
hsa_circ_0002194	RELL1	3.4956	up	0.00032853	0.0096436
hsa_circ_0009125	HABP4	3.4824	up	0.0035981	0.047979
hsa_circ_0000230	ZEB1	3.4704	up	2.77E-05	0.0017553
hsa_circ_0069152	AFAP1	3.3702	up	1.81E-07	3.58E-05
hsa_circ_0000707	CBFB	3.3602	up	0.00019324	0.0071877
hsa_circ_0005620	SH3PXD2A	3.3422	up	1.44E-06	0.00019015
hsa_circ_0031113	TEP1	3.3401	up	0.0022732	0.036328
hsa_circ_0008590	RELB	3.3269	up	3.61E-06	0.00040135
hsa_circ_0005230	DNM3OS	3.2707	up	4.51E-05	0.0025445
hsa_circ_0007940	ARPC1B	3.2571	up	0.0020962	0.034732
hsa_circ_0000972	MBOAT2	3.2064	up	4.55E-08	1.20E-05
hsa_circ_0005898	MYOF	3.1994	up	0.00019385	0.0071877
hsa_circ_0006692	TCONS_0002553	3.1955	up	0.00013532	0.005897
hsa_circ_0002075	BMP1	3.1686	up	4.70E-05	0.002614
hg38_circ_0004299		3.1187	up	9.59E-07	0.00013517
hsa_circ_0005358	GLI1	3.0609	up	0.0012142	0.025703
hsa_circ_0039076	SRCAP	3.054	up	0.0015032	0.028621
hg38_circ_0004278		3.0414	up	0.00026893	0.0086777
hsa_circ_0006608	PFKP	2.9926	up	0.0012141	0.025703
hsa_circ_0002837	TGFB1	2.9874	up	0.00023059	0.0079264
hsa_circ_0007731	MYO9B	2.9729	up	0.0010908	0.02414
hsa_circ_0008615	PPP1R13L	2.9134	up	0.00044618	0.011862
hsa_circ_0002130	C3	2.9109	up	0.00032489	0.0096412
hsa_circ_0087938	C9orf5	2.8982	up	0.0034934	0.047307
hsa_circ_0003071	SPATS2L	2.8619	up	3.36E-06	0.0003945
hsa_circ_0005571	IFI30	2.854	up	0.0003957	0.010932
hsa_circ_0005175	HPS4	2.8407	up	3.71E-05	0.0022072
hsa_circ_0006877	LDLR	2.8375	up	0.00010733	0.0049312
hsa_circ_0004954	PITPNA	2.7625	up	0.0022145	0.035727
hg38_circ_0007562		2.7533	up	0.001016	0.02273
hsa_circ_0004228	EVC2	2.7471	up	6.76E-05	0.0034412
hsa_circ_0000375	IFFO1	2.7356	up	5.70E-06	0.00057134
hsa_circ_0050334	URI1	2.7105	up	0.0028584	0.042246
hsa_circ_0000732	SCARF1	2.6791	up	1.26E-05	0.00096762
hsa_circ_0004458	PSD3	2.6614	up	0.0035685	0.047861
hsa_circ_0051680	DHX34	2.6396	up	7.53E-06	0.00066336
hsa_circ_0002532	TCONS_0001718	2.6382	up	0.00029655	0.0091432
hsa_circ_0002878	FBLN1	2.627	up	0.0026064	0.039774
hsa_circ_0008833	SAMD3	2.6131	up	0.00033541	0.0097779
hsa_circ_0000130	SNX27	2.6053	up	0.0016121	0.0295
hsa_circ_0001821	TCONS_0001535	2.6009	up	0.00024981	0.0084474
hsa_circ_0001721	CDK14	2.5473	up	4.96E-05	0.0026878
hsa_circ_0000437	CORO1C	2.4894	up	2.78E-13	5.88E-10
hsa_circ_0008521	PSEN1	2.4315	up	1.02E-08	3.94E-06
hg38_circ_0014462		2.4156	up	0.0002985	0.0091432
hsa_circ_0000690	ITGAL	2.3983	up	7.22E-07	0.00010898
hsa_circ_0008732	BNC2	2.3945	up	5.87E-07	9.92E-05
hsa_circ_0000228	ZEB1	2.385	up	7.39E-05	0.0036732
hsa_circ_0000688	MVP	2.3754	up	1.02E-08	3.94E-06
hsa_circ_0000446	TAOK3	2.3735	up	0.0010071	0.02273
hsa_circ_0008934	ASAP1	2.3226	up	0.00018591	0.0071438
hsa_circ_0074371	ARHGAP26	2.2921	up	2.70E-06	0.00033505
hg38_circ_0014786		2.2702	up	0.0002685	0.0086777
hsa_circ_0008016	FGFR1	2.235	up	2.78E-05	0.0017553
hsa_circ_0004476	ASXL2	2.2151	up	0.0020986	0.034732

hsa_circ_0003848	PSEN1	2.193	up	4.44E-05	0.0025365
hsa_circ_0000362	CBL	2.1725	up	0.0014264	0.027786
hsa_circ_0008194	AHNAK	2.1568	up	0.0017061	0.030259
hsa_circ_0000211	SFMBT2	2.1235	up	0.00026371	0.0086728
hg38_circ_0005277		2.1152	up	0.0031918	0.044927
hsa_circ_0085616	ASAP1	2.0958	up	2.71E-05	0.0017553
hsa_circ_0000754	SSH2	2.0734	up	0.0022775	0.036328
hsa_circ_0027440	TBC1D30	-2.1423	down	0.00019763	0.0072015
hsa_circ_0048965	INSR	-2.2209	down	0.00027598	0.0088375
hsa_circ_0001766	PDIA4	-2.2511	down	2.70E-05	0.0017553
hsa_circ_0001786	DCTN6	-2.2654	down	0.0032958	0.045978
hsa_circ_0021516	GAS2	-2.3044	down	0.00047697	0.012601
hg38_circ_0019004		-2.3339	down	0.0013939	0.027405
hsa_circ_0002472	PLXNA2	-2.3696	down	0.00084772	0.020019
hsa_circ_0005204	SLC43A1	-2.4017	down	2.83E-10	3.59E-07
hsa_circ_0044646	SPAG9	-2.4098	down	0.00022351	0.0078082
hsa_circ_0004913	TEX2	-2.413	down	0.00053024	0.013818
hg38_circ_0000339		-2.4443	down	0.00010664	0.0049312
hsa_circ_0002562	TCONS_0001474	-2.4459	down	0.00072939	0.017553
hsa_circ_0070440	FAM13A	-2.6008	down	0.00015406	0.0064478
hg38_circ_0005953		-2.6194	down	3.40E-10	3.59E-07
hsa_circ_0001566	MAPK9	-2.6608	down	7.78E-06	0.00067149
hsa_circ_0004844	ARSE	-2.6632	down	0.00042137	0.011345
hsa_circ_0040188	AARS	-2.7272	down	0.0001739	0.0069348
hsa_circ_0069922	SLC4A4	-2.8131	down	0.0013153	0.026398
hg38_circ_0019734		-2.8233	down	0.00092373	0.021454
hsa_circ_0004587	TMEM181	-2.8674	down	0.0015176	0.028705
hg38_circ_0026403		-2.8829	down	0.0015212	0.028705
hg38_circ_0022683		-2.8983	down	0.00041651	0.011286
hsa_circ_0004238	PTK2	-2.9896	down	0.00095523	0.021944
hsa_circ_0007513	GCAT	-3.0543	down	1.08E-07	2.68E-05
hsa_circ_0006374	ARCN1	-3.0637	down	0.00058962	0.014924
hsa_circ_0008676	SLC4A4	-3.1403	down	0.0021821	0.035477
hsa_circ_0009133	MFSD8	-3.1515	down	0.0016033	0.029466
hg38_circ_0024874		-3.1755	down	3.07E-05	0.0019058
hsa_circ_0005115	SEC63	-3.4096	down	0.0033506	0.046436
hg38_circ_0024699		-3.4752	down	1.14E-08	4.01E-06
hsa_circ_0015816	NR5A2	-3.6649	down	0.00011019	0.0050083
hg38_circ_0018658		-3.6859	down	0.00041637	0.011286
hsa_circ_0005497	CNKSR3	-3.7455	down	0.0014518	0.028022
hg38_circ_0018751		-3.7789	down	0.00067088	0.016584
hsa_circ_0035435	CGNL1	-3.7974	down	1.62E-07	3.43E-05
hsa_circ_0004627	UTP20	-3.8239	down	0.0030364	0.04379
hg38_circ_0001795		-3.8782	down	0.0030664	0.04379
hg38_circ_0027570		-3.8944	down	0.000312	0.0094203
hsa_circ_0006479	TEX2	-3.9767	down	0.0011787	0.02542
hg38_circ_0021274		-4.0077	down	0.00142	0.027786
hg38_circ_0023742		-4.0264	down	0.00043405	0.011612
hg38_circ_0005777		-4.0554	down	0.0018681	0.032219
hsa_circ_0080209	GRB10	-4.1053	down	5.51E-05	0.002911
hsa_circ_0002059	MAN1A2	-4.1367	down	1.16E-05	0.00092444
hg38_circ_0000214		-4.1688	down	5.80E-05	0.0030253
hg38_circ_0000241		-4.25	down	0.003122	0.044433
hg38_circ_0004194		-4.296	down	6.50E-07	0.00010169
hsa_circ_0002383	STARD10	-4.3627	down	0.0010163	0.02273
hsa_circ_0002979	CEACAM16	-4.379	down	8.12E-07	0.00011842
hg38_circ_0027050		-4.4012	down	0.0026027	0.039774
hg38_circ_0012894		-4.5194	down	0.00072224	0.017545

hg38_circ_0011914		-4.5464	down	0.0036387	0.048368
hg38_circ_0012792		-4.5575	down	0.0033754	0.046627
hg38_circ_0024497		-4.5761	down	5.23E-05	0.0028006
hg38_circ_0022684		-4.6932	down	1.01E-06	0.0001377
hsa_circ_0026920	SLC39A5	-4.7203	down	2.30E-05	0.0016305
hg38_circ_0011449		-4.8835	down	6.71E-09	3.54E-06
hg38_circ_0024330		-4.989	down	2.69E-06	0.00033505
hg38_circ_0011908		-5.0002	down	0.0022312	0.03586
hg38_circ_0012788		-5.0077	down	5.97E-06	0.00057369
hg38_circ_0027060		-5.0813	down	0.00017149	0.0069035
hg38_circ_0015287		-5.1143	down	2.03E-05	0.0014822
hg38_circ_0023664		-5.2185	down	0.0037003	0.048879
hg38_circ_0011912		-5.226	down	3.26E-06	0.00039421
hsa_circ_0008804	KDM3A	-5.2566	down	0.0034482	0.047018
hg38_circ_0002864		-5.2996	down	0.0034284	0.046899
hsa_circ_0002891	PDIA3	-5.3091	down	0.0030051	0.043651
hsa_circ_0001325	BBX	-5.3218	down	0.0032098	0.044927
hsa_circ_0047473	KIAA1328	-5.3243	down	0.0032086	0.044927
hsa_circ_0041154	RPH3AL	-5.3252	down	4.47E-06	0.00048457
hsa_circ_0058404	OBSL1	-5.41	down	0.0026189	0.03982
hg38_circ_0028350		-5.4386	down	0.0024977	0.038674
hg38_circ_0000232		-5.4524	down	0.0024535	0.038411
hg38_circ_0012787		-5.4956	down	0.0021199	0.034732
hg38_circ_0011904		-5.5215	down	0.0020047	0.033761
hg38_circ_0022333		-5.5298	down	0.0017293	0.030458
hsa_circ_0007123	MLL3	-5.537	down	0.0019786	0.033454
hsa_circ_0068389	MAP3K13	-5.5613	down	0.001635	0.029757
hsa_circ_0021346	SOX6	-5.5901	down	0.0017005	0.030259
hsa_circ_0006290	SLC7A1	-5.6326	down	0.0013412	0.026616
hg38_circ_0020070		-5.6646	down	0.0012517	0.026063
hsa_circ_0079223	SDK1	-5.6768	down	0.0011742	0.02542
hg38_circ_0000208		-5.6818	down	0.0012247	0.025755
hg38_circ_0011913		-5.6985	down	1.71E-09	1.40E-06
hg38_circ_0004887		-5.6998	down	0.0012922	0.026398
hg38_circ_0012456		-5.7978	down	0.00085543	0.020088
hg38_circ_0014361		-5.8793	down	0.00068824	0.016914
hg38_circ_0003245		-5.98	down	0.00051735	0.013583
hg38_circ_0024489		-6.0315	down	0.0020601	0.034556
hg38_circ_0024500		-6.0528	down	0.0019611	0.033291
hg38_circ_0027057		-6.1062	down	0.00035879	0.010388
hg38_circ_0011900		-6.132	down	6.12E-15	2.59E-11
hg38_circ_0015970		-6.1395	down	0.00037673	0.010546
hg38_circ_0011910		-6.1466	down	0.00032276	0.0096412
hg38_circ_0000217		-6.3069	down	0.00020568	0.0073678
hg38_circ_0023830		-6.3733	down	0.00015155	0.0064181
hg38_circ_0011906		-6.7319	down	4.90E-05	0.0026878
hg38_circ_0020077		-6.8856	down	2.45E-05	0.0016883
hsa_circ_0004390	LPAR3	-7.1784	down	9.34E-06	0.0007895
hg38_circ_0011903		-7.3064	down	4.59E-06	0.00048503
hg38_circ_0011450		-7.3323	down	4.71E-06	0.00048516
hg38_circ_0012893		-7.8084	down	6.39E-07	0.00010169
hg38_circ_0011453		-8.1851	down	2.20E-07	4.04E-05
hg38_circ_0011454		-8.567	down	9.72E-09	3.94E-06
hg38_circ_0012785		-8.8801	down	2.98E-08	8.99E-06

Circular RNA hsa_circRNA_0007334 is Predicted to Promote MMP7 and COL1A1 Expression by Functioning as miRNA Sponge in Pancreatic Ductal Adenocarcinoma

Jinghui Yang[1], Xianling Cong[3], Ming Ren[4], Hongyan Sun[3], Tao Liu [5], Gaoyang Chen[4], Qingyu Wang[4], Zhaoyan Li[4], Shan Yu[2*], Qiwei Yang[4,6*]

GO analysis

Term_type	Description	CAD_item	%		GO_accession	Gene
molecular_function	enzyme binding	34	15.66820276		GO:0019899	ENSG00000166501,ENSG00000129566,ENSG00000079841,ENSG00000197442,ENSG00000054611,ENSG00000110925,ENSG00000131236,ENSG00000038382,ENSG00000169180,ENSG00000116514,ENSG00000111490,ENSG00000140575,ENSG00000073803,ENSG00000124164,ENSG00000124126,ENSG00000164292,ENSG00000048052,ENSG00000169398,ENSG00000099282,ENSG00000213639,ENSG00000110395,ENSG00000124571,ENSG00000110880,ENSG00000148498,ENSG00000175899,ENSG00000147251,ENSG00000180530,ENSG00000013364,ENSG00000105329,ENSG00000106571,ENSG00000160087,ENSG00000008294,ENSG00000181031,ENSG00000110075
molecular_function	hydrolase activity	34	15.66820276		GO:0016787	ENSG00000090861,ENSG00000173575,ENSG00000215704,ENSG00000175535,ENSG00000184640,ENSG00000142789,ENSG00000175497,ENSG00000142615,ENSG00000128849,ENSG00000134815,ENSG00000243480,ENSG00000213639,ENSG00000157483,ENSG00000118322,ENSG00000048052,ENSG00000141298,ENSG00000164292,ENSG00000080603,ENSG00000140470,ENSG00000168487,ENSG00000187021,ENSG00000240038,ENSG00000072657,ENSG00000133392,ENSG00000118961,ENSG00000153002,ENSG00000099139,ENSG00000219073,ENSG00000198162,ENSG00000157399,ENSG00000091704,ENSG00000266200,ENSG00000197102,ENSG00000167004
molecular_function	nucleotide binding	38	17.51152074		GO:0000166	ENSG00000116350,ENSG00000130529,ENSG00000135090,ENSG00000173575,ENSG00000090861,ENSG00000160087,ENSG00000184640,ENSG00000058091,ENSG00000080603,ENSG00000159216,ENSG00000067057,ENSG00000090376,ENSG00000118322,ENSG00000164292,ENSG00000169398,ENSG00000066468,ENSG00000157483,ENSG00000077044,ENSG00000134815,ENSG00000167601,ENSG00000171105,ENSG00000100077,ENSG00000164430,ENSG00000086991,ENSG00000073803,ENSG00000154310,ENSG00000077782,ENSG00000038382,ENSG00000133392,ENSG00000154930,ENSG00000197442,ENSG00000197102,ENSG00000124813,ENSG00000159692,ENSG00000166501,ENSG00000119314,ENSG00000106049,ENSG00000129566
molecular_function	nucleoside phosphate binding	38	17.51152074		GO:1901265	ENSG00000129566,ENSG00000166501,ENSG00000119314,ENSG00000106049,ENSG00000124813,ENSG00000159692,ENSG00000154930,ENSG00000197442,ENSG00000197102,ENSG00000077782,ENSG00000038382,ENSG00000133392,ENSG00000073803,ENSG00000154310,ENSG00000171105,ENSG00000100077,ENSG00000164430,ENSG00000086991,ENSG00000157483,ENSG00000077044,ENSG00000134815,ENSG00000167601,ENSG00000090376,ENSG00000118322,ENSG00000169398,ENSG00000164292,ENSG00000066468,ENSG00000080603,ENSG00000159216,ENSG00000067057,ENSG00000173575,ENSG00000090861,ENSG00000160087,ENSG00000058091,ENSG00000184640,ENSG00000130529,ENSG00000135090,ENSG00000116350
molecular_function	small molecule binding	39	17.97235023		GO:0036094	ENSG00000169398,ENSG00000066468,ENSG00000164292,ENSG00000090376,ENSG00000130208,ENSG00000118322,ENSG00000167601,ENSG00000157483,ENSG00000077044,ENSG00000134815,ENSG00000067057,ENSG00000080603,ENSG00000159216,ENSG00000135090,ENSG00000130529,ENSG00000160087,ENSG00000184640,ENSG00000058091,ENSG00000173575,ENSG00000090861,ENSG00000116350,ENSG00000106049,ENSG00000166501,ENSG00000119314,ENSG00000129566,ENSG00000154930,ENSG00000197442,ENSG00000197102,ENSG00000159692,ENSG00000124813,ENSG00000038382,ENSG00000133392,ENSG00000077782,ENSG00000086991,ENSG00000100077,ENSG00000171105,ENSG00000164430,ENSG00000154310,ENSG00000073803
molecular_function	metal ion binding	53	24.42396313		GO:0046872	ENSG00000072657,ENSG00000177463,ENSG00000167460,ENSG00000005844,ENSG00000148516,ENSG00000116514,ENSG00000115548,ENSG00000131873,ENSG00000143970,ENSG00000169896,ENSG00000077942,ENSG00000073803,ENSG00000140575,ENSG00000153002,ENSG00000164430,ENSG00000198162,ENSG00000153317,ENSG00000105701,ENSG00000138798,ENSG00000166501,ENSG00000157399,ENSG00000198604,ENSG00000091542,ENSG00000091704,ENSG00000197442,ENSG00000173068,ENSG00000079841,ENSG00000156886,ENSG00000106571,ENSG00000175535,ENSG00000090861,ENSG00000181031,ENSG00000111087,ENSG00000186635,ENSG00000169851,ENSG00000102189,ENSG00000160094,ENSG00000213639,ENSG00000077044,ENSG00000243480,ENSG00000116833,ENSG00000048052,ENSG00000090376,ENSG00000118322,ENSG00000127152,ENSG00000168487,ENSG00000067057,ENSG00000140470,ENSG00000110395,ENSG00000169946,ENSG00000151693,ENSG00000187021,ENSG00000240038
molecular_function	anion binding	54	24.88479263		GO:0043168	ENSG00000143376,ENSG00000197442,ENSG00000197102,ENSG00000154930,ENSG00000145819,ENSG00000157107,ENSG00000124813,ENSG00000166501,ENSG00000138119,ENSG00000129566,ENSG00000100116,ENSG00000153317,ENSG00000164430,ENSG00000103196,ENSG00000100077,ENSG00000171105,ENSG00000140575,ENSG00000086991,ENSG00000073803,ENSG00000154310,ENSG00000077782,ENSG00000133392,ENSG00000038382,ENSG00000171517,ENSG00000148498,ENSG00000159216,ENSG00000080603,ENSG00000067057,ENSG00000118322,ENSG00000090376,ENSG00000130208,ENSG00000164292,ENSG00000169398,ENSG00000066468,ENSG00000116833,ENSG00000124126,ENSG00000243480,ENSG00000129515,ENSG00000134815,ENSG00000157483,ENSG00000077044,ENSG00000167601,ENSG00000102189,ENSG00000186635,ENSG00000134909,ENSG00000144426,ENSG00000130529,ENSG00000107957,ENSG00000135090,ENSG00000173575,ENSG00000090861,ENSG00000058091,ENSG00000184640,ENSG00000160087
molecular_function	cation binding	55	25.34562212		GO:0043169	ENSG00000131873,ENSG00000115548,ENSG00000148516,ENSG00000116514,ENSG00000005844,ENSG00000167460,ENSG00000177463,ENSG00000072657,ENSG00000153002,ENSG00000164430,ENSG00000140575,ENSG00000073803,ENSG00000077942,ENSG00000169896,ENSG00000143970,ENSG00000157399,ENSG00000166501,ENSG00000138798,ENSG00000105701,ENSG00000198162,ENSG00000153317,ENSG00000173068,ENSG00000197442,ENSG00000079841,ENSG00000154930,ENSG00000091704,ENSG00000198604,ENSG00000091542,ENSG00000090861,ENSG00000175535,ENSG00000106571,ENSG00000156886,ENSG00000160094,ENSG00000102189,ENSG00000169851,ENSG00000186635,ENSG00000111087,ENSG00000181031,ENSG00000090376,ENSG00000118322,ENSG00000130208,ENSG00000048052,ENSG00000116833,ENSG00000243480,ENSG00000077044,ENSG00000213639,ENSG00000240038,ENSG00000187021,ENSG00000151693,ENSG00000169946,ENSG00000110395,ENSG00000067057,ENSG00000140470,ENSG00000168487,ENSG00000127152
molecular_function	heterocyclic compound binding	63	29.03225806		GO:1901363	ENSG00000134815,ENSG00000077044,ENSG00000157483,ENSG00000167601,ENSG00000118322,ENSG00000090376,ENSG00000141298,ENSG00000169398,ENSG00000164292,ENSG00000066468,ENSG00000116833,ENSG00000111269,ENSG00000159216,ENSG00000080603,ENSG00000180530,ENSG00000067057,ENSG00000067955,ENSG00000127152,ENSG00000124571,ENSG00000169946,ENSG00000173575,ENSG00000090861,ENSG00000184640,ENSG00000058091,ENSG00000160087,ENSG00000106571,ENSG00000130529,ENSG00000135090,ENSG00000110693,ENSG00000111087,ENSG00000116350,ENSG00000160094,ENSG00000129566,ENSG00000105701,ENSG00000119314,ENSG00000166501,ENSG00000106049,ENSG00000124813,ENSG00000105419,ENSG00000110925,ENSG00000159692,ENSG00000197102,ENSG00000197442,ENSG00000173068,ENSG00000154930,ENSG00000100485,ENSG00000148516,ENSG00000124788,ENSG00000177463,ENSG00000105556,ENSG00000077782,ENSG00000115548,ENSG00000133392,ENSG00000038382,ENSG00000119686,ENSG00000073803,ENSG00000143970,ENSG00000154310,ENSG00000114439,ENSG00000164430,ENSG00000100077,ENSG00000171105,ENSG00000086991

molecular_function	organic cyclic compound binding	63	29.03225806		GO:0097159	ENSG00000154930,ENSG00000197102,ENSG00000197442,ENSG00000173068,ENSG00000100485,ENSG00000105419,ENSG00000124813,ENSG00000159692,ENSG00000110925,ENSG00000166501,ENSG00000119314,ENSG00000106049,ENSG00000105701,ENSG00000129566,ENSG00000171105,ENSG00000100077,ENSG00000114439,ENSG00000164430,ENSG00000086991,ENSG00000073803,ENSG00000119686,ENSG00000154310,ENSG00000143970,ENSG00000115548,ENSG00000077782,ENSG00000038382,ENSG00000133392,ENSG00000124788,ENSG00000148516,ENSG00000105556,ENSG00000177463,ENSG00000169946,ENSG00000124571,ENSG00000080603,ENSG00000180530,ENSG00000159216,ENSG00000111269,ENSG00000127152,ENSG00000067955,ENSG00000067057,ENSG00000118322,ENSG00000090376,ENSG00000116833,ENSG00000169398,ENSG00000164292,ENSG00000066468,ENSG00000141298,ENSG00000077044,ENSG00000157483,ENSG00000134815,ENSG00000167601,ENSG00000160094,ENSG00000116350,ENSG00000111087,ENSG00000130529,ENSG00000110693,ENSG00000135090,ENSG00000173575,ENSG00000090861,ENSG00000160087,ENSG00000106571,ENSG00000184640,ENSG00000058091
molecular_function	catalytic activity	76	35.02304147		GO:0003824	ENSG00000118322,ENSG00000048052,ENSG00000066468,ENSG00000141298,ENSG00000134815,ENSG00000077044,ENSG00000167601,ENSG00000240038,ENSG00000110395,ENSG00000080603,ENSG00000140470,ENSG00000168487,ENSG00000173575,ENSG00000184640,ENSG00000058091,ENSG00000175497,ENSG00000142615,ENSG00000128849,ENSG00000155660,ENSG00000150938,ENSG00000143811,ENSG00000138798,ENSG00000157399,ENSG00000106049,ENSG00000105701,ENSG00000167004,ENSG00000169902,ENSG00000091704,ENSG00000174939,ENSG00000091542,ENSG00000115548,ENSG00000133392,ENSG00000116514,ENSG00000153002,ENSG00000164430,ENSG00000099139,ENSG00000086991,ENSG00000154310,ENSG00000090376,ENSG00000169398,ENSG00000164292,ENSG00000243480,ENSG00000157483,ENSG00000213639,ENSG00000187021,ENSG00000067057,ENSG00000135090,ENSG00000090861,ENSG00000175535,ENSG00000215704,ENSG00000142789,ENSG00000166501,ENSG00000198380,ENSG00000100116,ENSG00000129566,ENSG00000219073,ENSG00000198162,ENSG00000197442,ENSG00000197102,ENSG00000162482,ENSG00000154930,ENSG00000159692,ENSG00000143797,ENSG00000266200,ENSG00000077782,ENSG00000216490,ENSG00000131873,ENSG00000038382,ENSG00000072657,ENSG00000171105,ENSG00000121964,ENSG00000100077,ENSG00000050748,ENSG00000118961,ENSG00000258539,ENSG00000073803
molecular_function	ion binding	92	42.39631336		GO:0043167	ENSG00000107957,ENSG00000144426,ENSG00000156886,ENSG00000058091,ENSG00000184640,ENSG00000173575,ENSG00000186635,ENSG00000169851,ENSG00000134909,ENSG00000111087,ENSG00000048052,ENSG00000066468,ENSG00000118322,ENSG00000130208,ENSG00000167601,ENSG00000077044,ENSG00000134815,ENSG00000110395,ENSG00000169946,ENSG00000240038,ENSG00000168487,ENSG00000127152,ENSG00000140470,ENSG00000080603,ENSG00000133392,ENSG00000115548,ENSG00000167460,ENSG00000171517,ENSG00000116514,ENSG00000086991,ENSG00000140575,ENSG00000164430,ENSG00000103196,ENSG00000153002,ENSG00000154310,ENSG00000143970,ENSG00000077942,ENSG00000169896,ENSG00000138798,ENSG00000157399,ENSG00000153317,ENSG00000105701,ENSG00000145819,ENSG00000079841,ENSG00000173068,ENSG00000198604,ENSG00000091542,ENSG00000124813,ENSG00000091704,ENSG00000135090,ENSG00000130529,ENSG00000160087,ENSG00000106571,ENSG00000175535,ENSG00000090861,ENSG00000160094,ENSG00000102189,ENSG00000181031,ENSG00000124126,ENSG00000116833,ENSG00000164292,ENSG00000169398,ENSG00000090376,ENSG00000213639,ENSG00000157483,ENSG00000129515,ENSG00000243480,ENSG00000148498,ENSG00000151693,ENSG00000187021,ENSG00000067057,ENSG00000159216,ENSG00000038382,ENSG00000131873,ENSG00000077782,ENSG00000072657,ENSG00000177463,ENSG00000005844,ENSG00000148516,ENSG00000100077,ENSG00000171105,ENSG00000073803,ENSG00000138119,ENSG00000166501,ENSG00000198162,ENSG00000129566,ENSG00000100116,ENSG00000157107,ENSG00000154930,ENSG00000143376,ENSG00000197442,ENSG00000197102
molecular_function	protein binding	144	66.359447		GO:0005515	ENSG00000145819,ENSG00000079841,ENSG00000167004,ENSG00000198604,ENSG00000136048,ENSG00000131236,ENSG00000124813,ENSG00000138798,ENSG00000164430,ENSG00000148935,ENSG00000140575,ENSG00000169896,ENSG00000154310,ENSG00000129116,ENSG00000171517,ENSG00000175899,ENSG00000071537,ENSG00000127152,ENSG00000180530,ENSG00000141298,ENSG00000176390,ENSG00000197256,ENSG00000167601,ENSG00000077044,ENSG00000124942,ENSG00000169851,ENSG00000186635,ENSG00000169862,ENSG00000110075,ENSG00000173575,ENSG00000213892,ENSG00000157107,ENSG00000197102,ENSG00000162482,ENSG00000154930,ENSG00000159692,ENSG00000115221,ENSG00000138119,ENSG00000166501,ENSG00000129566,ENSG00000171105,ENSG00000106070,ENSG00000073803,ENSG00000138674,ENSG00000138434,ENSG00000038382,ENSG00000169180,ENSG00000005844,ENSG00000148498,ENSG00000124571,ENSG00000151693,ENSG00000067057,ENSG00000147251,ENSG00000067955,ENSG00000124164,ENSG00000116833,ENSG00000090376,ENSG00000129515,ENSG00000157483,ENSG00000135525,ENSG00000139514,ENSG00000105329,ENSG00000198879,ENSG00000160087,ENSG00000090861,ENSG00000100485,ENSG00000054611,ENSG00000173040,ENSG00000153317,ENSG00000105701,ENSG00000111490,ENSG00000086991,ENSG00000136040,ENSG00000115109,ENSG00000099139,ENSG00000077942,ENSG00000143970,ENSG00000076356,ENSG00000133392,ENSG00000115548,ENSG00000167460,ENSG00000116514,ENSG00000169946,ENSG00000110880,ENSG00000110395,ENSG00000057019,ENSG00000064666,ENSG00000168487,ENSG00000080603,ENSG00000048052,ENSG00000066468,ENSG00000150477,ENSG00000114554,ENSG00000118322,ENSG00000153721,ENSG00000124006,ENSG00000100099,ENSG00000135052,ENSG00000080493,ENSG00000134909,ENSG00000150938,ENSG00000111087,ENSG00000155660,ENSG00000107957,ENSG00000179715,ENSG00000110693,ENSG00000184640,ENSG00000058091,ENSG00000025796,ENSG00000136237,ENSG00000163947,ENSG00000197442,ENSG00000143376,ENSG00000170927,ENSG00000110925,ENSG00000075223,ENSG00000105176,ENSG00000130956,ENSG00000050748,ENSG00000130429,ENSG00000216490,ENSG00000077782,ENSG00000138709,ENSG00000177463,ENSG00000148516,ENSG00000124788,ENSG00000146555,ENSG00000100068,ENSG00000159216,ENSG00000013364,ENSG00000164292,ENSG00000169398,ENSG00000124126,ENSG00000099282,ENSG00000196526,ENSG00000164483,ENSG00000213639,ENSG00000143768,ENSG00000102189,ENSG00000008294,ENSG00000181031,ENSG00000135090,ENSG00000130529,ENSG00000106571

molecular_function	binding	176	81.10599078		GO:0005488	ENSG00000159216,ENSG00000013364,ENSG00000100068,ENSG00000146555,ENSG00000213639,ENSG00000143768,ENSG00000099282,ENSG00000164483,ENSG00000196526,ENSG00000169398,ENSG00000164292,ENSG00000124126,ENSG00000181031,ENSG00000008294,ENSG00000102189,ENSG00000175535,ENSG00000106571,ENSG00000130529,ENSG00000135090,ENSG00000105176,ENSG00000110925,ENSG00000075223,ENSG00000163947,ENSG00000197442,ENSG00000143376,ENSG00000170927,ENSG00000100116,ENSG00000198162,ENSG00000119314,ENSG00000119686,ENSG00000100077,ENSG00000130956,ENSG00000050748,ENSG00000148516,ENSG00000124788,ENSG00000177463,ENSG00000072657,ENSG00000216490,ENSG00000077782,ENSG00000131873,ENSG00000138709,ENSG00000130429,ENSG00000080603,ENSG00000064666,ENSG00000057019,ENSG00000168487,ENSG00000169946,ENSG00000110880,ENSG00000110395,ENSG00000134815,ENSG00000135052,ENSG00000124006,ENSG00000100099,ENSG00000118322,ENSG00000114554,ENSG00000130208,ENSG00000153721,ENSG00000048052,ENSG00000150477,ENSG00000066468,ENSG00000150938,ENSG00000111087,ENSG00000155660,ENSG00000134909,ENSG00000080493,ENSG00000136237,ENSG00000184640,ENSG00000058091,ENSG00000025796,ENSG00000156886,ENSG00000144426,ENSG00000107957,ENSG00000179715,ENSG00000110693,ENSG00000105419,ENSG00000054611,ENSG00000173068,ENSG00000100485,ENSG00000105701,ENSG00000153317,ENSG00000173040,ENSG00000157399,ENSG00000106049,ENSG00000076356,ENSG00000077942,ENSG00000143970,ENSG00000103196,ENSG00000115109,ENSG00000099139,ENSG00000111490,ENSG00000086991,ENSG00000136040,ENSG00000116514,ENSG00000167460,ENSG00000136478,ENSG00000115548,ENSG00000133392,ENSG00000111269,ENSG00000147251,ENSG00000067057,ENSG00000067955,ENSG00000187021,ENSG00000151693,ENSG00000124571,ENSG00000148498,ENSG00000129515,ENSG00000243480,ENSG00000157483,ENSG00000135525,ENSG00000090376,ENSG00000124164,ENSG00000116833,ENSG00000139514,ENSG00000160094,ENSG00000090861,ENSG00000198879,ENSG00000160087,ENSG00000105329,ENSG00000159692,ENSG00000197102,ENSG00000162482,ENSG00000154930,ENSG00000157107,ENSG00000129566,ENSG00000166501,ENSG00000138119,ENSG00000115221,ENSG00000106070,ENSG00000073803,ENSG00000138674,ENSG00000171105,ENSG00000005844,ENSG00000174238,ENSG00000169180,ENSG00000138434,ENSG00000038382,ENSG00000180530,ENSG00000140470,ENSG00000127152,ENSG00000071537,ENSG00000240038,ENSG00000175899,ENSG00000077044,ENSG00000167601,ENSG00000197256,ENSG00000141298,ENSG00000176390,ENSG00000110075,ENSG00000169862,ENSG00000116350,ENSG00000124942,ENSG00000186635,ENSG00000169851,ENSG00000173575,ENSG00000213892,ENSG00000131236,ENSG00000136048,ENSG00000091704,ENSG00000124813,ENSG00000091542,ENSG00000198604,ENSG00000079841,ENSG00000167004,ENSG00000145819,ENSG00000146433,ENSG00000138798,ENSG00000169896,ENSG00000154310,ENSG00000164430,ENSG00000153002,ENSG00000148935,ENSG00000114439,ENSG00000140575,ENSG00000171517,ENSG00000105556,ENSG00000129116
molecular_function	molecular_function	205	94.47004608		GO:0003674	ENSG00000164292,ENSG00000169398,ENSG00000124126,ENSG00000152078,ENSG00000099282,ENSG00000164483,ENSG00000196526,ENSG00000143768,ENSG00000213639,ENSG00000146555,ENSG00000100068,ENSG00000159216,ENSG00000013364,ENSG00000135090,ENSG00000130529,ENSG00000168389,ENSG00000106571,ENSG00000215704,ENSG00000175535,ENSG00000102189,ENSG00000008294,ENSG00000142789,ENSG00000181031,ENSG00000198380,ENSG00000119314,ENSG00000198162,ENSG00000100116,ENSG00000143376,ENSG00000197442,ENSG00000163947,ENSG00000170927,ENSG00000075223,ENSG00000110925,ENSG00000266200,ENSG00000105176,ENSG00000130429,ENSG00000131873,ENSG00000216490,ENSG00000077782,ENSG00000138709,ENSG00000177463,ENSG00000072657,ENSG00000148516,ENSG00000124788,ENSG00000130956,ENSG00000050748,ENSG00000100077,ENSG00000119686,ENSG00000258539,ENSG00000150477,ENSG00000066468,ENSG00000048052,ENSG00000130208,ENSG00000153721,ENSG00000118322,ENSG00000114554,ENSG00000139540,ENSG00000124006,ENSG00000100099,ENSG00000134815,ENSG00000135052,ENSG00000169946,ENSG00000110880,ENSG00000110395,ENSG00000169359,ENSG00000057019,ENSG00000064666,ENSG00000168487,ENSG00000080603,ENSG00000107957,ENSG00000179715,ENSG00000110693,ENSG00000144426,ENSG00000058091,ENSG00000184640,ENSG00000025796,ENSG00000156886,ENSG00000136237,ENSG00000080493,ENSG00000134909,ENSG00000155660,ENSG00000150938,ENSG00000111087,ENSG00000106049,ENSG00000173040,ENSG00000157399,ENSG00000153317,ENSG00000105701,ENSG00000100485,ENSG00000054611,ENSG00000173068,ENSG00000169902,ENSG00000105419,ENSG00000133392,ENSG00000115548,ENSG00000136478,ENSG00000167460,ENSG00000116514,ENSG00000111490,ENSG00000086991,ENSG00000136040,ENSG00000103196,ENSG00000115109,ENSG00000099139,ENSG00000077942,ENSG00000143970,ENSG00000076356,ENSG00000124164,ENSG00000116833,ENSG00000090376,ENSG00000243480,ENSG00000129515,ENSG00000157483,ENSG00000135525,ENSG00000148498,ENSG00000124571,ENSG00000187021,ENSG00000151693,ENSG00000147251,ENSG00000067057,ENSG00000067955,ENSG00000111269,ENSG00000105329,ENSG00000198879,ENSG00000033867,ENSG00000160087,ENSG00000090861,ENSG00000160094,ENSG00000139514,ENSG00000115221,ENSG00000138119,ENSG00000166501,ENSG00000219073,ENSG00000129566,ENSG00000157107,ENSG00000197102,ENSG00000162482,ENSG00000154930,ENSG00000159692,ENSG00000143797,ENSG00000138434,ENSG00000038382,ENSG00000169180,ENSG00000174238,ENSG00000005844,ENSG00000156011,ENSG00000112394,ENSG00000235568,ENSG00000121964,ENSG00000171105,ENSG00000106070,ENSG00000118961,ENSG00000073803,ENSG00000138674,ENSG00000141298,ENSG00000176390,ENSG00000197256,ENSG00000167601,ENSG00000077044,ENSG00000240038,ENSG00000175899,ENSG00000140470,ENSG00000071537,ENSG00000127152,ENSG00000180530,ENSG00000159128,ENSG00000101187,ENSG00000173575,ENSG00000213892,ENSG00000072954,ENSG00000124942,ENSG00000186635,ENSG00000128849,ENSG00000169851,ENSG00000138640,ENSG00000175497,ENSG00000116350,ENSG00000142615,ENSG00000169862,ENSG00000110075,ENSG00000143811,ENSG00000138798,ENSG00000106771,ENSG00000146433,ENSG00000145819,ENSG00000167004,ENSG00000079841,ENSG00000174939,ENSG00000091542,ENSG00000198604,ENSG00000131236,ENSG00000136048,ENSG00000091704,ENSG00000124813,ENSG00000129116,ENSG00000105556,ENSG00000171517,ENSG00000148935,ENSG00000164430,ENSG00000153002,ENSG00000114439,ENSG00000140575,ENSG00000169896,ENSG00000154310,ENSG00000031003
cellular_component	membrane part	82	37.78801843		GO:0044425	ENSG00000171105,ENSG00000112394,ENSG00000235568,ENSG00000119686,ENSG00000138674,ENSG00000077782,ENSG00000131873,ENSG00000095139,ENSG00000005844,ENSG00000072657,ENSG00000197442,ENSG00000143376,ENSG00000170927,ENSG00000157107,ENSG00000143797,ENSG00000138119,ENSG00000115221,ENSG00000198162,ENSG00000102189,ENSG00000139514,ENSG00000008294,ENSG00000130529,ENSG00000188158,ENSG00000168389,ENSG00000033867,ENSG00000160087,ENSG00000146555,ENSG00000013364,ENSG00000169398,ENSG00000124164,ENSG00000152078,ENSG00000129515,ENSG00000135525,ENSG00000099282,ENSG00000115109,ENSG00000140575,ENSG00000099139,ENSG00000086991,ENSG00000136040,ENSG00000076356,ENSG00000169896,ENSG00000154310,ENSG00000136478,ENSG00000116514,ENSG00000171517,ENSG00000169902,ENSG00000136048,ENSG00000172765,ENSG00000164073,ENSG00000174939,ENSG00000173040,ENSG00000138798,ENSG00000105701,ENSG00000181826,ENSG00000106771,ENSG00000146433,ENSG00000175497,ENSG00000080493,ENSG00000124942,ENSG00000072954,ENSG00000169851,ENSG00000150938,ENSG00000134909,ENSG00000075420,ENSG00000144426,ENSG00000100731,ENSG00000159128,ENSG00000101187,ENSG00000103534,ENSG00000025796,ENSG00000156886,ENSG00000110880,ENSG00000110395,ENSG00000169359,ENSG00000057019,ENSG00000071537,ENSG00000114554,ENSG00000118322,ENSG00000066468,ENSG00000135052,ENSG00000167601,ENSG00000139540

cellular_component	intracellular organelle part	104	47.92626728		GO:0044446	ENSG00000102189,ENSG00000008294,ENSG00000134369,ENSG00000181031,ENSG00000105329,ENSG00000130529,ENSG00000168389,ENSG00000198879,ENSG00000160087,ENSG00000106571,ENSG00000124571,ENSG00000151693,ENSG00000067955,ENSG00000159216,ENSG00000013364,ENSG00000169398,ENSG00000124164,ENSG00000116833,ENSG00000129515,ENSG00000157483,ENSG00000213639,ENSG00000135525,ENSG00000143768,ENSG00000156011,ENSG00000130956,ENSG00000050748,ENSG00000171105,ENSG00000138674,ENSG00000130429,ENSG00000216490,ENSG00000131873,ENSG00000169180,ENSG00000177463,ENSG00000148516,ENSG00000095139,ENSG00000124788,ENSG00000197102,ENSG00000143376,ENSG00000154930,ENSG00000170927,ENSG00000159692,ENSG00000143797,ENSG00000105176,ENSG00000138119,ENSG00000166501,ENSG00000198162,ENSG00000129566,ENSG00000100116,ENSG00000072954,ENSG00000124942,ENSG00000186635,ENSG00000169851,ENSG00000128849,ENSG00000116350,ENSG00000134909,ENSG00000155660,ENSG00000110075,ENSG00000111087,ENSG00000107957,ENSG00000110693,ENSG00000159128,ENSG00000184640,ENSG00000025796,ENSG00000173575,ENSG00000213892,ENSG00000169946,ENSG00000169359,ENSG00000175899,ENSG00000064666,ENSG00000071537,ENSG00000180530,ENSG00000066468,ENSG00000048052,ENSG00000118322,ENSG00000124006,ENSG00000111490,ENSG00000086991,ENSG00000148935,ENSG00000114439,ENSG00000140575,ENSG00000099139,ENSG00000196141,ENSG00000154310,ENSG00000143970,ENSG00000133392,ENSG00000129116,ENSG00000115548,ENSG00000167460,ENSG00000104671,ENSG00000167004,ENSG00000169902,ENSG00000091542,ENSG00000198604,ENSG00000136048,ENSG00000172765,ENSG00000131236,ENSG00000164073,ENSG00000124813,ENSG00000106049,ENSG00000143811,ENSG00000157399,ENSG00000173040,ENSG00000138798,ENSG00000105701
cellular_component	organelle part	106	48.84792627		GO:0044422	ENSG00000102189,ENSG00000008294,ENSG00000181031,ENSG00000134369,ENSG00000105329,ENSG00000168389,ENSG00000130529,ENSG00000106571,ENSG00000198879,ENSG00000160087,ENSG00000033867,ENSG00000124571,ENSG00000151693,ENSG00000067955,ENSG00000013364,ENSG00000159216,ENSG00000116833,ENSG00000124164,ENSG00000169398,ENSG00000164292,ENSG00000213639,ENSG00000135525,ENSG00000157483,ENSG00000143768,ENSG00000129515,ENSG00000130956,ENSG00000156011,ENSG00000050748,ENSG00000171105,ENSG00000138674,ENSG00000130429,ENSG00000169180,ENSG00000131873,ENSG00000216490,ENSG00000177463,ENSG00000124788,ENSG00000095139,ENSG00000148516,ENSG00000154930,ENSG00000170927,ENSG00000143376,ENSG00000197102,ENSG00000143797,ENSG00000159692,ENSG00000105176,ENSG00000138119,ENSG00000166501,ENSG00000198162,ENSG00000100116,ENSG00000129566,ENSG00000128849,ENSG00000186635,ENSG00000169851,ENSG00000072954,ENSG00000124942,ENSG00000116350,ENSG00000134909,ENSG00000155660,ENSG00000111087,ENSG00000110075,ENSG00000110693,ENSG00000107957,ENSG00000159128,ENSG00000025796,ENSG00000184640,ENSG00000213892,ENSG00000173575,ENSG00000169359,ENSG00000169946,ENSG00000175899,ENSG00000071537,ENSG00000064666,ENSG00000180530,ENSG00000048052,ENSG00000066468,ENSG00000118322,ENSG00000124006,ENSG00000086991,ENSG00000111490,ENSG00000140575,ENSG00000099139,ENSG00000148935,ENSG00000114439,ENSG00000143970,ENSG00000154310,ENSG00000196141,ENSG00000129116,ENSG00000133392,ENSG00000115548,ENSG00000167460,ENSG00000104671,ENSG00000169902,ENSG00000167004,ENSG00000198604,ENSG00000091542,ENSG00000164073,ENSG00000124813,ENSG00000131236,ENSG00000172765,ENSG00000136048,ENSG00000106049,ENSG00000173040,ENSG00000157399,ENSG00000138798,ENSG00000143811,ENSG00000105701
cellular_component	cytoplasmic part	109	50.23041475		GO:0044444	ENSG00000130956,ENSG00000050748,ENSG00000171105,ENSG00000138674,ENSG00000073803,ENSG00000118961,ENSG00000106070,ENSG00000038382,ENSG00000130429,ENSG00000131873,ENSG00000216490,ENSG00000077782,ENSG00000095139,ENSG00000157107,ENSG00000170927,ENSG00000154930,ENSG00000197442,ENSG00000163947,ENSG00000197102,ENSG00000143376,ENSG00000162482,ENSG00000143797,ENSG00000105176,ENSG00000198380,ENSG00000138119,ENSG00000166501,ENSG00000198162,ENSG00000100116,ENSG00000102189,ENSG00000008294,ENSG00000181031,ENSG00000105329,ENSG00000168389,ENSG00000130529,ENSG00000106571,ENSG00000160087,ENSG00000033867,ENSG00000090861,ENSG00000148498,ENSG00000124571,ENSG00000151693,ENSG00000067057,ENSG00000147251,ENSG00000013364,ENSG00000124126,ENSG00000124164,ENSG00000169398,ENSG00000164292,ENSG00000213639,ENSG00000135525,ENSG00000157483,ENSG00000143768,ENSG00000129515,ENSG00000086991,ENSG00000140575,ENSG00000099139,ENSG00000164430,ENSG00000148935,ENSG00000103196,ENSG00000154310,ENSG00000031003,ENSG00000129116,ENSG00000133392,ENSG00000136478,ENSG00000167460,ENSG00000116514,ENSG00000145819,ENSG00000104671,ENSG00000100485,ENSG00000169902,ENSG00000079841,ENSG00000167004,ENSG00000164073,ENSG00000172765,ENSG00000131236,ENSG00000136048,ENSG00000106049,ENSG00000138798,ENSG00000157399,ENSG00000143811,ENSG00000153317,ENSG00000105701,ENSG00000169851,ENSG00000186635,ENSG00000138640,ENSG00000072954,ENSG00000124942,ENSG00000134909,ENSG00000155660,ENSG00000110075,ENSG00000111087,ENSG00000159128,ENSG00000058091,ENSG00000184640,ENSG00000025796,ENSG00000110395,ENSG00000169359,ENSG00000175899,ENSG00000071537,ENSG00000168487,ENSG00000080603,ENSG00000066468,ENSG00000130208,ENSG00000197256,ENSG00000118322,ENSG00000100099,ENSG00000124006,ENSG00000135052,ENSG00000134815
cellular_component	membrane	121	55.76036866		GO:0016020	ENSG00000130529,ENSG00000188158,ENSG00000168389,ENSG00000105329,ENSG00000135090,ENSG00000090861,ENSG00000033867,ENSG00000160087,ENSG00000102189,ENSG00000181031,ENSG00000139514,ENSG00000008294,ENSG00000164292,ENSG00000169398,ENSG00000152078,ENSG00000124126,ENSG00000124164,ENSG00000129515,ENSG00000135525,ENSG00000099282,ENSG00000146555,ENSG00000151693,ENSG00000148498,ENSG00000013364,ENSG00000067057,ENSG00000100068,ENSG00000067955,ENSG00000131873,ENSG00000216490,ENSG00000077782,ENSG00000169180,ENSG00000138434,ENSG00000005844,ENSG00000095139,ENSG00000072657,ENSG00000171105,ENSG00000100077,ENSG00000156011,ENSG00000112394,ENSG00000235568,ENSG00000119686,ENSG00000106070,ENSG00000138674,ENSG00000073803,ENSG00000166501,ENSG00000138119,ENSG00000115221,ENSG00000100116,ENSG00000198162,ENSG00000197442,ENSG00000197102,ENSG00000143376,ENSG00000170927,ENSG00000157107,ENSG00000143797,ENSG00000144426,ENSG00000100731,ENSG00000101187,ENSG00000159128,ENSG00000103534,ENSG00000058091,ENSG00000025796,ENSG00000156886,ENSG00000175497,ENSG00000080493,ENSG00000072954,ENSG00000124942,ENSG00000186635,ENSG00000169851,ENSG00000150938,ENSG00000110075,ENSG00000134909,ENSG00000075420,ENSG00000114554,ENSG00000153721,ENSG00000118322,ENSG00000066468,ENSG00000176390,ENSG00000134815,ENSG00000135052,ENSG00000077044,ENSG00000167601,ENSG00000139540,ENSG00000100099,ENSG00000110880,ENSG00000169359,ENSG00000110395,ENSG00000064666,ENSG00000057019,ENSG00000071537,ENSG00000136478,ENSG00000115548,ENSG00000129116,ENSG00000116514,ENSG00000171517,ENSG00000167460,ENSG00000115109,ENSG00000148935,ENSG00000099139,ENSG00000140575,ENSG00000111490,ENSG00000086991,ENSG00000136040,ENSG00000076356,ENSG00000169896,ENSG00000154310,ENSG00000173040,ENSG00000138798,ENSG00000181826,ENSG00000105701,ENSG00000153317,ENSG00000146433,ENSG00000106771,ENSG00000079841,ENSG00000173068,ENSG00000169902,ENSG00000136048,ENSG00000172765,ENSG00000131236,ENSG00000164073,ENSG00000174939

cellular_component	intracellular membrane-bounded organelle	124	57.14285714		GO:0043231	ENSG00000186635,ENSG00000169851,ENSG00000124942,ENSG00000072954,ENSG00000116350,ENSG00000169862,ENSG00000134909,ENSG00000155660,ENSG00000111087,ENSG00000110075,ENSG00000110693,ENSG00000159128,ENSG00000184640,ENSG00000058091,ENSG00000025796,ENSG00000136237,ENSG00000173575,ENSG00000169359,ENSG00000110395,ENSG00000169946,ENSG00000175899,ENSG00000168487,ENSG00000127152,ENSG00000071537,ENSG00000180530,ENSG00000080603,ENSG00000066468,ENSG00000048052,ENSG00000130208,ENSG00000197256,ENSG00000114554,ENSG00000118322,ENSG00000100099,ENSG00000124006,ENSG00000135052,ENSG00000134815,ENSG00000086991,ENSG00000111490,ENSG00000099139,ENSG00000140575,ENSG00000114439,ENSG00000103196,ENSG00000115109,ENSG00000143970,ENSG00000154310,ENSG00000196141,ENSG00000129116,ENSG00000133392,ENSG00000136478,ENSG00000115548,ENSG00000105556,ENSG00000116514,ENSG00000169902,ENSG00000167004,ENSG00000173068,ENSG00000198604,ENSG00000091542,ENSG00000164073,ENSG00000105419,ENSG00000124813,ENSG00000172765,ENSG00000136048,ENSG00000106049,ENSG00000173040,ENSG00000157399,ENSG00000138798,ENSG00000143811,ENSG00000105701,ENSG00000160094,ENSG00000102189,ENSG00000008294,ENSG00000181031,ENSG00000105329,ENSG00000168389,ENSG00000130529,ENSG00000160087,ENSG00000198879,ENSG00000106571,ENSG00000124571,ENSG00000151693,ENSG00000067955,ENSG00000067057,ENSG00000013364,ENSG00000111269,ENSG00000159216,ENSG00000124164,ENSG00000124126,ENSG00000116833,ENSG00000164292,ENSG00000169398,ENSG00000090376,ENSG00000157483,ENSG00000213639,ENSG00000143768,ENSG00000129515,ENSG00000130956,ENSG00000050748,ENSG00000171105,ENSG00000138674,ENSG00000118961,ENSG00000138434,ENSG00000169180,ENSG00000138709,ENSG00000216490,ENSG00000077782,ENSG00000131873,ENSG00000177463,ENSG00000124788,ENSG00000095139,ENSG00000148516,ENSG00000157107,ENSG00000154930,ENSG00000170927,ENSG00000143376,ENSG00000143797,ENSG00000110925,ENSG00000159692,ENSG00000105176,ENSG00000138119,ENSG00000166501,ENSG00000119314,ENSG00000198162,ENSG00000100116,ENSG00000129566
cellular_component	cytoplasm	141	64.97695853		GO:0005737	ENSG00000066468,ENSG00000048052,ENSG00000130208,ENSG00000114554,ENSG00000153721,ENSG00000118322,ENSG00000100099,ENSG00000124006,ENSG00000135052,ENSG00000134815,ENSG00000169359,ENSG00000110395,ENSG00000110880,ENSG00000169946,ENSG00000168487,ENSG00000080603,ENSG00000107957,ENSG00000184640,ENSG00000058091,ENSG00000025796,ENSG00000134909,ENSG00000111087,ENSG00000155660,ENSG00000106049,ENSG00000157399,ENSG00000173040,ENSG00000153317,ENSG00000105701,ENSG00000104671,ENSG00000100485,ENSG00000169902,ENSG00000173068,ENSG00000133392,ENSG00000136478,ENSG00000115548,ENSG00000167460,ENSG00000116514,ENSG00000086991,ENSG00000099139,ENSG00000115109,ENSG00000103196,ENSG00000196141,ENSG00000124126,ENSG00000164292,ENSG00000169398,ENSG00000196526,ENSG00000213639,ENSG00000143768,ENSG00000013364,ENSG00000159216,ENSG00000135090,ENSG00000168389,ENSG00000188158,ENSG00000130529,ENSG00000106571,ENSG00000102189,ENSG00000008294,ENSG00000181031,ENSG00000134369,ENSG00000198380,ENSG00000198162,ENSG00000100116,ENSG00000170927,ENSG00000197442,ENSG00000143376,ENSG00000163947,ENSG00000105176,ENSG00000130429,ENSG00000216490,ENSG00000131873,ENSG00000077782,ENSG00000072657,ENSG00000124788,ENSG00000095139,ENSG00000148516,ENSG00000050748,ENSG00000130956,ENSG00000176390,ENSG00000141298,ENSG00000197256,ENSG00000077044,ENSG00000175899,ENSG00000071537,ENSG00000159128,ENSG00000169851,ENSG00000186635,ENSG00000138640,ENSG00000124942,ENSG00000072954,ENSG00000169862,ENSG00000110075,ENSG00000138798,ENSG00000143811,ENSG00000145819,ENSG00000167004,ENSG00000079841,ENSG00000124813,ENSG00000164073,ENSG00000172765,ENSG00000136048,ENSG00000131236,ENSG00000129116,ENSG00000140575,ENSG00000164430,ENSG00000148935,ENSG00000114439,ENSG00000154310,ENSG00000031003,ENSG00000124164,ENSG00000116833,ENSG00000090376,ENSG00000157483,ENSG00000135525,ENSG00000129515,ENSG00000124571,ENSG00000148498,ENSG00000151693,ENSG00000147251,ENSG00000067057,ENSG00000105329,ENSG00000160087,ENSG00000033867,ENSG00000090861,ENSG00000138119,ENSG00000166501,ENSG00000129566,ENSG00000157107,ENSG00000154930,ENSG00000162482,ENSG00000197102,ENSG00000143797,ENSG00000159692,ENSG00000038382,ENSG00000138434,ENSG00000169180,ENSG00000174238,ENSG00000171105,ENSG00000138674,ENSG00000073803,ENSG00000118961,ENSG00000106070
cellular_component	intracellular organelle	144	66.359447		GO:0043229	ENSG00000168389,ENSG00000130529,ENSG00000106571,ENSG00000102189,ENSG00000181031,ENSG00000134369,ENSG00000008294,ENSG00000124126,ENSG00000164292,ENSG00000169398,ENSG00000213639,ENSG00000143768,ENSG00000196526,ENSG00000013364,ENSG00000159216,ENSG00000138709,ENSG00000077782,ENSG00000216490,ENSG00000131873,ENSG00000130429,ENSG00000095139,ENSG00000124788,ENSG00000148516,ENSG00000177463,ENSG00000050748,ENSG00000130956,ENSG00000119314,ENSG00000100116,ENSG00000198162,ENSG00000170927,ENSG00000143376,ENSG00000105176,ENSG00000110925,ENSG00000110693,ENSG00000107957,ENSG00000136237,ENSG00000025796,ENSG00000184640,ENSG00000058091,ENSG00000155660,ENSG00000111087,ENSG00000134909,ENSG00000114554,ENSG00000130208,ENSG00000118322,ENSG00000066468,ENSG00000048052,ENSG00000135052,ENSG00000134815,ENSG00000100099,ENSG00000124006,ENSG00000169359,ENSG00000110395,ENSG00000169946,ENSG00000110880,ENSG00000080603,ENSG00000168487,ENSG00000064666,ENSG00000115548,ENSG00000136478,ENSG00000133392,ENSG00000116514,ENSG00000167460,ENSG00000099139,ENSG00000103196,ENSG00000115109,ENSG00000086991,ENSG00000111490,ENSG00000143970,ENSG00000196141,ENSG00000173040,ENSG00000157399,ENSG00000106049,ENSG00000105701,ENSG00000169902,ENSG00000173068,ENSG00000104671,ENSG00000105419,ENSG00000105329,ENSG00000198879,ENSG00000033867,ENSG00000160087,ENSG00000160094,ENSG00000090376,ENSG00000124164,ENSG00000116833,ENSG00000157483,ENSG00000135525,ENSG00000129515,ENSG00000151693,ENSG00000124571,ENSG00000148498,ENSG00000111269,ENSG00000067955,ENSG00000067057,ENSG00000169180,ENSG00000138434,ENSG00000171105,ENSG00000156011,ENSG00000138674,ENSG00000118961,ENSG00000166501,ENSG00000138119,ENSG00000129566,ENSG00000154930,ENSG00000197102,ENSG00000157107,ENSG00000143797,ENSG00000159692,ENSG00000159128,ENSG00000213892,ENSG00000173575,ENSG00000116350,ENSG00000128849,ENSG00000186635,ENSG00000169851,ENSG00000124942,ENSG00000072954,ENSG00000110075,ENSG00000169862,ENSG00000197256,ENSG00000141298,ENSG00000175899,ENSG00000180530,ENSG00000071537,ENSG00000127152,ENSG00000129116,ENSG00000105556,ENSG00000140575,ENSG00000148935,ENSG00000114439,ENSG00000154310,ENSG00000138798,ENSG00000143811,ENSG00000181826,ENSG00000167004,ENSG00000145819,ENSG00000124813,ENSG00000164073,ENSG00000136048,ENSG00000172765,ENSG00000131236,ENSG00000091542,ENSG00000198604

cellular_component	membrane-bounded organelle	148	68.20276498		GO:0043227	ENSG00000160094,ENSG00000105329,ENSG00000090861,ENSG00000160087,ENSG00000198879,ENSG00000151693,ENSG00000124571,ENSG00000111269,ENSG00000067057,ENSG00000067955,ENSG00000090376,ENSG00000124164,ENSG00000116833,ENSG00000129515,ENSG00000243480,ENSG00000157483,ENSG00000171105,ENSG00000118961,ENSG00000138674,ENSG00000169180,ENSG00000138434,ENSG00000005844,ENSG00000174238,ENSG00000162482,ENSG00000197102,ENSG00000154930,ENSG00000157107,ENSG00000159692,ENSG00000143797,ENSG00000166501,ENSG00000115221,ENSG00000138119,ENSG00000129566,ENSG00000116350,ENSG00000072954,ENSG00000124942,ENSG00000186635,ENSG00000169851,ENSG00000110075,ENSG00000169862,ENSG00000159128,ENSG00000173575,ENSG00000240038,ENSG00000175899,ENSG00000180530,ENSG00000071537,ENSG00000127152,ENSG00000197256,ENSG00000167601,ENSG00000114439,ENSG00000140575,ENSG00000169896,ENSG00000154310,ENSG00000129116,ENSG00000105556,ENSG00000167004,ENSG00000136048,ENSG00000172765,ENSG00000131236,ENSG00000164073,ENSG00000124813,ENSG00000091542,ENSG00000198604,ENSG00000143811,ENSG00000138798,ENSG00000102189,ENSG00000181031,ENSG00000008294,ENSG00000130529,ENSG00000168389,ENSG00000106571,ENSG00000146555,ENSG00000159216,ENSG00000013364,ENSG00000169398,ENSG00000164292,ENSG00000124126,ENSG00000143768,ENSG00000213639,ENSG00000099282,ENSG00000050748,ENSG00000130956,ENSG00000216490,ENSG00000077782,ENSG00000131873,ENSG00000138709,ENSG00000130429,ENSG00000148516,ENSG00000124788,ENSG00000095139,ENSG00000177463,ENSG00000072657,ENSG00000143376,ENSG00000170927,ENSG00000105176,ENSG00000075223,ENSG00000110925,ENSG00000119314,ENSG00000198380,ENSG00000100116,ENSG00000198162,ENSG00000080493,ENSG00000111087,ENSG00000155660,ENSG00000150938,ENSG00000134909,ENSG00000110693,ENSG00000103534,ENSG00000136237,ENSG00000184640,ENSG00000058091,ENSG00000025796,ENSG00000169946,ENSG00000169359,ENSG00000110395,ENSG00000080603,ENSG00000064666,ENSG00000168487,ENSG00000130208,ENSG00000114554,ENSG00000118322,ENSG00000066468,ENSG00000048052,ENSG00000134815,ENSG00000135052,ENSG00000124006,ENSG00000139540,ENSG00000100099,ENSG00000115109,ENSG00000103196,ENSG00000099139,ENSG00000111490,ENSG00000086991,ENSG00000196141,ENSG00000077942,ENSG00000143970,ENSG00000136478,ENSG00000115548,ENSG00000133392,ENSG00000116514,ENSG00000167460,ENSG00000173068,ENSG00000169902,ENSG00000105419,ENSG00000157399,ENSG00000173040,ENSG00000106049,ENSG00000105701
cellular_component	organelle	163	75.11520737		GO:0043226	ENSG00000071537,ENSG00000127152,ENSG00000180530,ENSG00000175899,ENSG00000240038,ENSG00000167601,ENSG00000141298,ENSG00000197256,ENSG00000169862,ENSG00000110075,ENSG00000169851,ENSG00000186635,ENSG00000128849,ENSG00000124942,ENSG00000072954,ENSG00000116350,ENSG00000173575,ENSG00000213892,ENSG00000159128,ENSG00000198604,ENSG00000091542,ENSG00000164073,ENSG00000124813,ENSG00000131236,ENSG00000172765,ENSG00000136048,ENSG00000145819,ENSG00000167004,ENSG00000181826,ENSG00000138798,ENSG00000143811,ENSG00000154310,ENSG00000169896,ENSG00000140575,ENSG00000148935,ENSG00000114439,ENSG00000105556,ENSG00000129116,ENSG00000067955,ENSG00000067057,ENSG00000111269,ENSG00000124571,ENSG00000148498,ENSG00000151693,ENSG00000135525,ENSG00000157483,ENSG00000243480,ENSG00000129515,ENSG00000124164,ENSG00000116833,ENSG00000090376,ENSG00000160094,ENSG00000160087,ENSG00000198879,ENSG00000033867,ENSG00000090861,ENSG00000105329,ENSG00000143797,ENSG00000159692,ENSG00000157107,ENSG00000154930,ENSG00000162482,ENSG00000197102,ENSG00000129566,ENSG00000115221,ENSG00000138119,ENSG00000166501,ENSG00000138674,ENSG00000118961,ENSG00000156011,ENSG00000171105,ENSG00000174238,ENSG00000005844,ENSG00000138434,ENSG00000169180,ENSG00000168487,ENSG00000064666,ENSG00000080603,ENSG00000169359,ENSG00000110395,ENSG00000110880,ENSG00000169946,ENSG00000100099,ENSG00000139540,ENSG00000124006,ENSG00000135052,ENSG00000134815,ENSG00000066468,ENSG00000048052,ENSG00000114554,ENSG00000130208,ENSG00000118322,ENSG00000134909,ENSG00000155660,ENSG00000111087,ENSG00000150938,ENSG00000080493,ENSG00000025796,ENSG00000184640,ENSG00000058091,ENSG00000103534,ENSG00000136237,ENSG00000110693,ENSG00000107957,ENSG00000105419,ENSG00000104671,ENSG00000169902,ENSG00000173068,ENSG00000105701,ENSG00000106049,ENSG00000173040,ENSG00000157399,ENSG00000143970,ENSG00000196141,ENSG00000077942,ENSG00000111490,ENSG00000086991,ENSG00000099139,ENSG00000103196,ENSG00000115109,ENSG00000167460,ENSG00000116514,ENSG00000133392,ENSG00000115548,ENSG00000136478,ENSG00000013364,ENSG00000159216,ENSG00000146555,ENSG00000196526,ENSG00000099282,ENSG00000143768,ENSG00000213639,ENSG00000124126,ENSG00000169398,ENSG00000164292,ENSG00000008294,ENSG00000181031,ENSG00000134369,ENSG00000102189,ENSG00000106571,ENSG00000168389,ENSG00000130529,ENSG00000075223,ENSG00000110925,ENSG00000105176,ENSG00000170927,ENSG00000143376,ENSG00000198162,ENSG00000100116,ENSG00000198380,ENSG00000119314,ENSG00000050748,ENSG00000130956,ENSG00000072657,ENSG00000177463,ENSG00000124788,ENSG00000095139,ENSG00000148516,ENSG00000130429,ENSG00000138709,ENSG00000216490,ENSG00000077782,ENSG00000131873
cellular_component	intracellular part	166	76.49769585		GO:0044424	ENSG00000184640,ENSG00000058091,ENSG00000025796,ENSG00000136237,ENSG00000110693,ENSG00000107957,ENSG00000134909,ENSG00000155660,ENSG00000111087,ENSG00000100099,ENSG00000124006,ENSG00000135052,ENSG00000134815,ENSG00000048052,ENSG00000066468,ENSG00000114554,ENSG00000118322,ENSG00000130208,ENSG00000153721,ENSG00000168487,ENSG00000064666,ENSG00000080603,ENSG00000169359,ENSG00000110395,ENSG00000110880,ENSG00000169946,ENSG00000167460,ENSG00000116514,ENSG00000133392,ENSG00000115548,ENSG00000136478,ENSG00000143970,ENSG00000196141,ENSG00000111490,ENSG00000086991,ENSG00000099139,ENSG00000115109,ENSG00000103196,ENSG00000153317,ENSG00000105701,ENSG00000106049,ENSG00000157399,ENSG00000173040,ENSG00000105419,ENSG00000104671,ENSG00000100485,ENSG00000169902,ENSG00000173068,ENSG00000106571,ENSG00000135090,ENSG00000188158,ENSG00000168389,ENSG00000130529,ENSG00000008294,ENSG00000181031,ENSG00000134369,ENSG00000102189,ENSG00000196526,ENSG00000213639,ENSG00000143768,ENSG00000124126,ENSG00000164292,ENSG00000169398,ENSG00000013364,ENSG00000159216,ENSG00000072657,ENSG00000177463,ENSG00000095139,ENSG00000124788,ENSG00000148516,ENSG00000130429,ENSG00000138709,ENSG00000131873,ENSG00000216490,ENSG00000077782,ENSG00000050748,ENSG00000130956,ENSG00000198162,ENSG00000100116,ENSG00000198380,ENSG00000119314,ENSG00000110925,ENSG00000105176,ENSG00000170927,ENSG00000197442,ENSG00000163947,ENSG00000143376,ENSG00000213892,ENSG00000173575,ENSG00000159128,ENSG00000169862,ENSG00000110075,ENSG00000186635,ENSG00000138640,ENSG00000169851,ENSG00000128849,ENSG00000124942,ENSG00000072954,ENSG00000116350,ENSG00000077044,ENSG00000176390,ENSG00000141298,ENSG00000197256,ENSG00000071537,ENSG00000127152,ENSG00000180530,ENSG00000175899,ENSG00000105556,ENSG00000129116,ENSG00000154310,ENSG00000031003,ENSG00000140575,ENSG00000164430,ENSG00000114439,ENSG00000148935,ENSG00000181826,ENSG00000138798,ENSG00000143811,ENSG00000091542,ENSG00000198604,ENSG00000164073,ENSG00000124813,ENSG00000172765,ENSG00000136048,ENSG00000131236,ENSG00000145819,ENSG00000079841,ENSG00000167004,ENSG00000033867,ENSG00000198879,ENSG00000160087,ENSG00000090861,ENSG00000105329,ENSG00000160094,ENSG00000157483,ENSG00000135525,ENSG00000129515,ENSG00000124164,ENSG00000116833,ENSG00000090376,ENSG00000067955,ENSG00000147251,ENSG00000067057,ENSG00000111269,ENSG00000124571,ENSG00000148498,ENSG00000151693,ENSG00000174238,ENSG00000038382,ENSG00000138434,ENSG00000169180,ENSG00000073803,ENSG00000138674,ENSG00000118961,ENSG00000106070,ENSG00000156011,ENSG00000171105,ENSG00000129566,ENSG00000138119,ENSG00000166501,ENSG00000143797,ENSG00000159692,ENSG00000157107,ENSG00000154930,ENSG00000162482,ENSG00000197102

cellular_component	intracellular	168	77.41935484			GO:0005622	ENSG00000129566,ENSG00000138119,ENSG00000166501,ENSG00000143797,ENSG00000159692,ENSG00000157107,ENSG00000154930,ENSG00000162482,ENSG00000197102,ENSG00000174238,ENSG00000038382,ENSG00000138434,ENSG00000169180,ENSG00000073803,ENSG00000138674,ENSG00000106070,ENSG00000118961,ENSG00000235568,ENSG00000156011,ENSG00000171105,ENSG00000135525,ENSG00000157483,ENSG00000129515,ENSG00000124164,ENSG00000116833,ENSG00000090376,ENSG00000067955,ENSG00000067057,ENSG00000147251,ENSG00000111269,ENSG00000124571,ENSG00000148498,ENSG00000151693,ENSG00000198879,ENSG00000160087,ENSG00000033867,ENSG00000090861,ENSG00000105329,ENSG00000160094,ENSG00000181826,ENSG00000138798,ENSG00000143811,ENSG00000091542,ENSG00000198604,ENSG00000164073,ENSG00000124813,ENSG00000131236,ENSG00000172765,ENSG00000136048,ENSG00000145819,ENSG00000167004,ENSG00000079841,ENSG00000105556,ENSG00000129116,ENSG00000154310,ENSG00000031003,ENSG00000140575,ENSG00000164430,ENSG00000114439,ENSG00000148935,ENSG00000077044,ENSG00000176390,ENSG00000141298,ENSG00000197256,ENSG00000127152,ENSG00000071537,ENSG00000180530,ENSG00000175899,ENSG00000173575,ENSG00000213892,ENSG00000159128,ENSG00000169862,ENSG00000110075,ENSG00000186635,ENSG00000169851,ENSG00000128849,ENSG00000138640,ENSG00000124942,ENSG00000072954,ENSG00000116350,ENSG00000198162,ENSG00000100116,ENSG00000198380,ENSG00000119314,ENSG00000110925,ENSG00000105176,ENSG00000170927,ENSG00000143376,ENSG00000197442,ENSG00000163947,ENSG00000072657,ENSG00000177463,ENSG00000124788,ENSG00000095139,ENSG00000148516,ENSG00000130429,ENSG00000138709,ENSG00000077782,ENSG00000216490,ENSG00000131873,ENSG00000050748,ENSG00000130956,ENSG00000196526,ENSG00000143768,ENSG00000213639,ENSG00000124126,ENSG00000164292,ENSG00000169398,ENSG00000013364,ENSG00000159216,ENSG00000106571,ENSG00000135090,ENSG00000168389,ENSG00000188158,ENSG00000130529,ENSG00000008294,ENSG00000181031,ENSG00000134369,ENSG00000102189,ENSG00000153317,ENSG00000105701,ENSG00000106049,ENSG00000157399,ENSG00000173040,ENSG00000105419,ENSG00000104671,ENSG00000100485,ENSG00000169902,ENSG00000054611,ENSG00000173068,ENSG00000167460,ENSG00000116514,ENSG00000133392,ENSG00000136478,ENSG00000115548,ENSG00000143970,ENSG00000196141,ENSG00000111490,ENSG00000086991,ENSG00000099139,ENSG00000115109,ENSG00000103196,ENSG00000100099,ENSG00000124006,ENSG00000135052,ENSG00000134815,ENSG00000048052,ENSG00000066468,ENSG00000118322,ENSG00000114554,ENSG00000153721,ENSG00000130208,ENSG00000168487,ENSG00000064666,ENSG00000080603,ENSG00000169359,ENSG00000110395,ENSG00000169946,ENSG00000110880,ENSG00000184640,ENSG00000025796,ENSG00000058091,ENSG00000136237,ENSG00000110693,ENSG00000107957,ENSG00000134909,ENSG00000155660,ENSG00000111087
cellular_component	cell part	190	87.55760369			GO:0044464	ENSG00000124164,ENSG00000116833,ENSG00000090376,ENSG00000129515,ENSG00000157483,ENSG00000135525,ENSG00000148498,ENSG00000124571,ENSG00000151693,ENSG00000147251,ENSG00000067057,ENSG00000067955,ENSG00000111269,ENSG00000105329,ENSG00000198879,ENSG00000160087,ENSG00000033867,ENSG00000090861,ENSG00000160094,ENSG00000139514,ENSG00000115221,ENSG00000138119,ENSG00000166501,ENSG00000129566,ENSG00000157107,ENSG00000162482,ENSG00000197102,ENSG00000154930,ENSG00000159692,ENSG00000143797,ENSG00000138434,ENSG00000038382,ENSG00000169180,ENSG00000174238,ENSG00000005844,ENSG00000156011,ENSG00000112394,ENSG00000235568,ENSG00000171105,ENSG00000106070,ENSG00000118961,ENSG00000073803,ENSG00000138674,ENSG00000141298,ENSG00000176390,ENSG00000197256,ENSG00000167601,ENSG00000077044,ENSG00000175899,ENSG00000071537,ENSG00000127152,ENSG00000180530,ENSG00000101187,ENSG00000159128,ENSG00000173575,ENSG00000213892,ENSG00000124942,ENSG00000072954,ENSG00000186635,ENSG00000169851,ENSG00000138640,ENSG00000128849,ENSG00000175497,ENSG00000116350,ENSG00000169862,ENSG00000110075,ENSG00000143811,ENSG00000138798,ENSG00000181826,ENSG00000145819,ENSG00000079841,ENSG00000167004,ENSG00000091542,ENSG00000198604,ENSG00000136048,ENSG00000172765,ENSG00000131236,ENSG00000124813,ENSG00000164073,ENSG00000129116,ENSG00000105556,ENSG00000171517,ENSG00000164430,ENSG00000148935,ENSG00000114439,ENSG00000140575,ENSG00000169896,ENSG00000154310,ENSG00000031003,ENSG00000169398,ENSG00000164292,ENSG00000124126,ENSG00000099282,ENSG00000196526,ENSG00000143768,ENSG00000213639,ENSG00000146555,ENSG00000100068,ENSG00000159216,ENSG00000013364,ENSG00000135090,ENSG00000130529,ENSG00000188158,ENSG00000168389,ENSG00000106571,ENSG00000102189,ENSG00000008294,ENSG00000134369,ENSG00000181031,ENSG00000198380,ENSG00000119314,ENSG00000198162,ENSG00000100116,ENSG00000197442,ENSG00000143376,ENSG00000163947,ENSG00000170927,ENSG00000110925,ENSG00000105176,ENSG00000130429,ENSG00000131873,ENSG00000216490,ENSG00000077782,ENSG00000138709,ENSG00000177463,ENSG00000072657,ENSG00000148516,ENSG00000124788,ENSG00000095139,ENSG00000130956,ENSG00000050748,ENSG00000100077,ENSG00000119686,ENSG00000066468,ENSG00000048052,ENSG00000118322,ENSG00000114554,ENSG00000153721,ENSG00000130208,ENSG00000124006,ENSG00000139540,ENSG00000100099,ENSG00000134815,ENSG00000135052,ENSG00000169946,ENSG00000110880,ENSG00000169359,ENSG00000110395,ENSG00000057019,ENSG00000064666,ENSG00000168487,ENSG00000080603,ENSG00000107957,ENSG00000110693,ENSG00000144426,ENSG00000184640,ENSG00000025796,ENSG00000058091,ENSG00000156886,ENSG00000136237,ENSG00000080493,ENSG00000134909,ENSG00000150938,ENSG00000155660,ENSG00000111087,ENSG00000106049,ENSG00000173040,ENSG00000157399,ENSG00000153317,ENSG00000105701,ENSG00000100485,ENSG00000104671,ENSG00000173068,ENSG00000054611,ENSG00000169902,ENSG00000105419,ENSG00000133392,ENSG00000115548,ENSG00000136478,ENSG00000167460,ENSG00000116514,ENSG00000086991,ENSG00000111490,ENSG00000136040,ENSG00000103196,ENSG00000115109,ENSG00000099139,ENSG00000196141,ENSG00000143970,ENSG00000076356

cellular_component	cell	190	87.55760369		GO:0005623	ENSG00000160094,ENSG00000139514,ENSG00000105329,ENSG00000198879,ENSG00000033867,ENSG00000160087,ENSG00000090861,ENSG00000148498,ENSG00000124571,ENSG00000151693,ENSG00000147251,ENSG00000067057,ENSG00000067955,ENSG00000111269,ENSG00000124164,ENSG00000116833,ENSG00000090376,ENSG00000129515,ENSG00000135525,ENSG00000157483,ENSG00000112394,ENSG00000156011,ENSG00000235568,ENSG00000171105,ENSG00000106070,ENSG00000118961,ENSG00000138674,ENSG00000073803,ENSG00000138434,ENSG00000038382,ENSG00000169180,ENSG00000174238,ENSG00000005844,ENSG00000157107,ENSG00000162482,ENSG00000197102,ENSG00000154930,ENSG00000159692,ENSG00000143797,ENSG00000138119,ENSG00000115221,ENSG00000166501,ENSG00000129566,ENSG00000124942,ENSG00000072954,ENSG00000186635,ENSG00000169851,ENSG00000138640,ENSG00000128849,ENSG00000175497,ENSG00000116350,ENSG00000169862,ENSG00000110075,ENSG00000101187,ENSG00000159128,ENSG00000173575,ENSG00000213892,ENSG00000175899,ENSG00000071537,ENSG00000127152,ENSG00000180530,ENSG00000141298,ENSG00000176390,ENSG00000197256,ENSG00000167601,ENSG00000077044,ENSG00000114439,ENSG00000164430,ENSG00000148935,ENSG00000140575,ENSG00000169896,ENSG00000154310,ENSG00000031003,ENSG00000129116,ENSG00000105556,ENSG00000171517,ENSG00000145819,ENSG00000167004,ENSG00000079841,ENSG00000198604,ENSG00000091542,ENSG00000172765,ENSG00000131236,ENSG00000136048,ENSG00000164073,ENSG00000124813,ENSG00000143811,ENSG00000138798,ENSG00000181826,ENSG00000102189,ENSG00000008294,ENSG00000134369,ENSG00000181031,ENSG00000135090,ENSG00000130529,ENSG00000188158,ENSG00000168389,ENSG00000106571,ENSG00000146555,ENSG00000100068,ENSG00000159216,ENSG00000013364,ENSG00000164292,ENSG00000169398,ENSG00000124126,ENSG00000099282,ENSG00000196526,ENSG00000143768,ENSG00000213639,ENSG00000130956,ENSG00000050748,ENSG00000100077,ENSG00000119686,ENSG00000130429,ENSG00000077782,ENSG00000216490,ENSG00000131873,ENSG00000138709,ENSG00000177463,ENSG00000072657,ENSG00000148516,ENSG00000095139,ENSG00000124788,ENSG00000197442,ENSG00000143376,ENSG00000163947,ENSG00000170927,ENSG00000110925,ENSG00000105176,ENSG00000198380,ENSG00000119314,ENSG00000198162,ENSG00000100116,ENSG00000080493,ENSG00000134909,ENSG00000155660,ENSG00000111087,ENSG00000150938,ENSG00000107957,ENSG00000110693,ENSG00000144426,ENSG00000025796,ENSG00000184640,ENSG00000058091,ENSG00000156886,ENSG00000136237,ENSG00000110880,ENSG00000169946,ENSG00000110395,ENSG00000169359,ENSG00000057019,ENSG00000064666,ENSG00000168487,ENSG00000080603,ENSG00000048052,ENSG00000066468,ENSG00000153721,ENSG00000114554,ENSG00000118322,ENSG00000130208,ENSG00000124006,ENSG00000139540,ENSG00000100099,ENSG00000134815,ENSG00000135052,ENSG00000086991,ENSG00000111490,ENSG00000136040,ENSG00000103196,ENSG00000115109,ENSG00000099139,ENSG00000196141,ENSG00000143970,ENSG00000076356,ENSG00000133392,ENSG00000115548,ENSG00000136478,ENSG00000167460,ENSG00000116514,ENSG00000100485,ENSG00000104671,ENSG00000054611,ENSG00000173068,ENSG00000169902,ENSG00000105419,ENSG00000106049,ENSG00000157399,ENSG00000173040,ENSG00000153317,ENSG00000105701
cellular_component	cellular_component	210	96.77419355		GO:0005575	ENSG00000219073,ENSG00000129566,ENSG00000115221,ENSG00000138119,ENSG00000166501,ENSG00000159692,ENSG00000143797,ENSG00000157107,ENSG00000162482,ENSG00000197102,ENSG00000154930,ENSG00000174238,ENSG00000005844,ENSG00000138434,ENSG00000038382,ENSG00000169180,ENSG00000118961,ENSG00000106070,ENSG00000073803,ENSG00000138674,ENSG00000156011,ENSG00000112394,ENSG00000235568,ENSG00000171105,ENSG00000243480,ENSG00000129515,ENSG00000157483,ENSG00000135525,ENSG00000124164,ENSG00000116833,ENSG00000090376,ENSG00000147251,ENSG00000067057,ENSG00000067955,ENSG00000111269,ENSG00000124571,ENSG00000148498,ENSG00000187021,ENSG00000151693,ENSG00000160087,ENSG00000198879,ENSG00000033867,ENSG00000090861,ENSG00000105329,ENSG00000139514,ENSG00000160094,ENSG00000106771,ENSG00000146433,ENSG00000181826,ENSG00000143811,ENSG00000138798,ENSG00000174939,ENSG00000198604,ENSG00000091542,ENSG00000091704,ENSG00000172765,ENSG00000136048,ENSG00000131236,ENSG00000164073,ENSG00000124813,ENSG00000145819,ENSG00000167004,ENSG00000079841,ENSG00000105556,ENSG00000171517,ENSG00000129116,ENSG00000169896,ENSG00000154310,ENSG00000031003,ENSG00000153002,ENSG00000164430,ENSG00000114439,ENSG00000148935,ENSG00000140575,ENSG00000167601,ENSG00000077044,ENSG00000141298,ENSG00000176390,ENSG00000197256,ENSG00000140470,ENSG00000071537,ENSG00000127152,ENSG00000180530,ENSG00000240038,ENSG00000175899,ENSG00000173575,ENSG00000213892,ENSG00000101187,ENSG00000159128,ENSG00000169862,ENSG00000110075,ENSG00000124942,ENSG00000072954,ENSG00000186635,ENSG00000169851,ENSG00000128849,ENSG00000138640,ENSG00000175497,ENSG00000116350,ENSG00000142615,ENSG00000198162,ENSG00000100116,ENSG00000198380,ENSG00000119314,ENSG00000110925,ENSG00000075223,ENSG00000105176,ENSG00000163947,ENSG00000197442,ENSG00000143376,ENSG00000170927,ENSG00000177463,ENSG00000072657,ENSG00000148516,ENSG00000124788,ENSG00000095139,ENSG00000130429,ENSG00000216490,ENSG00000131873,ENSG00000077782,ENSG00000138709,ENSG00000119686,ENSG00000050748,ENSG00000130956,ENSG00000100077,ENSG00000099282,ENSG00000196526,ENSG00000143768,ENSG00000213639,ENSG00000169398,ENSG00000164292,ENSG00000124126,ENSG00000152078,ENSG00000100068,ENSG00000159216,ENSG00000013364,ENSG00000146555,ENSG00000106571,ENSG00000175535,ENSG00000215704,ENSG00000135090,ENSG00000130529,ENSG00000188158,ENSG00000168389,ENSG00000008294,ENSG00000142789,ENSG00000134369,ENSG00000181031,ENSG00000102189,ENSG00000153317,ENSG00000105701,ENSG00000106049,ENSG00000157399,ENSG00000173040,ENSG00000105419,ENSG00000100485,ENSG00000104671,ENSG00000054611,ENSG00000173068,ENSG00000169902,ENSG00000167460,ENSG00000116514,ENSG00000133392,ENSG00000136478,ENSG00000115548,ENSG00000077942,ENSG00000196141,ENSG00000143970,ENSG00000076356,ENSG00000111490,ENSG00000086991,ENSG00000136040,ENSG00000115109,ENSG00000103196,ENSG00000099139,ENSG00000139540,ENSG00000124006,ENSG00000100099,ENSG00000134815,ENSG00000135052,ENSG00000048052,ENSG00000066468,ENSG00000114554,ENSG00000153721,ENSG00000118322,ENSG00000130208,ENSG00000064666,ENSG00000057019,ENSG00000168487,ENSG00000080603,ENSG00000169946,ENSG00000110880,ENSG00000110395,ENSG00000169359,ENSG00000025796,ENSG00000184640,ENSG00000058091,ENSG00000156886,ENSG00000136237,ENSG00000103534,ENSG00000107957,ENSG00000110693,ENSG00000100731,ENSG00000144426,ENSG00000134909,ENSG00000075420,ENSG00000155660,ENSG00000150938,ENSG00000111087,ENSG00000080493
biological_process	regulation of metabolic process	100	46.08294931		GO:0019222	ENSG00000156011,ENSG00000130956,ENSG00000050748,ENSG00000235568,ENSG00000171105,ENSG00000106070,ENSG00000073803,ENSG00000038382,ENSG00000077782,ENSG00000216490,ENSG00000177463,ENSG00000148516,ENSG00000124788,ENSG00000197442,ENSG00000163947,ENSG00000170927,ENSG00000110925,ENSG00000159692,ENSG00000105176,ENSG00000198380,ENSG00000119314,ENSG00000166501,ENSG00000160094,ENSG00000008294,ENSG00000135090,ENSG00000105329,ENSG00000198879,ENSG00000106571,ENSG00000175535,ENSG00000148498,ENSG00000124571,ENSG00000151693,ENSG00000067955,ENSG00000159216,ENSG00000111269,ENSG00000013364,ENSG00000169398,ENSG00000116833,ENSG00000124164,ENSG00000124126,ENSG00000090376,ENSG00000129515,ENSG00000213639,ENSG00000143768,ENSG00000086991,ENSG00000114439,ENSG00000115109,ENSG00000140575,ENSG00000077942,ENSG00000154310,ENSG00000143970,ENSG00000031003,ENSG00000115548,ENSG00000105556,ENSG00000116514,ENSG00000171517,ENSG00000100485,ENSG00000145819,ENSG00000173068,ENSG00000079841,ENSG00000198604,ENSG00000131236,ENSG00000136048,ENSG00000124813,ENSG00000138798,ENSG00000105701,ENSG00000124942,ENSG00000138640,ENSG00000186635,ENSG00000116350,ENSG00000134909,ENSG00000169862,ENSG00000150938,ENSG00000110075,ENSG00000111087,ENSG00000107957,ENSG00000110693,ENSG00000173575,ENSG00000136237,ENSG00000169946,ENSG00000110880,ENSG00000169359,ENSG00000110395,ENSG00000175899,ENSG00000064666,ENSG00000168487,ENSG00000071537,ENSG00000127152,ENSG00000080603,ENSG00000180530,ENSG00000066468,ENSG00000048052,ENSG00000176390,ENSG00000197256,ENSG00000153721,ENSG00000130208,ENSG00000139540,ENSG00000100099,ENSG00000134815,ENSG00000135052

biological_process	cellular response to stimulus	106	48.84792627		GO:0051716	ENSG00000105701,ENSG00000173040,ENSG00000138798,ENSG00000143811,ENSG00000105419,ENSG00000124813,ENSG00000131236,ENSG00000145819,ENSG00000100485,ENSG00000167004,ENSG00000171517,ENSG00000115548,ENSG00000136478,ENSG00000154310,ENSG00000143970,ENSG00000169896,ENSG00000077942,ENSG00000076356,ENSG00000031003,ENSG00000136040,ENSG00000086991,ENSG00000140575,ENSG00000115109,ENSG00000164430,ENSG00000167601,ENSG00000139540,ENSG00000077044,ENSG00000176390,ENSG00000048052,ENSG00000066468,ENSG00000153721,ENSG00000197256,ENSG00000114554,ENSG00000071537,ENSG00000168487,ENSG00000127152,ENSG00000057019,ENSG00000064666,ENSG00000180530,ENSG00000169359,ENSG00000110395,ENSG00000110880,ENSG00000175899,ENSG00000156886,ENSG00000058091,ENSG00000136237,ENSG00000173575,ENSG00000110693,ENSG00000159128,ENSG00000134909,ENSG00000169862,ENSG00000155660,ENSG00000150938,ENSG00000111087,ENSG00000138640,ENSG00000186635,ENSG00000138119,ENSG00000115221,ENSG00000166501,ENSG00000075223,ENSG00000105176,ENSG00000170927,ENSG00000197442,ENSG00000163947,ENSG00000143376,ENSG00000162482,ENSG00000072657,ENSG00000177463,ENSG00000005844,ENSG00000148516,ENSG00000130429,ENSG00000038382,ENSG00000131873,ENSG00000216490,ENSG00000077782,ENSG00000073803,ENSG00000106070,ENSG00000235568,ENSG00000156011,ENSG00000130956,ENSG00000050748,ENSG00000171105,ENSG00000100077,ENSG00000196526,ENSG00000099282,ENSG00000157483,ENSG00000143768,ENSG00000129515,ENSG00000124164,ENSG00000116833,ENSG00000124126,ENSG00000169398,ENSG00000164292,ENSG00000090376,ENSG00000100068,ENSG00000147251,ENSG00000013364,ENSG00000111269,ENSG00000148498,ENSG00000106571,ENSG00000090861,ENSG00000135090,ENSG00000105329,ENSG00000130529,ENSG00000008294,ENSG00000181031
biological_process	macromolecule metabolic process	109	50.23041475		GO:0043170	ENSG00000173575,ENSG00000058091,ENSG00000110693,ENSG00000111087,ENSG00000155660,ENSG00000169862,ENSG00000175497,ENSG00000116350,ENSG00000142615,ENSG00000124942,ENSG00000186635,ENSG00000134815,ENSG00000139540,ENSG00000167601,ENSG00000100099,ENSG00000197256,ENSG00000153721,ENSG00000130208,ENSG00000066468,ENSG00000048052,ENSG00000141298,ENSG00000176390,ENSG00000080603,ENSG00000180530,ENSG00000064666,ENSG00000140470,ENSG00000168487,ENSG00000071537,ENSG00000127152,ENSG00000175899,ENSG00000169946,ENSG00000110880,ENSG00000169359,ENSG00000110395,ENSG00000116514,ENSG00000171517,ENSG00000105556,ENSG00000115548,ENSG00000077942,ENSG00000143970,ENSG00000154310,ENSG00000115109,ENSG00000153002,ENSG00000114439,ENSG00000099139,ENSG00000140575,ENSG00000086991,ENSG00000105701,ENSG00000157399,ENSG00000138798,ENSG00000091704,ENSG00000124813,ENSG00000174939,ENSG00000091542,ENSG00000198604,ENSG00000173068,ENSG00000167004,ENSG00000079841,ENSG00000169902,ENSG00000090861,ENSG00000215704,ENSG00000198879,ENSG00000160087,ENSG00000106571,ENSG00000130529,ENSG00000105329,ENSG00000135090,ENSG00000142789,ENSG00000008294,ENSG00000160094,ENSG00000129515,ENSG00000143768,ENSG00000213639,ENSG00000099282,ENSG00000090376,ENSG00000169398,ENSG00000116833,ENSG00000124164,ENSG00000111269,ENSG00000159216,ENSG00000013364,ENSG00000067955,ENSG00000148498,ENSG00000124571,ENSG00000148516,ENSG00000124788,ENSG00000177463,ENSG00000072657,ENSG00000216490,ENSG00000077782,ENSG00000131873,ENSG00000038382,ENSG00000073803,ENSG00000171105,ENSG00000100077,ENSG00000130956,ENSG00000050748,ENSG00000235568,ENSG00000129566,ENSG00000219073,ENSG00000198162,ENSG00000119314,ENSG00000166501,ENSG00000198380,ENSG00000105176,ENSG00000110925,ENSG00000159692,ENSG00000197442,ENSG00000170927
biological_process	response to stimulus	120	55.29953917		GO:0050896	ENSG00000186635,ENSG00000138640,ENSG00000116350,ENSG00000134909,ENSG00000169862,ENSG00000150938,ENSG00000155660,ENSG00000111087,ENSG00000110693,ENSG00000159128,ENSG00000058091,ENSG00000156886,ENSG00000173575,ENSG00000136237,ENSG00000110880,ENSG00000169946,ENSG00000110395,ENSG00000169359,ENSG00000175899,ENSG00000057019,ENSG00000064666,ENSG00000071537,ENSG00000168487,ENSG00000127152,ENSG00000180530,ENSG00000048052,ENSG00000066468,ENSG00000176390,ENSG00000197256,ENSG00000153721,ENSG00000114554,ENSG00000167601,ENSG00000139540,ENSG00000100099,ENSG00000077044,ENSG00000086991,ENSG00000136040,ENSG00000164430,ENSG00000115109,ENSG00000140575,ENSG00000169896,ENSG00000077942,ENSG00000154310,ENSG00000143970,ENSG00000031003,ENSG00000076356,ENSG00000136478,ENSG00000115548,ENSG00000116514,ENSG00000171517,ENSG00000100485,ENSG00000145819,ENSG00000079841,ENSG00000167004,ENSG00000169902,ENSG00000091542,ENSG00000131236,ENSG00000124813,ENSG00000105419,ENSG00000143811,ENSG00000138798,ENSG00000173040,ENSG00000105701,ENSG00000008294,ENSG00000181031,ENSG00000105329,ENSG00000135090,ENSG00000130529,ENSG00000160087,ENSG00000106571,ENSG00000090861,ENSG00000148498,ENSG00000146555,ENSG00000147251,ENSG00000100068,ENSG00000111269,ENSG00000013364,ENSG00000169398,ENSG00000164292,ENSG00000124126,ENSG00000124164,ENSG00000116833,ENSG00000090376,ENSG00000099282,ENSG00000196526,ENSG00000129515,ENSG00000157483,ENSG00000213639,ENSG00000135525,ENSG00000143768,ENSG00000156011,ENSG00000050748,ENSG00000130956,ENSG00000235568,ENSG00000171105,ENSG00000100077,ENSG00000106070,ENSG00000073803,ENSG00000138674,ENSG00000038382,ENSG00000130429,ENSG00000216490,ENSG00000077782,ENSG00000131873,ENSG00000177463,ENSG00000072657,ENSG00000148516,ENSG00000005844,ENSG00000095139,ENSG00000197442,ENSG00000163947,ENSG00000162482,ENSG00000143376,ENSG00000170927,ENSG00000075223,ENSG00000266200,ENSG00000105176,ENSG00000138119,ENSG00000115221,ENSG00000166501
biological_process	cellular metabolic process	121	55.76036866		GO:0044237	ENSG00000198162,ENSG00000129566,ENSG00000100116,ENSG00000198380,ENSG00000166501,ENSG00000119314,ENSG00000143797,ENSG00000110925,ENSG00000159692,ENSG00000105176,ENSG00000170927,ENSG00000154930,ENSG00000197442,ENSG00000162482,ENSG00000163947,ENSG00000072657,ENSG00000177463,ENSG00000124788,ENSG00000148516,ENSG00000038382,ENSG00000077782,ENSG00000131873,ENSG00000073803,ENSG00000106070,ENSG00000235568,ENSG00000156011,ENSG00000130956,ENSG00000050748,ENSG00000171105,ENSG00000100077,ENSG00000213639,ENSG00000143768,ENSG00000129515,ENSG00000124164,ENSG00000124126,ENSG00000116833,ENSG00000169398,ENSG00000090376,ENSG00000067955,ENSG00000067057,ENSG00000013364,ENSG00000111269,ENSG00000159216,ENSG00000148498,ENSG00000151693,ENSG00000106571,ENSG00000198879,ENSG00000160087,ENSG00000175535,ENSG00000090861,ENSG00000135090,ENSG00000105329,ENSG00000130529,ENSG00000008294,ENSG00000170835,ENSG00000160094,ENSG00000105701,ENSG00000106049,ENSG00000138798,ENSG00000157399,ENSG00000143811,ENSG00000198604,ENSG00000091542,ENSG00000174939,ENSG00000124813,ENSG00000131236,ENSG00000136048,ENSG00000145819,ENSG00000100485,ENSG00000169902,ENSG00000167004,ENSG00000173068,ENSG00000105556,ENSG00000171517,ENSG00000116514,ENSG00000115548,ENSG00000136478,ENSG00000154310,ENSG00000143970,ENSG00000077942,ENSG00000031003,ENSG00000086991,ENSG00000099139,ENSG00000140575,ENSG00000114439,ENSG00000164430,ENSG00000167601,ENSG00000139540,ENSG00000077044,ENSG00000134815,ENSG00000176390,ENSG00000048052,ENSG00000141298,ENSG00000066468,ENSG00000130208,ENSG00000197256,ENSG00000153721,ENSG00000168487,ENSG00000071537,ENSG00000127152,ENSG00000180530,ENSG00000080603,ENSG00000110395,ENSG00000169359,ENSG00000110880,ENSG00000169946,ENSG00000175899,ENSG00000058091,ENSG00000136237,ENSG00000173575,ENSG00000110693,ENSG00000107957,ENSG00000169862,ENSG00000134909,ENSG00000155660,ENSG00000110075,ENSG00000111087,ENSG00000186635,ENSG00000138640,ENSG00000124942,ENSG00000116350

biological_process	primary metabolic process	130	59.9078341		GO:0044238	ENSG00000198162,ENSG00000100116,ENSG00000198380,ENSG00000119314,ENSG00000110925,ENSG00000105176,ENSG00000170927,ENSG00000163947,ENSG00000197442,ENSG00000072657,ENSG00000177463,ENSG00000124788,ENSG00000148516,ENSG00000131873,ENSG00000077782,ENSG00000050748,ENSG00000130956,ENSG00000100077,ENSG00000099282,ENSG00000213639,ENSG00000143768,ENSG00000124126,ENSG00000169398,ENSG00000013364,ENSG00000159216,ENSG00000106571,ENSG00000215704,ENSG00000175535,ENSG00000135090,ENSG00000130529,ENSG00000008294,ENSG00000142789,ENSG00000170835,ENSG00000105701,ENSG00000106049,ENSG00000157399,ENSG00000100485,ENSG00000169902,ENSG00000173068,ENSG00000116514,ENSG00000136478,ENSG00000115548,ENSG00000143970,ENSG00000077942,ENSG00000086991,ENSG00000099139,ENSG00000139540,ENSG00000135052,ENSG00000134815,ENSG00000066468,ENSG00000048052,ENSG00000153721,ENSG00000130208,ENSG00000168487,ENSG00000080603,ENSG00000110395,ENSG00000169359,ENSG00000169946,ENSG00000110880,ENSG00000058091,ENSG00000136237,ENSG00000110693,ENSG00000134909,ENSG00000111087,ENSG00000155660,ENSG00000219073,ENSG00000129566,ENSG00000166501,ENSG00000143797,ENSG00000159692,ENSG00000154930,ENSG00000174238,ENSG00000038382,ENSG00000073803,ENSG00000118961,ENSG00000235568,ENSG00000156011,ENSG00000171105,ENSG00000129515,ENSG00000243480,ENSG00000124164,ENSG00000116833,ENSG00000090376,ENSG00000067955,ENSG00000111269,ENSG00000148498,ENSG00000151693,ENSG00000187021,ENSG00000198879,ENSG00000160087,ENSG00000090861,ENSG00000105329,ENSG00000160094,ENSG00000138798,ENSG00000143811,ENSG00000091542,ENSG00000198604,ENSG00000174939,ENSG00000124813,ENSG00000091704,ENSG00000131236,ENSG00000145819,ENSG00000167004,ENSG00000105556,ENSG00000171517,ENSG00000154310,ENSG00000031003,ENSG00000140575,ENSG00000164430,ENSG00000153002,ENSG00000114439,ENSG00000167601,ENSG00000077044,ENSG00000176390,ENSG00000141298,ENSG00000197256,ENSG00000071537,ENSG00000127152,ENSG00000140470,ENSG00000180530,ENSG00000175899,ENSG00000240038,ENSG00000173575,ENSG00000169862,ENSG00000186635,ENSG00000138640,ENSG00000124942,ENSG00000142615,ENSG00000116350,ENSG00000175497
biological_process	regulation of cellular process	136	62.67281106		GO:0050794	ENSG00000173040,ENSG00000105701,ENSG00000153317,ENSG00000173068,ENSG00000054611,ENSG00000100485,ENSG00000105419,ENSG00000115548,ENSG00000136478,ENSG00000116514,ENSG00000115109,ENSG00000136040,ENSG00000086991,ENSG00000111490,ENSG00000076356,ENSG00000143970,ENSG00000077942,ENSG00000130208,ENSG00000153721,ENSG00000114554,ENSG00000066468,ENSG00000048052,ENSG00000134815,ENSG00000124006,ENSG00000139540,ENSG00000169359,ENSG00000110395,ENSG00000110880,ENSG00000169946,ENSG00000080603,ENSG00000168487,ENSG00000064666,ENSG00000057019,ENSG00000110693,ENSG00000136237,ENSG00000156886,ENSG00000058091,ENSG00000155660,ENSG00000111087,ENSG00000150938,ENSG00000134909,ENSG00000119314,ENSG00000170927,ENSG00000197442,ENSG00000163947,ENSG00000143376,ENSG00000105176,ENSG00000075223,ENSG00000110925,ENSG00000216490,ENSG00000131873,ENSG00000077782,ENSG00000130429,ENSG00000124788,ENSG00000148516,ENSG00000072657,ENSG00000177463,ENSG00000100077,ENSG00000130956,ENSG00000050748,ENSG00000124126,ENSG00000164292,ENSG00000169398,ENSG00000143768,ENSG00000213639,ENSG00000196526,ENSG00000099282,ENSG00000146555,ENSG00000013364,ENSG00000159216,ENSG00000100068,ENSG00000130529,ENSG00000135090,ENSG00000106571,ENSG00000181031,ENSG00000008294,ENSG00000138798,ENSG00000079841,ENSG00000167004,ENSG00000145819,ENSG00000124813,ENSG00000136048,ENSG00000131236,ENSG00000198604,ENSG00000171517,ENSG00000105556,ENSG00000140575,ENSG00000148935,ENSG00000114439,ENSG00000031003,ENSG00000154310,ENSG00000169896,ENSG00000197256,ENSG00000176390,ENSG00000141298,ENSG00000077044,ENSG00000167601,ENSG00000175899,ENSG00000180530,ENSG00000071537,ENSG00000127152,ENSG00000159128,ENSG00000173575,ENSG00000175497,ENSG00000116350,ENSG00000138640,ENSG00000186635,ENSG00000124942,ENSG00000110075,ENSG00000169862,ENSG00000166501,ENSG00000138119,ENSG00000115221,ENSG00000159692,ENSG00000038382,ENSG00000005844,ENSG00000171105,ENSG00000235568,ENSG00000156011,ENSG00000073803,ENSG00000106070,ENSG00000090376,ENSG00000124164,ENSG00000116833,ENSG00000157483,ENSG00000129515,ENSG00000151693,ENSG00000124571,ENSG00000148498,ENSG00000111269,ENSG00000067955,ENSG00000147251,ENSG00000105329,ENSG00000090861,ENSG00000198879,ENSG00000160094
biological_process	organic substance metabolic process	138	63.59447005		GO:0071704	ENSG00000159692,ENSG00000143797,ENSG00000162482,ENSG00000154930,ENSG00000219073,ENSG00000129566,ENSG00000166501,ENSG00000118961,ENSG00000073803,ENSG00000156011,ENSG00000235568,ENSG00000171105,ENSG00000174238,ENSG00000038382,ENSG00000067057,ENSG00000067955,ENSG00000111269,ENSG00000124571,ENSG00000148498,ENSG00000187021,ENSG00000151693,ENSG00000129515,ENSG00000243480,ENSG00000124164,ENSG00000116833,ENSG00000090376,ENSG00000160094,ENSG00000198879,ENSG00000160087,ENSG00000090861,ENSG00000105329,ENSG00000174939,ENSG00000198604,ENSG00000091542,ENSG00000131236,ENSG00000091704,ENSG00000124813,ENSG00000145819,ENSG00000079841,ENSG00000167004,ENSG00000143811,ENSG00000138798,ENSG00000154310,ENSG00000031003,ENSG00000164430,ENSG00000153002,ENSG00000114439,ENSG00000140575,ENSG00000105556,ENSG00000171517,ENSG00000140470,ENSG00000127152,ENSG00000071537,ENSG00000180530,ENSG00000175899,ENSG00000240038,ENSG00000167601,ENSG00000077044,ENSG00000141298,ENSG00000176390,ENSG00000197256,ENSG00000169862,ENSG00000124942,ENSG00000138640,ENSG00000186635,ENSG00000175497,ENSG00000116350,ENSG00000142615,ENSG00000173575,ENSG00000110925,ENSG00000105176,ENSG00000163947,ENSG00000197442,ENSG00000170927,ENSG00000198162,ENSG00000100116,ENSG00000198380,ENSG00000119314,ENSG00000130956,ENSG00000050748,ENSG00000100077,ENSG00000177463,ENSG00000072657,ENSG00000148516,ENSG00000124788,ENSG00000216490,ENSG00000131873,ENSG00000077782,ENSG00000159216,ENSG00000013364,ENSG00000099282,ENSG00000143768,ENSG00000213639,ENSG00000169398,ENSG00000124126,ENSG00000008294,ENSG00000142789,ENSG00000170835,ENSG00000106571,ENSG00000175535,ENSG00000215704,ENSG00000135090,ENSG00000130529,ENSG00000100485,ENSG00000173068,ENSG00000169902,ENSG00000105701,ENSG00000106049,ENSG00000157399,ENSG00000077942,ENSG00000143970,ENSG00000086991,ENSG00000115109,ENSG00000099139,ENSG00000116514,ENSG00000115548,ENSG00000136478,ENSG00000064666,ENSG00000168487,ENSG00000080603,ENSG00000169946,ENSG00000110880,ENSG00000169359,ENSG00000110395,ENSG00000139540,ENSG00000100099,ENSG00000134815,ENSG00000135052,ENSG00000066468,ENSG00000048052,ENSG00000153721,ENSG00000130208,ENSG00000134909,ENSG00000155660,ENSG00000111087,ENSG00000058091,ENSG00000136237,ENSG00000110693

biological_process	regulation of biological process	143	65.89861751		GO:0050789	ENSG00000159692,ENSG00000115221,ENSG00000138119,ENSG00000166501,ENSG00000073803,ENSG00000106070,ENSG00000235568,ENSG00000156011,ENSG00000171105,ENSG00000005844,ENSG00000038382,ENSG00000067955,ENSG00000147251,ENSG00000111269,ENSG00000124571,ENSG00000148498,ENSG00000151693,ENSG00000157483,ENSG00000129515,ENSG00000124164,ENSG00000116833,ENSG00000090376,ENSG00000160094,ENSG00000198879,ENSG00000090861,ENSG00000105329,ENSG00000198604,ENSG00000091542,ENSG00000124813,ENSG00000131236,ENSG00000136048,ENSG00000145819,ENSG00000167004,ENSG00000079841,ENSG00000138798,ENSG00000154310,ENSG00000169896,ENSG00000031003,ENSG00000140575,ENSG00000164430,ENSG00000148935,ENSG00000114439,ENSG00000105556,ENSG00000171517,ENSG00000071537,ENSG00000127152,ENSG00000180530,ENSG00000175899,ENSG00000167601,ENSG00000077044,ENSG00000176390,ENSG00000141298,ENSG00000197256,ENSG00000169862,ENSG00000110075,ENSG00000186635,ENSG00000138640,ENSG00000124942,ENSG00000116350,ENSG00000175497,ENSG00000173575,ENSG00000159128,ENSG00000075223,ENSG00000110925,ENSG00000105176,ENSG00000170927,ENSG00000143376,ENSG00000197442,ENSG00000163947,ENSG00000198380,ENSG00000119314,ENSG00000130956,ENSG00000050748,ENSG00000100077,ENSG00000072657,ENSG00000177463,ENSG00000124788,ENSG00000148516,ENSG00000130429,ENSG00000077782,ENSG00000216490,ENSG00000131873,ENSG00000100068,ENSG00000013364,ENSG00000159216,ENSG00000146555,ENSG00000196526,ENSG00000099282,ENSG00000143768,ENSG00000213639,ENSG00000124126,ENSG00000164292,ENSG00000169398,ENSG00000008294,ENSG00000181031,ENSG00000106571,ENSG00000175535,ENSG00000135090,ENSG00000130529,ENSG00000105419,ENSG00000100485,ENSG00000054611,ENSG00000173068,ENSG00000153317,ENSG00000105701,ENSG00000173040,ENSG00000143970,ENSG00000077942,ENSG00000076356,ENSG00000136040,ENSG00000111490,ENSG00000086991,ENSG00000115109,ENSG00000116514,ENSG00000115548,ENSG00000136478,ENSG00000168487,ENSG00000064666,ENSG00000057019,ENSG00000080603,ENSG00000169359,ENSG00000110395,ENSG00000169946,ENSG00000110880,ENSG00000100099,ENSG00000139540,ENSG00000124006,ENSG00000135052,ENSG00000134815,ENSG00000048052,ENSG00000066468,ENSG00000153721,ENSG00000114554,ENSG00000130208,ENSG00000134909,ENSG00000155660,ENSG00000111087,ENSG00000150938,ENSG00000156886,ENSG00000058091,ENSG00000136237,ENSG00000110693,ENSG00000107957
biological_process	metabolic process	146	67.28110599		GO:0008152	ENSG00000105329,ENSG00000160087,ENSG00000198879,ENSG00000090861,ENSG00000160094,ENSG00000124164,ENSG00000116833,ENSG00000090376,ENSG00000157483,ENSG00000243480,ENSG00000129515,ENSG00000124571,ENSG00000148498,ENSG00000151693,ENSG00000187021,ENSG00000067955,ENSG00000067057,ENSG00000111269,ENSG00000038382,ENSG00000174238,ENSG00000235568,ENSG00000156011,ENSG00000171105,ENSG00000073803,ENSG00000106070,ENSG00000118961,ENSG00000166501,ENSG00000219073,ENSG00000129566,ENSG00000154930,ENSG00000162482,ENSG00000143797,ENSG00000159692,ENSG00000173575,ENSG00000138640,ENSG00000186635,ENSG00000124942,ENSG00000142615,ENSG00000175497,ENSG00000116350,ENSG00000169862,ENSG00000110075,ENSG00000176390,ENSG00000141298,ENSG00000197256,ENSG00000167601,ENSG00000077044,ENSG00000175899,ENSG00000240038,ENSG00000127152,ENSG00000071537,ENSG00000140470,ENSG00000180530,ENSG00000105556,ENSG00000171517,ENSG00000140575,ENSG00000153002,ENSG00000164430,ENSG00000114439,ENSG00000154310,ENSG00000031003,ENSG00000138798,ENSG00000143811,ENSG00000145819,ENSG00000079841,ENSG00000167004,ENSG00000091542,ENSG00000198604,ENSG00000174939,ENSG00000124813,ENSG00000131236,ENSG00000136048,ENSG00000091704,ENSG00000135090,ENSG00000130529,ENSG00000106571,ENSG00000175535,ENSG00000215704,ENSG00000170835,ENSG00000008294,ENSG00000142789,ENSG00000124126,ENSG00000169398,ENSG00000099282,ENSG00000213639,ENSG00000143768,ENSG00000013364,ENSG00000159216,ENSG00000131873,ENSG00000216490,ENSG00000077782,ENSG00000072657,ENSG00000177463,ENSG00000124788,ENSG00000148516,ENSG00000130956,ENSG00000050748,ENSG00000100077,ENSG00000258539,ENSG00000198380,ENSG00000119314,ENSG00000198162,ENSG00000100116,ENSG00000170927,ENSG00000163947,ENSG00000197442,ENSG00000110925,ENSG00000105176,ENSG00000110693,ENSG00000107957,ENSG00000058091,ENSG00000025796,ENSG00000136237,ENSG00000134909,ENSG00000111087,ENSG00000150938,ENSG00000155660,ENSG00000048052,ENSG00000066468,ENSG00000153721,ENSG00000130208,ENSG00000100099,ENSG00000139540,ENSG00000135052,ENSG00000134815,ENSG00000169359,ENSG00000110395,ENSG00000169946,ENSG00000110880,ENSG00000168487,ENSG00000064666,ENSG00000080603,ENSG00000115548,ENSG00000136478,ENSG00000116514,ENSG00000086991,ENSG00000099139,ENSG00000115109,ENSG00000143970,ENSG00000077942,ENSG00000106049,ENSG00000157399,ENSG00000105701,ENSG00000100485,ENSG00000169902,ENSG00000173068
biological_process	biological regulation	150	69.12442396		GO:0065007	ENSG00000181031,ENSG00000008294,ENSG00000130529,ENSG00000135090,ENSG00000175535,ENSG00000106571,ENSG00000146555,ENSG00000159216,ENSG00000013364,ENSG00000100068,ENSG00000169398,ENSG00000164292,ENSG00000124126,ENSG00000143768,ENSG00000213639,ENSG00000099282,ENSG00000196526,ENSG00000100077,ENSG00000130956,ENSG00000050748,ENSG00000077782,ENSG00000216490,ENSG00000131873,ENSG00000130429,ENSG00000148516,ENSG00000124788,ENSG00000177463,ENSG00000072657,ENSG00000197442,ENSG00000163947,ENSG00000143376,ENSG00000170927,ENSG00000105176,ENSG00000075223,ENSG00000110925,ENSG00000119314,ENSG00000198380,ENSG00000080493,ENSG00000155660,ENSG00000111087,ENSG00000150938,ENSG00000134909,ENSG00000107957,ENSG00000110693,ENSG00000136237,ENSG00000058091,ENSG00000156886,ENSG00000110880,ENSG00000169946,ENSG00000169359,ENSG00000110395,ENSG00000080603,ENSG00000057019,ENSG00000064666,ENSG00000168487,ENSG00000118322,ENSG00000114554,ENSG00000130208,ENSG00000153721,ENSG00000066468,ENSG00000048052,ENSG00000134815,ENSG00000135052,ENSG00000139540,ENSG00000124006,ENSG00000100099,ENSG00000115109,ENSG00000099139,ENSG00000086991,ENSG00000111490,ENSG00000136040,ENSG00000076356,ENSG00000077942,ENSG00000143970,ENSG00000115548,ENSG00000136478,ENSG00000116514,ENSG00000054611,ENSG00000173068,ENSG00000100485,ENSG00000105419,ENSG00000173040,ENSG00000105701,ENSG00000153317,ENSG00000160094,ENSG00000105329,ENSG00000090861,ENSG00000198879,ENSG00000033867,ENSG00000151693,ENSG00000148498,ENSG00000124571,ENSG00000111269,ENSG00000147251,ENSG00000067955,ENSG00000090376,ENSG00000116833,ENSG00000124164,ENSG00000129515,ENSG00000157483,ENSG00000171105,ENSG00000156011,ENSG00000112394,ENSG00000235568,ENSG00000106070,ENSG00000073803,ENSG00000038382,ENSG00000005844,ENSG00000159692,ENSG00000166501,ENSG00000115221,ENSG00000138119,ENSG00000129566,ENSG00000116350,ENSG00000175497,ENSG00000124942,ENSG00000138640,ENSG00000186635,ENSG00000110075,ENSG00000169862,ENSG00000101187,ENSG00000159128,ENSG00000173575,ENSG00000175899,ENSG00000180530,ENSG00000127152,ENSG00000071537,ENSG00000197256,ENSG00000141298,ENSG00000176390,ENSG00000077044,ENSG00000167601,ENSG00000164430,ENSG00000148935,ENSG00000114439,ENSG00000140575,ENSG00000031003,ENSG00000169896,ENSG00000154310,ENSG00000171517,ENSG00000105556,ENSG00000167004,ENSG00000079841,ENSG00000145819,ENSG00000131236,ENSG00000136048,ENSG00000124813,ENSG00000198604,ENSG00000091542,ENSG00000138798

biological_process	single-organism cellular process	161	74.19354839		GO:0044763	ENSG00000110075,ENSG00000169862,ENSG00000116350,ENSG00000175497,ENSG00000169851,ENSG00000186635,ENSG00000138640,ENSG00000072954,ENSG00000124942,ENSG00000173575,ENSG00000159128,ENSG00000180530,ENSG00000071537,ENSG00000127152,ENSG00000175899,ENSG00000077044,ENSG00000167601,ENSG00000197256,ENSG00000176390,ENSG00000141298,ENSG00000031003,ENSG00000154310,ENSG00000169896,ENSG00000140575,ENSG00000148935,ENSG00000164430,ENSG00000171517,ENSG00000129116,ENSG00000124813,ENSG00000164073,ENSG00000131236,ENSG00000136048,ENSG00000091542,ENSG00000198604,ENSG00000167004,ENSG00000079841,ENSG00000145819,ENSG00000138798,ENSG00000143811,ENSG00000090861,ENSG00000033867,ENSG00000105329,ENSG00000111269,ENSG00000067955,ENSG00000147251,ENSG00000148498,ENSG00000124571,ENSG00000157483,ENSG00000135525,ENSG00000129515,ENSG00000090376,ENSG00000116833,ENSG00000124164,ENSG00000138674,ENSG00000073803,ENSG00000106070,ENSG00000171105,ENSG00000235568,ENSG00000156011,ENSG00000112394,ENSG00000005844,ENSG00000169180,ENSG00000038382,ENSG00000159692,ENSG00000162482,ENSG00000197102,ENSG00000157107,ENSG00000129566,ENSG00000166501,ENSG00000115221,ENSG00000138119,ENSG00000150938,ENSG00000111087,ENSG00000155660,ENSG00000134909,ENSG00000080493,ENSG00000136237,ENSG00000156886,ENSG00000025796,ENSG00000184640,ENSG00000058091,ENSG00000110693,ENSG00000107957,ENSG00000080603,ENSG00000168487,ENSG00000057019,ENSG00000064666,ENSG00000169359,ENSG00000110395,ENSG00000110880,ENSG00000169946,ENSG00000135052,ENSG00000100099,ENSG00000124006,ENSG00000139540,ENSG00000118322,ENSG00000153721,ENSG00000114554,ENSG00000066468,ENSG00000048052,ENSG00000076356,ENSG00000143970,ENSG00000077942,ENSG00000099139,ENSG00000103196,ENSG00000115109,ENSG00000136040,ENSG00000111490,ENSG00000086991,ENSG00000167460,ENSG00000136478,ENSG00000115548,ENSG00000133392,ENSG00000105419,ENSG00000054611,ENSG00000104671,ENSG00000100485,ENSG00000105701,ENSG00000153317,ENSG00000173040,ENSG00000181031,ENSG00000134369,ENSG00000008294,ENSG00000102189,ENSG00000106571,ENSG00000188158,ENSG00000168389,ENSG00000130529,ENSG00000135090,ENSG00000013364,ENSG00000159216,ENSG00000100068,ENSG00000146555,ENSG00000213639,ENSG00000143768,ENSG00000196526,ENSG00000099282,ENSG00000124126,ENSG00000164292,ENSG00000169398,ENSG00000119686,ENSG00000100077,ENSG00000050748,ENSG00000130956,ENSG00000095139,ENSG00000124788,ENSG00000148516,ENSG00000072657,ENSG00000177463,ENSG00000131873,ENSG00000216490,ENSG00000077782,ENSG00000130429,ENSG00000105176,ENSG00000110925,ENSG00000075223,ENSG00000170927,ENSG00000197442,ENSG00000163947,ENSG00000143376,ENSG00000119314
biological_process	single-organism process	177	81.56682028		GO:0044699	ENSG00000124164,ENSG00000116833,ENSG00000090376,ENSG00000157483,ENSG00000135525,ENSG00000243480,ENSG00000129515,ENSG00000124571,ENSG00000148498,ENSG00000067955,ENSG00000147251,ENSG00000111269,ENSG00000105329,ENSG00000033867,ENSG00000090861,ENSG00000139514,ENSG00000138119,ENSG00000115221,ENSG00000166501,ENSG00000129566,ENSG00000157107,ENSG00000162482,ENSG00000197102,ENSG00000159692,ENSG00000038382,ENSG00000169180,ENSG00000174238,ENSG00000005844,ENSG00000235568,ENSG00000156011,ENSG00000112394,ENSG00000171105,ENSG00000073803,ENSG00000138674,ENSG00000106070,ENSG00000176390,ENSG00000141298,ENSG00000197256,ENSG00000167601,ENSG00000077044,ENSG00000240038,ENSG00000175899,ENSG00000127152,ENSG00000071537,ENSG00000180530,ENSG00000159128,ENSG00000101187,ENSG00000173575,ENSG00000213892,ENSG00000186635,ENSG00000169851,ENSG00000138640,ENSG00000072954,ENSG00000124942,ENSG00000116350,ENSG00000175497,ENSG00000169862,ENSG00000110075,ENSG00000138798,ENSG00000143811,ENSG00000145819,ENSG00000079841,ENSG00000167004,ENSG00000091542,ENSG00000198604,ENSG00000124813,ENSG00000164073,ENSG00000131236,ENSG00000136048,ENSG00000129116,ENSG00000171517,ENSG00000140575,ENSG00000148935,ENSG00000164430,ENSG00000114439,ENSG00000154310,ENSG00000169896,ENSG00000031003,ENSG00000124126,ENSG00000164292,ENSG00000169398,ENSG00000196526,ENSG00000099282,ENSG00000213639,ENSG00000143768,ENSG00000146555,ENSG00000100068,ENSG00000013364,ENSG00000159216,ENSG00000135090,ENSG00000168389,ENSG00000188158,ENSG00000130529,ENSG00000106571,ENSG00000175535,ENSG00000102189,ENSG00000008294,ENSG00000181031,ENSG00000142789,ENSG00000134369,ENSG00000198380,ENSG00000119314,ENSG00000198162,ENSG00000170927,ENSG00000197442,ENSG00000143376,ENSG00000163947,ENSG00000266200,ENSG00000110925,ENSG00000075223,ENSG00000105176,ENSG00000130429,ENSG00000131873,ENSG00000216490,ENSG00000077782,ENSG00000072657,ENSG00000177463,ENSG00000124788,ENSG00000095139,ENSG00000148516,ENSG00000130956,ENSG00000050748,ENSG00000100077,ENSG00000119686,ENSG00000066468,ENSG00000048052,ENSG00000130208,ENSG00000114554,ENSG00000153721,ENSG00000118322,ENSG00000100099,ENSG00000139540,ENSG00000124006,ENSG00000135052,ENSG00000169359,ENSG00000110395,ENSG00000110880,ENSG00000169946,ENSG00000168487,ENSG00000064666,ENSG00000057019,ENSG00000080603,ENSG00000110693,ENSG00000107957,ENSG00000156886,ENSG00000184640,ENSG00000025796,ENSG00000058091,ENSG00000103534,ENSG00000136237,ENSG00000080493,ENSG00000134909,ENSG00000111087,ENSG00000150938,ENSG00000155660,ENSG00000157399,ENSG00000173040,ENSG00000153317,ENSG00000105701,ENSG00000104671,ENSG00000100485,ENSG00000173068,ENSG00000054611,ENSG00000105419,ENSG00000133392,ENSG00000115548,ENSG00000136478,ENSG00000167460,ENSG00000136040,ENSG00000086991,ENSG00000111490,ENSG00000099139,ENSG00000103196,ENSG00000115109,ENSG00000143970,ENSG00000077942,ENSG00000076356

biological_process	cellular process	183	84.33179724		GO:0009987	ENSG00000127152,ENSG00000071537,ENSG00000180530,ENSG00000175899,ENSG00000167601,ENSG00000077044,ENSG00000141298,ENSG00000176390,ENSG00000197256,ENSG00000169862,ENSG00000110075,ENSG00000124942,ENSG00000072954,ENSG00000186635,ENSG00000169851,ENSG00000138640,ENSG00000116350,ENSG00000175497,ENSG00000173575,ENSG00000159128,ENSG00000174939,ENSG00000198604,ENSG00000091542,ENSG00000136048,ENSG00000131236,ENSG00000164073,ENSG00000124813,ENSG00000145819,ENSG00000167004,ENSG00000079841,ENSG00000143811,ENSG00000138798,ENSG00000169896,ENSG00000154310,ENSG00000031003,ENSG00000148935,ENSG00000164430,ENSG00000114439,ENSG00000140575,ENSG00000105556,ENSG00000171517,ENSG00000129116,ENSG00000147251,ENSG00000067057,ENSG00000067955,ENSG00000111269,ENSG00000148498,ENSG00000124571,ENSG00000151693,ENSG00000129515,ENSG00000135525,ENSG00000157483,ENSG00000124164,ENSG00000116833,ENSG00000090376,ENSG00000160094,ENSG00000198879,ENSG00000033867,ENSG00000160087,ENSG00000090861,ENSG00000105329,ENSG00000159692,ENSG00000143797,ENSG00000157107,ENSG00000197102,ENSG00000162482,ENSG00000154930,ENSG00000129566,ENSG00000115221,ENSG00000138119,ENSG00000166501,ENSG00000106070,ENSG00000073803,ENSG00000138674,ENSG00000112394,ENSG00000156011,ENSG00000235568,ENSG00000171105,ENSG00000005844,ENSG00000038382,ENSG00000169180,ENSG00000064666,ENSG00000057019,ENSG00000168487,ENSG00000080603,ENSG00000169946,ENSG00000110880,ENSG00000169359,ENSG00000110395,ENSG00000124006,ENSG00000139540,ENSG00000100099,ENSG00000134815,ENSG00000135052,ENSG00000066468,ENSG00000048052,ENSG00000118322,ENSG00000114554,ENSG00000130208,ENSG00000153721,ENSG00000134909,ENSG00000111087,ENSG00000150938,ENSG00000155660,ENSG00000080493,ENSG00000184640,ENSG00000058091,ENSG00000025796,ENSG00000156886,ENSG00000136237,ENSG00000107957,ENSG00000110693,ENSG00000105419,ENSG00000100485,ENSG00000104671,ENSG00000173068,ENSG00000054611,ENSG00000169902,ENSG00000153317,ENSG00000105701,ENSG00000106049,ENSG00000173040,ENSG00000157399,ENSG00000077942,ENSG00000143970,ENSG00000076356,ENSG00000086991,ENSG00000111490,ENSG00000136040,ENSG00000115109,ENSG00000103196,ENSG00000099139,ENSG00000167460,ENSG00000116514,ENSG00000133392,ENSG00000136478,ENSG00000115548,ENSG00000100068,ENSG00000159216,ENSG00000013364,ENSG00000146555,ENSG00000099282,ENSG00000196526,ENSG00000143768,ENSG00000213639,ENSG00000169398,ENSG00000164292,ENSG00000124126,ENSG00000008294,ENSG00000134369,ENSG00000181031,ENSG00000170835,ENSG00000102189,ENSG00000106571,ENSG00000175535,ENSG00000135090,ENSG00000130529,ENSG00000168389,ENSG00000188158,ENSG00000075223,ENSG00000110925,ENSG00000105176,ENSG00000163947,ENSG00000197442,ENSG00000143376,ENSG00000170927,ENSG00000198162,ENSG00000100116,ENSG00000198380,ENSG00000119314,ENSG00000119686,ENSG00000130956,ENSG00000050748,ENSG00000100077,ENSG00000177463,ENSG00000072657,ENSG00000148516,ENSG00000095139,ENSG00000124788,ENSG00000130429,ENSG00000216490,ENSG00000131873,ENSG00000077782
biological_process	biological_process	204	94.00921659		GO:0008150	ENSG00000157107,ENSG00000162482,ENSG00000197102,ENSG00000154930,ENSG00000159692,ENSG00000143797,ENSG00000138119,ENSG00000115221,ENSG00000166501,ENSG00000219073,ENSG00000129566,ENSG00000112394,ENSG00000156011,ENSG00000235568,ENSG00000171105,ENSG00000118961,ENSG00000106070,ENSG00000073803,ENSG00000138674,ENSG00000038382,ENSG00000169180,ENSG00000174238,ENSG00000005844,ENSG00000148498,ENSG00000124571,ENSG00000187021,ENSG00000151693,ENSG00000147251,ENSG00000067057,ENSG00000067955,ENSG00000111269,ENSG00000116833,ENSG00000124164,ENSG00000090376,ENSG00000129515,ENSG00000243480,ENSG00000135525,ENSG00000157483,ENSG00000160094,ENSG00000139514,ENSG00000105329,ENSG00000160087,ENSG00000198879,ENSG00000033867,ENSG00000090861,ENSG00000145819,ENSG00000167004,ENSG00000079841,ENSG00000174939,ENSG00000198604,ENSG00000091542,ENSG00000136048,ENSG00000131236,ENSG00000091704,ENSG00000164073,ENSG00000124813,ENSG00000143811,ENSG00000138798,ENSG00000146433,ENSG00000106771,ENSG00000148935,ENSG00000164430,ENSG00000153002,ENSG00000114439,ENSG00000140575,ENSG00000169896,ENSG00000154310,ENSG00000031003,ENSG00000129116,ENSG00000105556,ENSG00000171517,ENSG00000175899,ENSG00000240038,ENSG00000140470,ENSG00000127152,ENSG00000071537,ENSG00000180530,ENSG00000141298,ENSG00000176390,ENSG00000197256,ENSG00000167601,ENSG00000077044,ENSG00000072954,ENSG00000124942,ENSG00000186635,ENSG00000169851,ENSG00000138640,ENSG00000175497,ENSG00000116350,ENSG00000142615,ENSG00000169862,ENSG00000110075,ENSG00000159128,ENSG00000101187,ENSG00000173575,ENSG00000213892,ENSG00000197442,ENSG00000163947,ENSG00000143376,ENSG00000170927,ENSG00000110925,ENSG00000075223,ENSG00000266200,ENSG00000105176,ENSG00000198380,ENSG00000119314,ENSG00000198162,ENSG00000100116,ENSG00000130956,ENSG00000050748,ENSG00000100077,ENSG00000119686,ENSG00000258539,ENSG00000130429,ENSG00000131873,ENSG00000216490,ENSG00000077782,ENSG00000177463,ENSG00000072657,ENSG00000148516,ENSG00000095139,ENSG00000124788,ENSG00000146555,ENSG00000100068,ENSG00000159216,ENSG00000013364,ENSG00000169398,ENSG00000164292,ENSG00000124126,ENSG00000152078,ENSG00000099282,ENSG00000196526,ENSG00000213639,ENSG00000143768,ENSG00000170835,ENSG00000102189,ENSG00000008294,ENSG00000142789,ENSG00000134369,ENSG00000181031,ENSG00000135090,ENSG00000130529,ENSG00000188158,ENSG00000168389,ENSG00000106571,ENSG00000215704,ENSG00000175535,ENSG00000100485,ENSG00000104671,ENSG00000054611,ENSG00000173068,ENSG00000169902,ENSG00000105419,ENSG00000106049,ENSG00000157399,ENSG00000173040,ENSG00000153317,ENSG00000105701,ENSG00000111490,ENSG00000086991,ENSG00000136040,ENSG00000115109,ENSG00000103196,ENSG00000099139,ENSG00000077942,ENSG00000143970,ENSG00000076356,ENSG00000133392,ENSG00000136478,ENSG00000115548,ENSG00000167460,ENSG00000116514,ENSG00000110880,ENSG00000169946,ENSG00000169359,ENSG00000110395,ENSG00000064666,ENSG00000057019,ENSG00000168487,ENSG00000080603,ENSG00000066468,ENSG00000048052,ENSG00000153721,ENSG00000114554,ENSG00000118322,ENSG00000130208,ENSG00000139540,ENSG00000124006,ENSG00000100099,ENSG00000134815,ENSG00000135052,ENSG00000080493,ENSG00000134909,ENSG00000155660,ENSG00000150938,ENSG00000111087,ENSG00000107957,ENSG00000110693,ENSG00000184640,ENSG00000058091,ENSG00000025796,ENSG00000156886,ENSG00000103534,ENSG00000136237

All the related genes (without duplicate)
ENSG00000166501,ENSG00000129566,ENSG00000079841,ENSG00000197442,ENSG00000054611,ENSG00000110925,ENSG00000131236,ENSG00000038382,ENSG00000169180,ENSG00000116514,ENSG00000111490,ENSG00000140575,ENSG00000073803,ENSG00000124164,ENSG00000124126,ENSG00000164292,ENSG00000048052,ENSG00000169398,ENSG00000099282,ENSG00000213639,ENSG00000110395,ENSG00000124571,ENSG00000110880,ENSG00000148498,ENSG00000175899,ENSG00000147251,ENSG00000180530,ENSG00000013364,ENSG00000105329,ENSG00000106571,ENSG00000160087,ENSG00000008294,ENSG00000181031,ENSG00000110075,ENSG00000090861,ENSG00000173575,ENSG00000215704,ENSG00000175535,ENSG00000184640,ENSG00000142789,ENSG00000175497,ENSG00000142615,ENSG00000128849,ENSG00000134815,ENSG00000243480,ENSG00000157483,ENSG00000118322,ENSG00000141298,ENSG00000080603,ENSG00000140470,ENSG00000168487,ENSG00000187021,ENSG00000240038,ENSG00000072657,ENSG00000133392,ENSG00000118961,ENSG00000153002,ENSG00000099139,ENSG00000219073,ENSG00000198162,ENSG00000157399,ENSG00000091704,ENSG00000266200,ENSG00000197102,ENSG00000167004,ENSG00000116350,ENSG00000130529,ENSG00000135090,ENSG00000058091,ENSG00000159216,ENSG00000067057,ENSG00000090376,ENSG00000066468,ENSG00000077044,ENSG00000167601,ENSG00000171105,ENSG00000100077,ENSG00000164430,ENSG00000086991,ENSG00000154310,ENSG00000077782,ENSG00000154930,ENSG0000124813,ENSG00000159692,ENSG00000119314,ENSG00000106049,ENSG00000130208,ENSG00000177463,ENSG00000167460,ENSG00000005844,ENSG00000148516,ENSG00000115548,ENSG00000131873,ENSG00000143970,ENSG00000169896,ENSG0000077942,ENSG00000153317,ENSG00000105701,ENSG00000138798,ENSG00000198604,ENSG00000091542,ENSG00000173068,ENSG00000156886,ENSG00000111087,ENSG00000186635,ENSG0000169851,ENSG00000102189,ENSG00000160094,ENSG00000116833,ENSG00000127152,ENSG00000169946,ENSG00000151693,ENSG00000143376,ENSG00000145819,ENSG00000157107,ENSG00000138119,ENSG00000100116,ENSG00000103196,ENSG00000171517,ENSG00000129515,ENSG00000134909,ENSG00000144426,ENSG00000107957,ENSG00000111269,ENSG00000067955,ENSG00000110693,ENSG00000105419,ENSG00000100485,ENSG00000124788,ENSG00000105556,ENSG00000119686,ENSG00000114439,ENSG00000155660,ENSG00000150938,ENSG00000143811,ENSG00000169902,ENSG00000174939,ENSG00000198380,ENSG00000162482,ENSG00000143797,ENSG00000216490,ENSG00000121964,ENSG00000050748,ENSG00000258539,ENSG00000136048,ENSG00000148935,ENSG00000129116,ENSG00000071537,ENSG00000176390,ENSG00000197256,ENSG00000124942,ENSG00000169862,ENSG00000213892,ENSG00000115221,ENSG00000106070,ENSG00000138674,ENSG00000138434,ENSG00000135525,ENSG00000139514,ENSG00000198879,ENSG00000173040,ENSG00000136040,ENSG00000115109,ENSG00000076356,ENSG00000057019,ENSG00000064666,ENSG00000150477,ENSG00000114554,ENSG00000153721,ENSG00000124006,ENSG00000100099,ENSG00000135052,ENSG00000080493,ENSG00000179715,ENSG00000025796,ENSG00000136237,ENSG00000163947,ENSG00000170927,ENSG00000075223,ENSG00000105176,ENSG00000130956,ENSG00000130429,ENSG00000138709,ENSG00000146555,ENSG00000100068,ENSG00000196526,ENSG00000164483,ENSG00000143768,ENSG00000136478,ENSG00000174238,ENSG00000146433,ENSG00000152078,ENSG00000168389,ENSG00000139540,ENSG00000169359,ENSG0000033867,ENSG00000156011,ENSG00000112394,ENSG00000235568,ENSG00000159128,ENSG00000101187,ENSG00000072954,ENSG00000138640,ENSG00000106771,ENSG00000031003,ENSG00000095139,ENSG00000188158,ENSG00000172765,ENSG00000164073,ENSG00000181826,ENSG00000075420,ENSG00000100731,ENSG00000103534,ENSG00000134369,ENSG00000196141,ENSG00000104671,ENSG00000170835

Circular RNA hsa_circRNA_0007334 is Predicted to Promote MMP7 and COL1A1 Expression by Functioning as miRNA Sponge in Pancreatic Ductal Adenocarcinoma

Jinghui Yang[1], Xianling Cong[3], Ming Ren[4], Hongyan Sun[3], Tao Liu [5], Gaoyang Chen[4], Qingyu Wang[4], Zhaoyan Li[4], Shan Yu[2*], Qiwei Yang[4,6*]

CircRNA Mirbase ID	CircRNA (Top) - miRNA (Bottom) pairing	Site Type	CircRNA Start	CircRNA End	3' pairing	local AU	position	TA	SPS	context+ s	context+ score percentile
hsa-miR-1283 (3' ... 5')	NNNNNNNNNNGUCAACUUUGUAGU	7mer-m8	7	13	too_close	too_close	too_close	too_close	too_close	too_close	NA
hsa-miR-1272 (3' ... 5')	CCUACUGUAUCAUGAUCAUCAUA	8mer-1a	197	204	0.003	0.011	-0.109	-0.009	0.064	-0.287	99
hsa-miR-1276 (3' ... 5')	AUUCAAGCAAAACUAGCUCUUUU	7mer-m8	80	86	0.003	-0.092	-0.051	0.012	0.018	-0.23	98
hsa-miR-621 (3' ... 5')	GCCAACUCUUUGCCUUGCUAGCA	8mer-1a	32	39	0.013	0.031	-0.106	-0.073	-0.073	-0.455	98
hsa-miR-548p (3' ... 5')	CUUUAUCUUGCACUUUUUUGCUU	7mer-m8	133	139	-0.016	-0.042	-0.05	0.03	0.058	-0.14	97
hsa-miR-330-3p (3' ... 5')	UAUCUUGCACUUUUUUGCUUUGG	7mer-m8	136	142	-0.007	-0.056	-0.05	0.02	0.023	-0.19	96
hsa-miR-604 (3' ... 5')	CUCUUUGCCUUGCUAGCAGCCAU	7mer-1a	37	43	-0.002	-0.006	-0.046	-0.002	-0.104	-0.234	95
hsa-miR-647 (3' ... 5')	CUCUUUGCCUUGCUAGCAGCCAU	7mer-m8	37	43	0.012	-0.056	-0.057	0.01	-0.084	-0.295	95
hsa-miR-433 (3' ... 5')	GGAAUUUCCUACUGUAUCAUGAU	7mer-m8	190	196	0.03	-0.035	-0.058	0.002	0.043	-0.138	94
hsa-miR-571 (3' ... 5')	CAACUUUGUAGUGUGCCAACUCU	7mer-m8	18	24	0.003	0.003	-0.059	-0.013	-0.018	-0.204	94
hsa-miR-144 (3' ... 5')	CAAAGUGGAAUUUCCUACUGUAU	7mer-1a	184	190	0.001	-0.008	-0.046	0.013	0.015	-0.099	92
hsa-miR-577 (3' ... 5')	CAUUUGGUUUCGAACUUAUCUAC	7mer-1a	57	63	0.004	-0.005	-0.044	0.015	0.066	-0.038	92
hsa-miR-577 (3' ... 5')	CUACCCUUUUGGGCCUUUAUCUU	7mer-m8	119	125	0.003	0.055	-0.048	0.015	0.095	0	87
hsa-miR-515-5p (3' ... 5')	UGAUCAUCAUAGGAGUGGAGAAC	7mer-1a	209	215	0.008	0.005	-0.049	0.023	-0.034	-0.121	86
hsa-miR-1322 (3' ... 5')	CUACUGUAUCAUGAUCAUCAUAG	7mer-1a	198	204	-0.009	0.014	-0.048	-0.016	0.033	-0.1	84

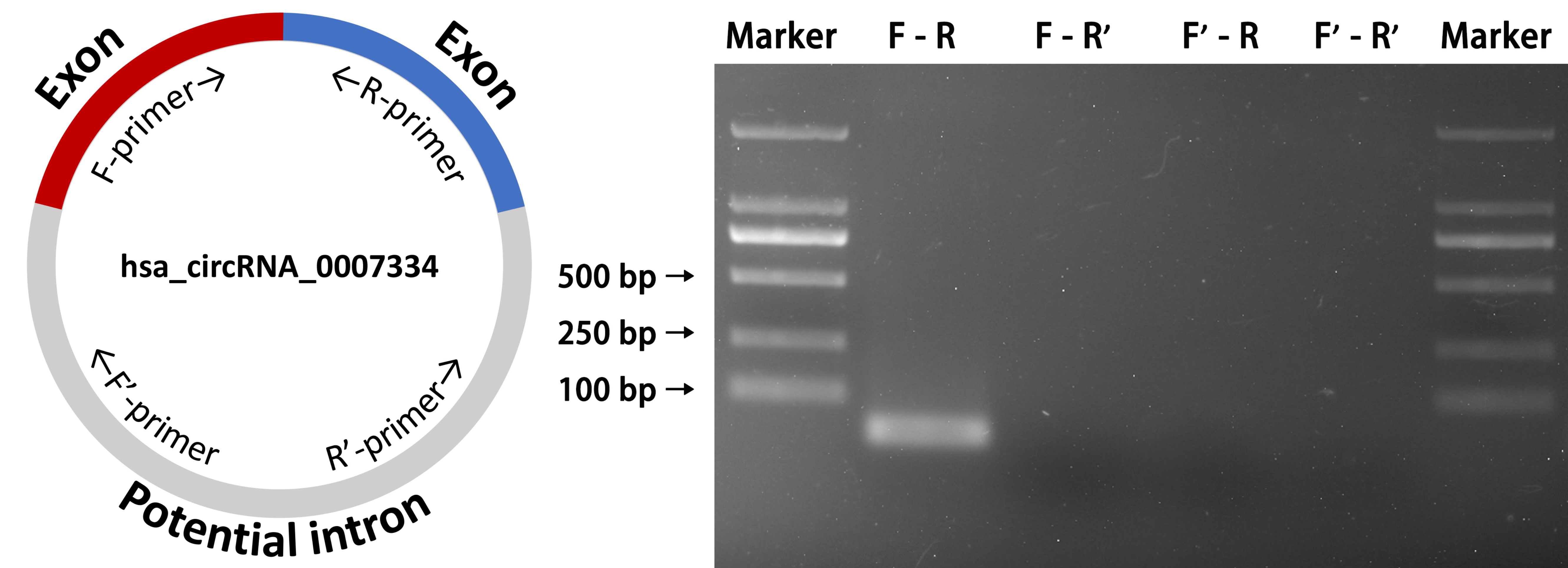
Exon
Exon
F-primer
R-primer
hsa_circRNA_0007334
F'-primer
R'-primer
Potential intron
500 bp
250 bp
100 bp
Marker
F - R
F - R'
F' - R
F' - R'
Marker
